# Pediatric Respiratory and Enteric Virus Acquisition and Immunogenesis in US Mothers and Children Aged 0-2: PREVAIL Cohort Study

**DOI:** 10.2196/22222

**Published:** 2021-02-12

**Authors:** Ardythe L Morrow, Mary A Staat, Emily A DeFranco, Monica M McNeal, Allison R Cline, Shannon C Conrey, Elizabeth P Schlaudecker, Alexandra M Piasecki, Rachel M Burke, Liang Niu, Aron J Hall, Michael D Bowen, Susan I Gerber, Gayle E Langley, Natalie J Thornburg, Angela P Campbell, Jan Vinjé, Umesh D Parashar, Daniel C Payne

**Affiliations:** 1 Department of Environmental and Public Health Sciences, Division of Epidemiology University of Cincinnati College of Medicine Cincinnati, OH United States; 2 Division of Infectious Diseases Cincinnati Children's Hospital Medical Center Cincinnati, OH United States; 3 Department of Pediatrics University of Cincinnati College of Medicine Cincinnati, OH United States; 4 Department of Obstetrics and Gynecology University of Cincinnati College of Medicine Cincinnati, OH United States; 5 Division of Viral Diseases National Center for Immunization and Respiratory Disease Centers for Disease Control and Prevention Atlanta, GA United States; 6 Department of Environmental and Public Health Sciences, Division of Biostatistics and Bioinformatics University of Cincinnati College of Medicine Cincinnati, OH United States; 7 Division of Foodborne, Waterborne, and Environmental Diseases National Center for Emerging and Zoonotic Infectious Diseases Centers for Disease Control and Prevention Atlanta, GA United States

**Keywords:** birth cohort, RSV, influenza, rotavirus, norovirus, vaccines, vaccine effectiveness, immunology, pediatrics

## Abstract

**Background:**

Acute gastroenteritis (AGE) and acute respiratory infections (ARIs) cause significant pediatric morbidity and mortality. Developing childhood vaccines against major enteric and respiratory pathogens should be guided by the natural history of infection and acquired immunity. The United States currently lacks contemporary birth cohort data to guide vaccine development.

**Objective:**

The PREVAIL (Pediatric Respiratory and Enteric Virus Acquisition and Immunogenesis Longitudinal) Cohort study was undertaken to define the natural history of infection and immune response to major pathogens causing AGE and ARI in US children.

**Methods:**

Mothers in Cincinnati, Ohio, were enrolled in their third trimester of pregnancy, with intensive child follow-up to 2 years. Blood samples were obtained from children at birth (cord), 6 weeks, and 6, 12, 18, and 24 months. Whole stool specimens and midturbinate nasal swabs were collected weekly and tested by multipathogen molecular assays. Saliva, meconium, maternal blood, and milk samples were also collected. AGE (≥3 loose or watery stools or ≥1 vomiting episode within 24 hours) and ARI (cough or fever) cases were documented by weekly cell phone surveys to mothers via automated SMS text messaging and review of medical records. Immunization records were obtained from registries and providers. follow-up ended in October 2020. Pathogen-specific infections are defined by a PCR-positive sample or rise in serum antibody.

**Results:**

Of the 245 enrolled mother–child pairs, 51.8% (n=127) were White, 43.3% (n=106) Black, 55.9% (n=137) publicly insured, and 86.5% (n=212) initiated breastfeeding. Blood collection was 100.0% for mothers (n=245) and 85.7% for umbilical cord (n=210). A total of 194/245 (79.2%) mother–child pairs were compliant based on participation in at least 70% (≥71/102 study weeks) of child-weeks and providing 70% or more of weekly samples during that time, or blood samples at 18 or 24 months. Compliant participants (n=194) had 71.0% median nasal swab collection (IQR 30.0%-90.5%), with 98.5% (191/194) providing either an 18- or 24-month blood sample; median response to weekly SMS text message surveys was 95.1% (IQR 76.5%-100%). Compliant mothers reported 2.0 AGE and 4.5 ARI cases per child-year, of which 25.5% (160/627) and 38.06% (486/1277) of cases, respectively, were medically attended; 0.5% of AGE (3/627) and 0.55% of ARI (7/1277) cases were hospitalized.

**Conclusions:**

The PREVAIL Cohort demonstrates intensive follow-up to document the natural history of enteric and respiratory infections and immunity in children 0-2 years of age in the United States and will contribute unique data to guide vaccine recommendations. Testing for pathogens and antibodies is ongoing.

**International Registered Report Identifier (IRRID):**

RR1-10.2196/22222

## Introduction

Acute gastroenteritis (AGE) and acute respiratory infection (ARI) remain major causes of morbidity and mortality in young children worldwide. AGE is estimated to cause more than 1.5 million deaths globally among children under 5 years of age each year [1-3]. Rotavirus remains a major cause of global pediatric disease burden, particularly among unvaccinated children [4,5], but in the United States norovirus has become the leading cause of AGE since the introduction of widespread childhood rotavirus vaccination [4]. Norovirus causes approximately 20% of all AGE cases among US children visiting emergency rooms and admitted to hospital [4,6-9], and an estimated population-wide annual burden of 19-21 million AGE cases, including 56,000-71,000 hospitalizations and 570-800 deaths [10,11]. ARI is estimated to cause 5 million deaths globally among children under 5 years each year [12-16]. Respiratory syncytial virus (RSV) is the most common cause of viral lower respiratory tract illness in young children, responsible for 58,000 hospitalizations and 2 million outpatient visits in children under 5 years of age in the United States [17]. In the United States and Australia, 2% of children are hospitalized with RSV infection before their first birthday [17-19].

Given the significant burden of disease that norovirus and RSV cause in young children, these pathogens are high priority targets for vaccine development. However, there are significant obstacles to this effort, and greater understanding of their natural history and immune responses is critical. For example, norovirus GII.4 strains undergo rapid evolution, resulting in emergent immune escape variants every 2-5 years which replace previous predominant strains. RSV infections do not result in sterilizing immunity and correlates of protection are not completely understood. Maternal vaccination is under investigation as a potential strategy to reduce the burden of RSV disease in young infants, but deeper understanding is needed of maternal–infant immune transfer and infant acquisition of immunity [20-25].

Rotavirus vaccines have been in use since 2006 in the United States (RotaTeq, Merck and Co.; and Rotarix, GlaxoSmithKline Biologicals). While efforts to better understand vaccine performance have been fruitful, factors that influence rotavirus vaccine effectiveness in the postvaccine era, as well as the persistence and transmission of rotavirus, now need to be considered for both mother and child. These factors were not possible to have been assessed in rotavirus vaccine prelicensure clinical trials, which occurred amidst the prevaccine global setting of persistent and population-wide rotavirus exposures causing regular immunologic challenges. Existing vaccines to prevent influenza (including live attenuated influenza vaccine and inactivated influenza vaccine) are not recommended for infants under 6 months of age, so understanding the impact of maternal–infant immunologic transfer in utero and via breastmilk is important to assess for infants too young to be protected by vaccination. Even for older infants and children, it is important to have a better understanding of the complementary and aggregate roles of antibody protection via breastmilk antibodies and naturally acquired immunity from a child’s first and second annual influenza seasons; deeper knowledge of this dynamic could improve our knowledge of how the vaccines could be optimized.

Understanding of the early natural history of infection and immune response requires intensive birth cohort studies that identify both symptomatic and asymptomatic infections and the pattern of immunity that develops to natural infections within each child. A well-conducted birth cohort can determine the specificity of immune response to natural infections, including homologous or heterologous protection, and other information critical to vaccine development. This concept is exemplified by a classic birth cohort study conducted in Mexico that defined rotavirus natural history and immunity [26] and provided the epidemiological framework required to develop the currently licensed rotavirus vaccines. Because of high cost and logistical limitations, there has been a paucity of birth cohort studies of natural infection and immunity in the United States in recent decades. Most cohort studies have been conducted in low-resource countries where infectious disease rates are high and research staff can document the health status of children through frequent home visits. The studies from low-resource countries have been invaluable to public health worldwide. However, the lack of such studies in the United States continues an unfortunate knowledge gap regarding the population burden of disease and the potential effectiveness of childhood vaccines in the US context.

To address this gap in public health data, the Centers for Disease Control and Prevention (CDC) sponsored the PREVAIL (Pediatric Respiratory and Enteric Virus Acquisition and Immunogenesis Longitudinal) Cohort [27]. The PREVAIL Cohort is a prospective, observational study of mother–child pairs residing in the Cincinnati, Ohio region. The study was designed to determine the natural history of endemic enteric and respiratory viral infections (norovirus, RSV, rotavirus, and influenza virus) and immune response to those infections in children. The aims of PREVAIL were to design and enact an intensive data and sample collection methodology for a birth cohort study of norovirus, rotavirus, RSV, and influenza virus infections and their immune responses in the United States. Novel elements of the design include collection of prospective samples enabled by electronic medical records alerts and trained hospital staff; weekly stool and nasal swab collections from study infants by their mothers, enabled by a courier service; as well as weekly data collection from mothers enabled by automated SMS text message surveys. In this paper we present the overall study design and describe our initial outcomes: maternal compliance with the study’s intensive sample and data collection schedule and maternally reported AGE and ARI incidence in the cohort. Our experience demonstrates the feasibility of birth cohort studies in the United States enabled by available infrastructure and technologies.

## Methods

### Enrollment and Follow-Up

**Figure 1:**
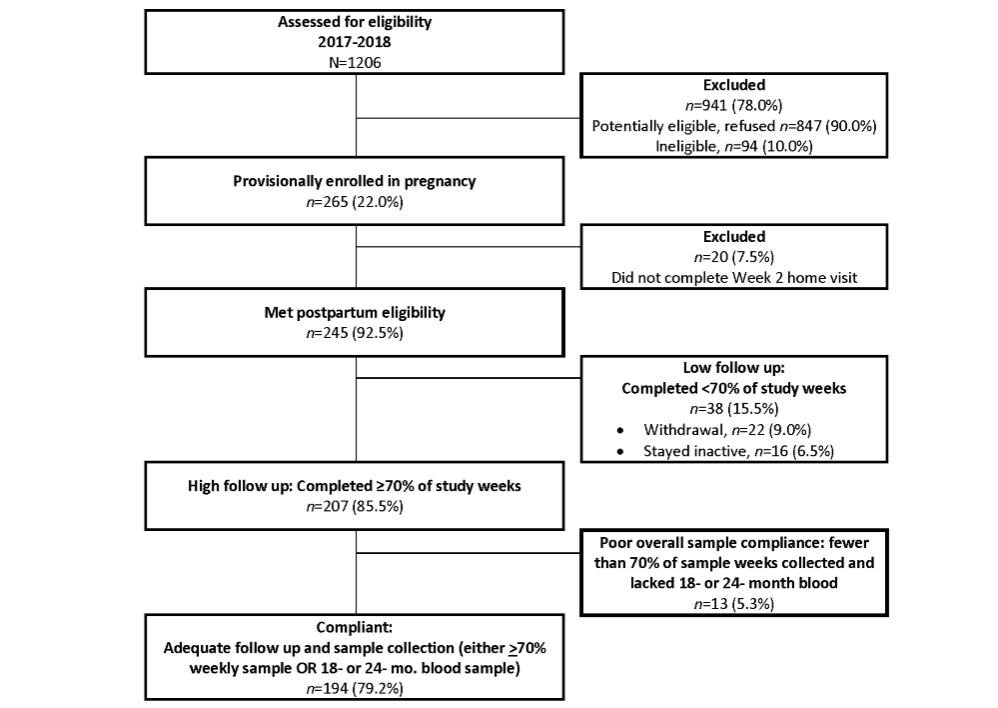
Participant enrollment flow chart, PREVAIL Study, STROBE flow chart. STROBE, Strengthening the Reporting of Observational Studies in Epidemiology [28].

Launched in March 2017, the PREVAIL Cohort included generally healthy mother and child pairs followed actively from the third trimester of pregnancy until the child’s second birthday. Recruitment was conducted in obstetrical clinics associated with the 2 study birth hospitals in Cincinnati, Ohio (Figure 1): University of Cincinnati Medical Center (UCMC) and The Christ Hospital (TCH). Target enrollment was set as 265 pregnant mothers in the last trimester of pregnancy and at least 240 eligible mother–infant pairs at postpartum week 2. Target sample size was based on 80% power, α=.05, and 2-sided tests of hypothesis to detect protection against repeat infections with norovirus or RSV. The target sample size was more than sufficient to provide robust estimation of AGE and ARI cases reported here. This study was reviewed and approved by institutional review boards at the CDC, Cincinnati Children’s Hospital Medical Center (CCHMC), and the hospitals where maternal enrollment and delivery occurred.

Mothers were screened for potential eligibility using medical records and approached in the last trimester during an outpatient obstetric visit. Eligible pregnant women at or after 34 weeks of gestation were invited to participate. Those who elected to enroll completed a written, informed consent. Predelivery inclusion criteria for enrollment included singleton pregnancy, maternal age of 18 years or more, planned delivery at either study hospital, and a cell phone that could be used for SMS text messaging. The cell phone criterion provided a means for close communication with study mothers. Predelivery exclusion criteria were living more than 20 mi from the birth hospital, illicit drug use, and HIV infection. Exclusion based on distance was due to the logistical requirements of weekly sample transport after the child’s birth. Final inclusion in the postnatal follow-up portion of the study was contingent upon maternal delivery of a singleton, liveborn infant, lack of a major congenital anomaly, and active participation in the week 2 home visit. Enrollment was completed in July 2018.

Study mothers were trained in study procedures during the week 2 home visit by a research nurse, and reminders were provided during subsequent research visits. Maternal training emphasized techniques for sample collection, initiating courier pick-up, response to text questionnaires, and taking the child’s temperature during illness. Mothers were provided with a notebook to provide specific guidance on study procedures, and information on how to call the research staff and the courier service. Each mother was provided with a thermometer and taught how to safely obtain a rectal temperature in infants up to 6 months, and an axillary measurement after 6 months.

Participants under follow-up were monitored and classified as withdrawn, active, or inactive on an ongoing basis. Withdrawals were defined as participants who formally notified study staff that they no longer wished to continue participation. Inactive participants were defined as those who passively ceased meaningful participation, based on failing to attend a study clinic visit and cessation of all sample collection or SMS text message survey responses for at least eight consecutive weeks. Participation was closely monitored, and when several weeks of inactivity were observed, study staff reached out and made every effort to engage participants in the study.

To acknowledge the significant time and effort required of study mothers, we established a compensation schedule that included each study visit and each sample type. Compensation was provided using the ClinCard system [29], with funds loaded electronically onto mother’s study card once a month. Mothers who completed all study procedures, from pregnancy through the infant’s second birthday, were given a total compensation of up to US $1500. However, actual compensation varied, depending on adherence to the study procedures. To optimize weekly sample collection, we instituted a reward system of an additional monthly compensation if monthly sample compliance was 75% or more (ie, if at least three stool samples and three nasal swabs were received out of the four expected for each of the sample types). ClinCard payments were made on a rolling 4-week cycle for completion of weekly study requirements. Participants with sample compliance that fell below 75% were contacted by study staff to encourage participation, but there were no consequences for noncompliance other than loss of potential compensation.

### Data and Sample Collection and Management

**Figure 2:**
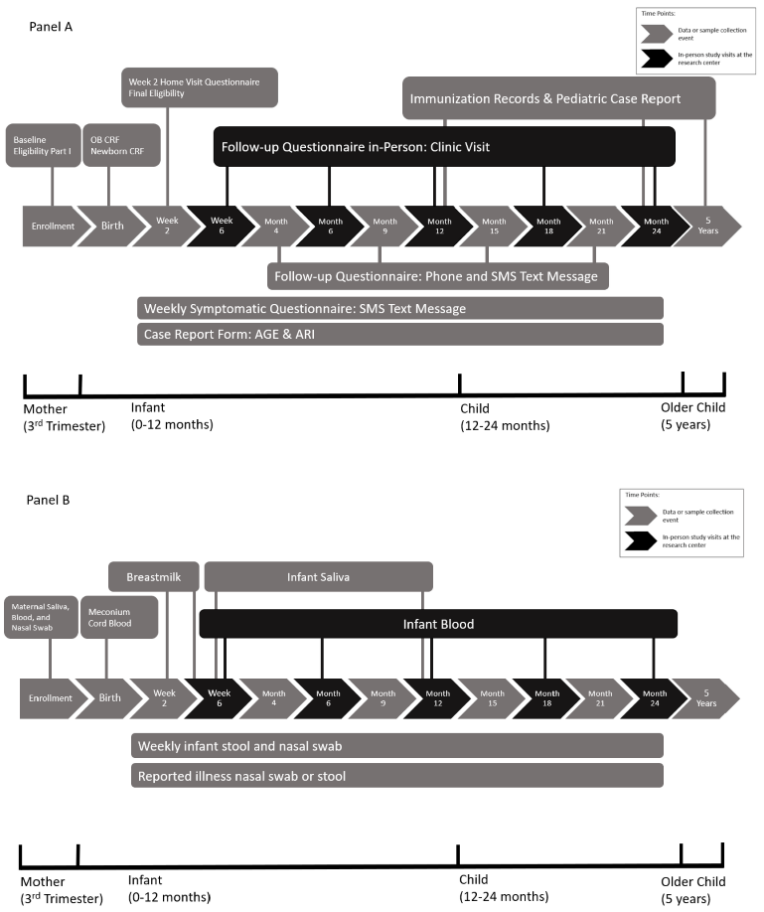
PREVAIL study timelines. (A) Data collection; (B) sample collection. AGE: acute gastroenteritis; ARI: acute respiratory infection; CRF: case report form.

The study data system (Figure 2A) included comprehensive standardized questionnaires administered in-person during research visits, immunization and medical record abstraction, and automated SMS text message surveys administered weekly or periodically. The questionnaires applied throughout the study (Multimedia Appendices 1-6) captured infectious disease risk factors such as sociodemographic factors, immunization history, household composition, childcare and breastfeeding, as well as related child health factors such as nutrition and sleep practices.

During the prenatal enrollment visit, a baseline questionnaire was administered to the mother regarding her household, health, and immunization history. Subsequent research visits were held at postnatal weeks 2 and 6 and months 6, 12, 18, and 24. Participants were required to complete the week 2 study visit within a 10-day window, and the week 6 to month 24 visits anytime during a 12-week window beginning with the due date.

The week 2 visit was conducted in the mother’s home. The remaining postnatal research visits were conducted in the clinic and included measurement of child weight and length. Between research clinic visits, a brief questionnaire was administered by phone (month 4) or through automated cell-phone SMS text messaging (months 9, 15, and 18) to determine any change in time-varying covariates such as breastfeeding or childcare.

Weekly reporting of symptoms in study children was conducted by automated cell phone SMS text messaging. The system used REDCap (Research Electronic Data Capture) [30,31], a secure, web-based software platform designed to support data capture and management for research studies. REDCap has a plugin to connect to the Twilio system [32], which supports fully automated delivery of the survey to enrolled mothers via their personal cell phone. Responses were automatically captured in the REDCap database without need for further data entry.

Affirmative maternal responses to weekly symptom surveys triggered a follow-up survey to ascertain type and severity of symptoms, illness start date, and whether the illness was ongoing. Once the child was reported as no longer symptomatic, an automated follow-up survey requested additional information on the episode, including the date ended, the location of any medically attended visits, and administration of any medications.

Electronic hospital records were systematically abstracted for pregnancy and perinatal histories of the mother and infant. The medical records at Cincinnati Children’s Hospital, which includes its emergency room and affiliated clinics, were abstracted for all study children. Medical records are also currently being requested from all other pediatric providers. Record abstraction focused on AGE, ARI, and other medically relevant illnesses or conditions (eg, asthma, sickle cell anemia) and prescription of antibacterial or antiviral medications. Pediatric and maternal immunization records were obtained from Ohio and Kentucky immunization registries and identified health care providers. Maternal immunization records for influenza were also sought from employers.

Study data were entered directly into the REDCap database either by study staff at research visits via an internet-enabled tablet or by mothers responding to weekly SMS text message surveys. Maternal adherence to the protocol was routinely tracked for clinic visits and weekly sample and data collection. The REDCap database system included logic checks at the point of data capture. A data team comprising CCHMC and CDC staff systematically reviewed completed questionnaires to ensure completion of missing data and verify or correct improbable values.

Samples collected from study participants (Figure 2B) included midturbinate nasal swab, whole stool, blood, saliva, and milk. The pregnancy enrollment visit included collection of a maternal midturbinate nasal swab, saliva, and blood. Birth samples included cord blood collected at delivery and meconium stool samples collected during the delivery hospitalization. At the university birth hospital (UCMC), study mothers were identified in the hospital electronic medical records system as PREVAIL participants. Subsequently, when mothers were admitted for delivery, the UCMC electronic medical record system sent an automated phone SMS text message to on-call study staff notifying them of the hospital admission, and the research staff coordinated with the hospital delivery team to collect cord blood at birth. At the community birth hospital (TCH), there were no 24-hour research staff; instead, several labor-intensive procedures were instituted: TCH clinical staff were trained every few months on key study procedures, and reminded to collect cord bloods on study mothers, with posted signs prompting nurses to ask about the mother’s study involvement; furthermore, mothers delivering at TCH were instructed to inform labor and delivery nurses that they were enrolled in the PREVAIL study. Regardless of study hospital, all study mothers were asked to collect their infant’s meconium sample after delivery and ask their clinical nurses to keep the sample refrigerated until courier pick-up for delivery to the study laboratory.

At the week 2 home visit, mothers were trained in the remaining study procedures by the study nurse and provided with materials for sample collection and an organizer with instructions and materials. This training took about 45 minutes. Because of the importance and novelty of the instructions to the mother, key messages were repeated at the week 6 research visit, and close contact was maintained by text or phone to assist mothers who initially struggled with sample collection or weekly surveys.

Mothers were asked to collect a soiled diaper and a nasal swab from their child between Saturday and Wednesday of each week, with additional stool or nasal samples during illness. Stool collection involved having mothers wrap and place their child’s soiled diaper into a resealable plastic bag, label it with the date of collection, and place the sealed bag into a disposable pouch designed for temperature control. Mothers were shown how to collect a midturbinate nasal swab using a flocked swab (Copan Diagnostics, Inc.) placed inside the child’s nostril, not very far up, and gently rotate the swab in place. The swab was then placed into the vial containing BD Universal Viral Transport medium (Becton, Dickinson, and Co., Franklin Lakes, New Jersey), which was labeled with the collection date. The nasal swab vial was then placed into the pouch, sealed, and kept in the home refrigerator until time to contact the courier.

Stool and nasal swabs were sent to the study laboratory via courier within 1 to 2 days of collection. Mothers contacted the courier to initiate sample pick-up and placed samples into a small hard-sided cooler with an ice pack. The cooler was labeled for the courier and typically placed outside the door of the mother’s residence. The courier service delivered samples from study homes to the study laboratory within a 4-hour window, trained its employees in study procedures, and contacted the study coordinator whenever sample collection issues arose. In the study laboratory, sample management and biobanking followed standardized procedures, including bar coding and entry of data into a professional software system for inventory management.

### Study Outcomes

Study outcomes were maternal compliance with the study procedures and the incidence rates of AGE and ARI. Compliance was defined for this study as achieving at least 70% of intended follow-up duration (≥71/102 weeks) and at least one of the following: 70% of weekly sample collection during their period of activity in the study or a blood sample collected at 18 or 24 months. This level of compliance was considered adequate to identify infections in the first and second years of life. However, we also analyzed the impact of changing our standard for compliance to 80% of study weeks (≥81/102) and 80% of weekly sample collections or blood collection at 18 or 24 months; because we found that only 2 participants changed categories from compliant to noncompliant, the cut point was maintained as 70% to be inclusive. All study samples were maintained for the periods for which mothers participated, including those who did not meet compliance goals. All participant data will remain available for analysis and will be selected or excluded based on individual study requirements.

An episode of AGE was defined as 3 or more loose or watery stools or 1 or more vomiting episodes within 24 hours at any time in the previous week. An episode of AGE was considered to have ended when 2 or more consecutive asymptomatic days occurred. Severity of AGE is scored using the modified Vesikari scale [33].

An episode of ARI is defined as the presence of cough or fever (temperature ≥38.0°C, rectal; ≥37.0°C, axillary) at any time in the previous week. When fever was reported, study staff asked the mother for the child’s temperature, and the method for measuring temperature. Severity of ARI was based on the highest medical care sought for the child: hospital or emergency department use was considered moderate to severe.

Incidence rates of AGE and ARI were calculated as the number of cases reported divided by the number of weeks of SMS text message survey reporting by study mothers subtracting the weeks that the child was symptomatic for either AGE or ARI, multiplied by 52 to report cases per child-year and by 100 to report cases per 100 child-weeks.

## Results

### Enrollment and Follow-Up

Based on review of obstetrical records, a total of 1206 pregnant women were identified as potentially eligible and approached about the study (Figure 1). The 1206 screened patients were reported in medical records to be 44.78% White (n=540), 47.60% Black (n=574), and 7.63% other or unknown (n=92). On the day of initial screening contact, 8.21% (n=99) of mothers were identified as ineligible, 21.39% (n=258) refused participation, 15.42% (n=186) completed enrollment, and 54.81% (n=661) refused enrollment at that time but invited recontact about the study. Of the 192 mothers who provided a reason for nonparticipation, the primary reasons for refusal were lack of interest (n=126, 65.6%), not enough time (n=31, 16.1%), and the blood draws (n=14, 7.3%). Of the 661 women who refused immediate enrollment but allowed recontact, 79 (12.0%) were subsequently enrolled. Altogether, 265/1206 (22.0%) mothers who were screened consented to participate, which fully met our target enrollment goal. Enrollment in pregnancy was nearly evenly divided between the 2 birth hospitals.

Of the 265 mothers included in pregnancy, 245 (92.5%) met the postpartum criteria and completed enrollment (Figure 1), which modestly exceeded our planned enrollment goal. The 245 mother–child pairs who completed postpartum enrollment into the study are described in Table 1. Mothers ranged from 18 to 45 years of age. At the time of enrollment, 55.9% (n=137) were publicly insured, 43.3% (n=106) were privately insured, and 0.8% (n=2) were self-pay. Reported household income was less than US $25,000 for nearly one-third of study households (n=78), with the highest income category above US $100,000 for nearly one-quarter of study households (n=58). Nearly half of study mothers were married (n=118), with an additional 18.4% (n=45) of women unmarried but residing with their partner. As much as 4.5% (n=11) of infants were born late preterm, at 35 or 36 weeks of age. Most mothers reported their race as White (n=127, 51.8%) or Black (n=106, 43.3%); the remaining mothers identified themselves as biracial, Asian, or other. This racial distribution was like that identified at initial participant screening.

**Table 1:** Characteristics of the mother–child pairs in the PREVAIL Cohort.

Characteristics			All subjects (N=245), n (%)		Compliant (N=194), n (%)		Noncompliant (N=51), n (%)		*P* value
**Maternal age**									<.001
	18-24 years	50 (20.4)		29 (14.9)		21 (41.2)			
	25-34 years	155 (63.3)		132 (68.0)		23 (45.1)			
	≥35 years	40 (16.3)		33 (17.0)		7 (13.7)			
**Parity**									.470
	1	93 (38.0)		74 (38.1)		19 (37.3)			
	2	67 (27.3)		56 (28.9)		11 (21.6)			
	>3	85 (34.7)		64 (33.0)		21 (41.2)			
**Maternal race**									.630
	Black	106 (43.3)		82 (42.3)		24 (47.1)			
	White	127 (51.8)		103 (53.1)		24 (47.1)			
	Biracial	5 (2.0)		4 (2.1)		1 (2.0)			
	Asian and unknown	7 (2.9)		5 (2.6)		2 (3.9)			
**Insurance**									.008
	Public	137 (55.9)		99 (51.0)		38 (74.5)			
	Private	106 (43.3)		93 (47.9)		13 (25.5)			
	Unknown	2 (0.8)		2 (1.0)		0 (0.0)			
**Maternal education**									.012
	Less than high school	22 (9.0)		16 (8.2)		6 (11.8)			
	High-school graduate	93 (38.0)		65 (33.5)		28 (54.9)			
	Associate/trade	35 (14.3)		29 (14.9)		6 (11.8)			
	College graduate	95 (38.8)		84 (43.3)		11 (21.6)			
**Household income (annual)**									.001
	<US $25,000	78 (31.8)		57 (29.4)		21 (41.2)			
	US $25,000-US $49,999	49 (20.0)		36 (18.6)		13 (25.5)			
	US $50,000-US $99,999	48 (19.6)		45 (23.2)		3 (5.9)			
	US $100,000 and above	58 (23.7)		50 (25.8)		8 (15.7)			
	Unknown	12 (4.9)		6 (3.1)		6 (11.8)			
**Marital status**									.008
	Married	118 (48.2)		103 (53.1)		15 (29.4)			
	Lives with partner	45 (18.4)		31 (16.0)		14 (27.5)			
	Divorced, separated, or single	82 (33.5)		60 (30.9)		22 (43.1)			
**Delivery mode**									.260
	Vaginal	151 (61.6)		116 (59.8)		35 (68.6)			
	Cesarean	94 (38.4)		78 (40.2)		16 (31.4)			
**Infant gestational age at birth**									.710
	35-36 weeks	11 (4.5)		9 (4.6)		2 (3.9)			
	37 weeks	44 (18.0)		37 (19.1)		7 (13.7)			
	38-42 weeks	190 (77.6)		148 (76.3)		42 (82.4)			
**Number of adults in** **household**									>.99
	2 or more adults	189 (77.1)		149 (76.8)		40 (78.4)			
	1 adult	56 (22.9)		45 (23.2)		11 (21.6)			
**Total number of persons in** **household**									.310
	2 persons	14 (5.7)		11 (5.7)		3 (5.9)			
	3 or 4 persons	146 (59.6)		121 (62.4)		25 (49.0)			
	5 or 6 persons	61 (24.9)		44 (22.7)		17 (33.3)			
	>6 persons	24 (9.8)		18 (9.3)		6 (11.8)			
**Breastfeeding**									.17
	Initiated	212 (86.5)		171 (88.1)		41 (80.4)			
	Did not initiate	33 (13.5)		23 (11.9)		10 (19.6)			

**Figure 3:**
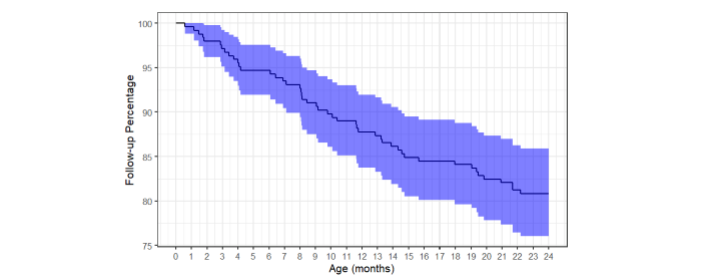
Survival curve of 245 study participants and their participation over the 2-year follow-up period. The curve represents cumulative loss of 47 infants who withdrew or became and remained inactive prior to the final scheduled visit at 2 years.

The 245 mother–child pairs enrolled in the study contributed 433.6 child-years of follow-up. Of these 245 mother–child pairs, a total of 47 (19.2%) were ever withdrawn or remained inactive in the study, of whom 38 were lost to follow-up prior to week 71 of the study; 9 became inactive between week 71 and the 2-year study visit. Loss to follow-up occurred fairly evenly from birth to 2 years of age (Figure 3).

### Data and Sample Collection and Management

Overall, there was generally high adherence to completion of questionnaires, research visits, weekly data and sample collection, and completion of scheduled blood draws. A total of 1246 study visits were performed, 18,183 weekly SMS text message surveys were completed, and 13,809 weekly stool samples, 14,361 weekly nasal samples, and 1,176 infant blood samples were collected. Excluding only children who formally withdrew from the study by the time of each research visit, adherence to completion of study visits was 96.7% (233/241) at week 6 and 79.4% (177/223) at month 24. Lower adherence to coming into the research clinic for a blood draw for the 2-year study visit was a combination of time in the study or age, but also the COVID-19 pandemic occurred in Cincinnati when the last group of study participants were approaching the final study visit. For this last group, in-person study visits had to be postponed for a few months, at which point some indicated that they no longer wished to conduct the 2-year in-person study visit. We estimate a loss of as many as 15 participants from the 2-year blood draw due to interruption of human research by the pandemic.

Among all 245 enrolled participants, blood was obtained from all study mothers at the time of enrollment in pregnancy, and 88.6% (217/245) collection of umbilical cord blood samples was achieved from the birth hospitals. Blood sample collection was 80% or above throughout the first year of life but declined over time except in those identified as compliant (Table 2). During postnatal research visits, blood sample collection failed about 5%-10% of the time, resulting in rescheduling blood draws whenever possible.

**Table 2:** Follow-up, sample, and data collection.

Study parameter			All enrolled (N=245)		Compliant (N=194)		
**Follow-up time**							
	Total child-weeks	22,549		20,111			
	Total child-years	433.6		386.8			
	Withdrew or remained inactive, n (%)	47 (19.2)		4 (2.1)			
**Blood collection, n (%)**							
	Maternal blood, third trimester	245 (100.0)		194 (100.0)			
	Umbilical cord blood	210 (85.7)		164 (84.5)			
	Infant week 6	213 (86.9)		175 (90.2)			
	Infant month 6	205 (83.7)		178 (91.8)			
	Infant month 12	197 (80.4)		185 (95.4)			
	Infant month 18	186 (75.9)		184 (94.8)			
	Infant month 24	165 (67.3)		164 (84.5)			
**Weekly data and sample (percentage of study weeks), median (IQR)**							
	SMS text message survey response	91.8 (69.9-100.0)		95.1 (76.6-100.0)			
	Stool samples collected	62.1 (20.0-89.3)		71.8 (29.1-100.0)			
	Nasal swabs collected	61.0 (21.9-87.6)		71.0 (30.0-90.5)			

Sample collection rates were also robust for other sample types. Saliva was collected from 234/245 (95.5%) study children for secretor status determination. In addition, of the 180 mothers who reported breastfeeding at the week 2 home visit, all provided at least one milk sample. Collection of cord blood was high but differed by hospital. The perinatal research system at UCMC produced a higher cord blood collection rate (114 of 125 expected samples, 91.2%) than the attentive but more ad hoc approach required for cord collection from TCH (96 of 120 expected, 80.0%, *P*=.017). By contrast, the collection of meconium, which depended upon study mothers, was obtained by 232 of 245 mothers (94.7%), and not influenced by birth hospital.

### Study Outcomes

Of the 245 mother–child pairs enrolled in the study, 194 (79.2%) were defined as compliant, having achieved follow-up of 70% (≥71/102) or more of study weeks, and either collection of 70% or more of weekly samples during their period of activity or having a blood collection at 18 or 24 months of age. Compliant mother–child pairs contributed 386.8 of the 433.6 child-years (89.2% of the total follow-up time in the study; Table 2). Mothers who were compliant in the study were significantly (*P*<.05) more likely than noncompliant mothers to be older, privately insured, college educated, have higher income, and married (Table 1). Nevertheless, the characteristics of the 194 mothers defined as adequately compliant over the course of the study remained generally representative of our study population.

Among the 194 compliant mother–child pairs, blood sample collections ranged from 84.5% to 95.4% of children at each scheduled time (Table 2). Median weekly response to SMS text message surveys to report AGE or ARI in the study child was 95.1% (IQR 76.5%-100.0%) of active weeks (Table 2), though individual response to weekly SMS text message surveys was variable. Median weekly sample collection was 71% or more of study weeks for both stool (71.8%, IQR 29.1%-92.2%) and nasal samples (71.0%, IQR 30.0%-90.5%). None of the compliant mothers withdrew from study and only 4 became inactive, but this inactivity occurred after the child reached 71 weeks of participation.

AGE and ARI incidence rates did not differ between the 245 children enrolled and the 194 children who maintained adequate compliance with the study protocol (Table 3). The incidence of AGE was 2.0 cases per child-year, with a median duration of 3 days. The incidence of ARI was 4.5 cases per child-year, with a median duration of 4 days. The compliant mothers reported a total of 627 AGE episodes and 1277 ARI episodes in their children over the course of follow-up. Of these, 160 (25.5%) AGE episodes were medically attended, while 486 (38.06%) ARI episodes were medically attended (difference in proportion between AGE and ARI episodes, *P*<.001); 3 out of 627 (0.5%) AGE cases were hospitalized; similarly, 7 out of 1277 ARI episodes were hospitalized (0.55%).

**Table 3:** Maternal report of AGE and ARI in study children.

Measure			All (N=245)		Compliant (N=194)
**AGE^a^ cases reported**					
	Number of cases	671		627	
	Child-weeks at risk^b^	17,473		16,020	
	Child-weeks symptomatic^c^, n/N (%)	710/18,183 (3.9)		657/16,677 (3.9)	
	Incidence: cases/100 child-weeks	3.8		3.9	
	Incidence: cases/child-year	2.0		2.0	
	Median days ill/case	3		3	
	Medically attended cases, n/N (%)	172/671 (25.6)		160/627 (25.5)	
	Hospitalized cases, n/N (%)	3/671 (0.4)		3/627 (0.5)	
**ARI^d^ cases reported**					
	Number of cases	1349		1277	
	Child-weeks at risk^b^	16,244		14,836	
	Child-weeks symptomatic^c^, n/N (%)	1939/18,183 (10.7)		1842/16,677 (11.0)	
	Incidence: cases/100 child-weeks	8.3		8.6	
	Incidence: cases/child-year	4.3		4.5	
	Median days ill/case	4		4	
	Medically attended cases, n/N (%)	521/1349 (38.6)		486/1277 (38.1)	
	Hospitalized cases, n/N (%)	8/1349 (0.6)		7/1277 (0.5)	

^a^AGE: acute gastroenteritis.

^b^The number of child-weeks at risk is calculated as the number of weeks of follow-up minus the number of weeks symptomatic

^c^Child-weeks symptomatic is calculated as the number of weeks when mothers reported symptoms divided by the total number of weeks that the child was under follow-up.

^d^ARI: acute respiratory infection.

## Discussion

The outcomes of the PREVAIL Cohort reported here are the generally high compliance rates that were obtained with our intensive study protocol, which was designed to ensure longitudinal characterization of repeated infections in the first 2 years of life. Of the 245 mother–child pairs enrolled, 194 (79.2%) were considered compliant with the sample collection and follow-up protocol. These mothers provided 384 child-years of follow-up, 13,048 stool samples, 13,546 weekly nasal samples, and 1050 scheduled blood samples from week 2 to year 2 of life. Median weekly completion of SMS text message surveys regarding the child’s AGE or ARI symptoms was 95.1% (IQR 76.5%-100%), though individual response varied. While mothers who were noncompliant differed in some measures of socioeconomic status, compliant mothers remained generally representative of the study population. Furthermore, we found an incidence of 2.0 AGE cases per child-year, and 4.5 ARI cases per child-year among study children over the first 2 years of life, as reported by mothers. Of these, 1 of 4 AGE cases and nearly 2 of 5 ARI cases were medically attended. Furthermore, 0.5% of AGE (3/627) cases and 0.55% of ARI (7/1277) cases were hospitalized. These findings indicate that our cohort methodology works in the US context, and that AGE and ARI in early childhood represent a significant public health problem.

The PREVAIL Cohort design is based upon the concept that infectious disease outcomes are the consequences of a cascade of diverse immunologic attributes and exposures, formed while in utero and shaped throughout life. Furthermore, the cohort is founded upon the premise that fully understanding the development of symptomatic infections also requires information on periods of asymptomatic infection. If public health interventions are devoted to prolonging this healthy period, then both symptomatic and asymptomatic longitudinal observations are indispensable.

We designed this study to untangle the web of influences that create disease and immunity among an intensively followed cohort of US children from their third trimester in utero to their second birthday. A comprehensive portfolio of clinical, epidemiologic, behavioral, and immunologic factors was studied during periods of good health, predisease, symptomatic and asymptomatic infections, and convalescence. Birth cohort studies are irreplaceable in providing an estimated protective efficacy that could potentially be achieved from well-designed vaccines through observing the pattern of immunity that develops against natural infections. Such cohort studies can also guide vaccine policy toward important target or vulnerable populations, optimal immunization ages, optimal vaccine dosage, and titer levels needed for long-lasting protection, and to better understand complementary interactions underpinning the creation of specific immunity at young ages.

Our study design was based upon the premise that understanding the true natural history of infection and immunity requires weekly sample and data collection, as exemplified by a Mexican birth cohort study that eminently provided the epidemiological framework required to develop both US-licensed rotavirus vaccines [34]. Through weekly data and stool samples and routine blood tested for rotavirus IgA and IgG from 200 Mexican children from birth to the 2nd birthday, that model cohort showed that first natural rotavirus infections are the most severe, but incremental and heterologous infections confer protection that typically leads to reduced severity—a concept now recognized as the operating principle for how the currently effective live-attenuated rotavirus vaccines work. In our cohort, we expect to describe antirotavirus immunologic responses by US mothers and infants during the postrotavirus vaccine era.

With the goals of enrollment and follow-up achieved, the PREVAIL Cohort successfully illustrates this proof of concept: It is possible to achieve an intensive infection and immunity birth cohort in the current-day US population, which involves keeping participants under weekly follow-up with high levels of compliance. Such efforts require significant infrastructure and technologic innovation to efficiently collect birth samples, weekly stool/nasal swab samples, weekly automated health status surveys, and systematically handle large-scale sample transport, management and analysis conducted by a collaborative network of public health disciplines.

This cohort anticipates applying these same methodological concepts to important norovirus vaccine development questions. Our observations of norovirus infections over time could improve the knowledge of how immunity against heterotypic strains is developed in young children. By comparing the symptoms associated with early norovirus infections with maternal antibody titers and markers of innate, genetic immunity (eg, polymorphism in *fucosyltransferase 2* [*FUT2*], which defines secretor status), this cohort could clarify antecedent contributions influencing infection risk and prolonging the asymptomatic period [6,35-37].

The PREVAIL Cohort aims to study pathogens contributing to serious ARI burden in the United States and worldwide, including RSV and influenza. Maternal and infant RSV vaccines are under investigation as potential preventive strategies, and a need exists to better understand vaccines designed to elicit antibodies to protect against infection in early life. Our data will characterize the infant immune response (neutralizing antibody titer and RSV-specific antibody profile) to primary and secondary RSV infections. We expect to better define the association of maternal RSV neutralizing antibodies (including the titer and RSV-specific antibody profile) with the risk of infant RSV infection, including correlations with transplacentally transferred RSV-specific maternal antibodies and breast milk immunomodulators. For influenza, we expect to longitudinally determine how initial and repeated natural influenza infections or influenza vaccinations (for both mother and offspring) shape immunity to future influenza exposures in the first 2 years of life. Ultimately, this information will be important to facilitate design of durable, broadly protective influenza vaccines.

Comparability of PREVAIL Cohort results to other published cohorts is not direct and should take into consideration wide variations in other cohort methodologies, low- and middle-income country settings, the ages of children studied, the degree of severity included in case definitions, and other factors. Sample collection and testing protocols also differ among cohorts, as described for norovirus cohorts by Cannon and others [38]. A limitation of many infection cohort studies worldwide has been that sample collection is undertaken on a monthly or less frequent basis, which could result in many missed asymptomatic infections. Weekly sample testing was the goal in the PREVAIL Cohort. While weekly sample collection was not perfect, it was achieved for the majority of child-weeks. We anticipate the combination of weekly sample testing and serology will optimize identification of infections, which we consider critical to deepen understanding of immune development.

There are other limitations in this study to consider. The AGE and ARI incidence rates reported here are based solely on maternal report. Medical records are currently being reviewed to obtain data on AGE and ARI events that resulted in health care system use that might not have been reported by the mother; these medical record data are not yet available for inclusion in this paper. Furthermore, laboratory analyses are underway to determine pathogen-specific outcomes and antibody response. The multiple outcomes measured in this cohort will be reported separately.

Lopman and Kang [27] argue that a cohort study of the natural history of infection is needed in the United States to guide maternal, infant, and childhood vaccine strategies, and this is applicable to all 4 of our focal pathogens (norovirus, rotavirus, RSV, and influenza). While incidence and severity may differ across regions and cohorts, many of the factors driving infectious disease burden at the youngest ages remain unknown, undermining our ability to estimate the impact of potential vaccines on disease burden. The National Academy of Science stated, “Without the comprehensive, longitudinal data provided by a [birth cohort study], it will be difficult to identify and make wise investments in policies that will promote health at the individual, community, and societal levels” [39]. The PREVAIL Cohort is expected to provide significant public health value by providing important information regarding the elicitation of immune protections against these childhood diseases, informing the development of new vaccines, improving upon vaccine strategies in this vulnerable infant population, and perhaps understanding the development of immunity for infectious diseases that are antigenically new to the human species.

## Appendix

Multimedia Appendix 1 PREVAIL AGE Survey.

Multimedia Appendix 2 PREVAIL ARI Survey.

Multimedia Appendix 3 PREVAIL Baseline Questionnaire.

Multimedia Appendix 4 PREVAIL Week 2 Questionnaire.

Multimedia Appendix 5 PREVAIL Month 24 Questionnaire.

Multimedia Appendix 6 PREVAIL Weekly Survey.

## Abbreviations

AGE: acute gastroenteritis
ARI: acute respiratory infection
CCHMC: Cincinnati Children’s Hospital Medical Center
CDC: Centers for Disease Control and Prevention
CRF: case report form
FUT2: fucosyltransferase 2
PREVAIL Cohort: Study acronym: Pediatric Respiratory and Enteric Virus Acquisition and Immunogenesis Longitudinal Cohort
REDCap: Research Electronic Data Capture (data management system)
RSV: respiratory syncytial virus
TCH: The Christ Hospital
UCMC: University of Cincinnati Medical Center
