# Supplementary material for: Pediatric Respiratory and Enteric Virus Acquisition and Immunogenesis in US Mothers and Children Aged 0-2: PREVAIL Cohort Study
Source: JMIR Res Protoc. 2021 Feb 12;10(2):e22222. doi: 10.2196/22222 (PMC7910118; doi:10.2196/22222)
Supplement: Multimedia Appendix 1 [file resprot_v10i2e22222_app1.pdf]

Txt AGE CRF

Please complete the survey below about [week_0_arm_1][nbcrf_inf_name]'s recent episode of vomitting and/or diarrhea.

If you have any questions or concerns, please contact a PREVAIL Research Coordinator.

About a week ago, you reported that [week_0_arm_1][nbcrf_inf_name] had vomiting and/or diarrhea. Please complete the following survey to tell us more about that illness.

What day did [week_0_arm_1][nbcrf_inf_name] become ill? __________________________________

DIARRHEA

Did [week_0_arm_1][nbcrf_inf_name] ever have diarrhea during this illness?

- ◯ No
- ◯ Yes
- ◯ Unsure/Unknown
- ◯ Refused/ No Response

How many days did [week_0_arm_1][nbcrf_inf_name] have diarrhea?

- ◯ Unsure/Unknown
- ◯ Refused/No Response
- ◯ 1
- ◯ 2
- ◯ 3
- ◯ 4
- ◯ 5
- ◯ 6
- ◯ 7
- ◯ 8
- ◯ 9
- ◯ 10
- ◯ 11
- ◯ 12
- ◯ 13
- ◯ 14
- ◯ 15
- ◯ 16
- ◯ 17
- ◯ 18
- ◯ 19
- ◯ 20
- ◯ 21
- ◯ 22
- ◯ 23
- ◯ 24
- ◯ 25
- ◯ 26
- ◯ 27
- ◯ 28
- ◯ 29
- ◯ 30
- ◯ 31

What was the greatest number of episodes in 24 hours?

(this is not necessarily the most recent 24 hour period)

- ◯ Unsure/Unknown
- ◯ Refused/No Response
- ◯ 1
- ◯ 2
- ◯ 3
- ◯ 4
- ◯ 5
- ◯ 6
- ◯ 7
- ◯ 8
- ◯ 9
- ◯ 10
- ◯ 11
- ◯ 12
- ◯ 13
- ◯ 14
- ◯ 15
- ◯ 16
- ◯ 17
- ◯ 18
- ◯ 19
- ◯ 20
- ◯ 21
- ◯ 22
- ◯ 23
- ◯ 24
- ◯ 25
- ◯ 26
- ◯ 27
- ◯ 28
- ◯ 29
- ◯ 30
- ◯ 31

VOMITING

Did [week_0_arm_1][nbcrf_inf_name] ever have vomiting during this illness?

- ◯ No
- ◯ Yes
- ◯ Unsure/Unknown
- ◯ Refused/ No Response

How many days did [week_0_arm_1][nbcrf_inf_name] have vomiting?	◯ Unsure/Unknown<br>◯ Refused/No Response<br>◯ 1<br>◯ 2<br>◯ 3<br>◯ 4<br>◯ 5<br>◯ 6<br>◯ 7<br>◯ 8<br>◯ 9<br>◯ 10<br>◯ 11<br>◯ 12<br>◯ 13<br>◯ 14<br>◯ 15<br>◯ 16<br>◯ 17<br>◯ 18<br>◯ 19<br>◯ 20<br>◯ 21<br>◯ 22<br>◯ 23<br>◯ 24<br>◯ 25<br>◯ 26<br>◯ 27<br>◯ 28<br>◯ 29<br>◯ 30<br>◯ 31

What was the greatest number of episodes in 24 hours?

(this is not necessarily the most recent 24 hour period)

- ◯ Unsure/Unknown
- ◯ Refused/No Response
- ◯ 1
- ◯ 2
- ◯ 3
- ◯ 4
- ◯ 5
- ◯ 6
- ◯ 7
- ◯ 8
- ◯ 9
- ◯ 10
- ◯ 11
- ◯ 12
- ◯ 13
- ◯ 14
- ◯ 15
- ◯ 16
- ◯ 17
- ◯ 18
- ◯ 19
- ◯ 20
- ◯ 21
- ◯ 22
- ◯ 23
- ◯ 24
- ◯ 25
- ◯ 26
- ◯ 27
- ◯ 28
- ◯ 29
- ◯ 30
- ◯ 31

FEVER

Did [week_0_arm_1][nbcrf_inf_name] ever have a fever during this illness?

- ◯ No
- ◯ Yes
- ◯ Unsure/Unknown
- ◯ Refused/No Response

How many days did [week_0_arm_1][nbcrf_inf_name] have a fever?	◯ Unsure/Unknown<br>◯ Refused/No Response<br>◯ 1<br>◯ 2<br>◯ 3<br>◯ 4<br>◯ 5<br>◯ 6<br>◯ 7<br>◯ 8<br>◯ 9<br>◯ 10<br>◯ 11<br>◯ 12<br>◯ 13<br>◯ 14<br>◯ 15<br>◯ 16<br>◯ 17<br>◯ 18<br>◯ 19<br>◯ 20<br>◯ 21<br>◯ 22<br>◯ 23<br>◯ 24<br>◯ 25<br>◯ 26<br>◯ 27<br>◯ 28<br>◯ 29<br>◯ 30<br>◯ 31
What was the highest temperature measured?	______________________________
Unit of measurement for temperature	◯ Fahrenheit<br>◯ Celsius
Temperature Method	◯ Rectal<br>◯ Armpit<br>◯ Mouth<br>◯ Ear<br>◯ Forehead<br>◯ Unsure/Unknown<br>◯ Other

Other Symptoms

What is the most severe sign or symptom [week_0_arm_1][nbcrf_inf_name] displayed during this illness?<br><br>(choose the most severe behavior, with seizure being the worst and normal being the least)	◯ Normal<br>◯ Less playful<br>◯ Fussy/Irritable<br>◯ Lethargic/Listless<br>◯ Seizure<br>◯ Unsure/Unknown<br>◯ Refused/No Response

Do you think that [week_0_arm_1][nbcrf_inf_name]'s eyes were normal or sunken, compared with before he/she was sick?	◯ Not sunken<br>◯ Sunken<br>◯ Unsure/Unknown<br>◯ Refused/No Response
Was your child uninterested in drinking, unable to drink or refused to feed?	◯ Took fluids normally<br>◯ Was uninterested in drinking and/or refused to feed<br>◯ Unsure/Unknown<br>◯ Refused/No Response
Skin Turgor<br><br>Please lightly pull on the child's skin to test dehydration, and tell me how long the skin stays pinched. Using thumb and forefinger, lightly pull the skin of the abdomen halfway between the child's die and belly button (with fold going in the direction of head to foot). Once the skin is pinched up, hold for a second and release by opening the finger and thumb	◯ Normal - skin retracts immediately<br>◯ Slowly - the fold is visible for less than 2 seconds<br>◯ Very slowly - the fold is visible for more than 2 seconds<br>◯ Unsure/Unknown<br>◯ Refused/No Response

Medical Visits for this Episode of Illness

How many times did [week_0_arm_1][nbcrf_inf_name] go to a doctor, clinic, emergency room or hospital during this episode of illness?	◯ 0<br>◯ 1<br>◯ 2<br>◯ 3<br>◯ 4 or more times
Medical Visit 1	
(1) Date of Visit	______________________________
(1) Date Description	◯ Exact<br>◯ Approximate<br>◯ Unknown<br>◯ No Response/Refused
(1) Location	◯ Doctor's office/Walk-in Clinic<br>◯ Emergency room<br>◯ Admitted to Hospital
Medical Visit 2	
(2) Date of Visit	______________________________
(2) Date Description	◯ Exact<br>◯ Approximate<br>◯ Unknown<br>◯ No Response/Refused
(2) Location	◯ Doctor's office/Walk-in clinic<br>◯ Emergency room<br>◯ Admitted to hospital
Medical Visit 3	

(3) Date of Visit	__________________________________
(3) Date Description	◯ Exact<br>◯ Approximate<br>◯ Unknown<br>◯ No Response/Refused
(3) Location	◯ Doctor's office/Walk-in Clinic<br>◯ Emergency room<br>◯ Admitted to Hospital

A member of the PREVAIL study staff will contact you to discuss [week_0_arm_1][nbcrf_inf_name]'s remaining medical visit/visits

If [week_0_arm_1][nbcrf_inf_name] has had 4 or more medical visits during this episode of illness, please enter the date, date description, and location here:	____

MEDICATIONS

Did [week_0_arm_1][nbcrf_inf_name] ever receive rehydration fluid by mouth at home, such as Pedialyte or Gatorade, or intravenous fluid from the hospital or ER?	◯ No<br>◯ Yes<br>◯ Unsure/Unknown<br>◯ Refused/No Response
Name the product or describe the fluid given:	__________________________________
Did [week_0_arm_1][nbcrf_inf_name] take any anti-diarrheal medications?	◯ No<br>◯ Yes<br>◯ Unsure/Unknown<br>◯ Refused/ No Response
Name of anti-diarrheal:	__________________________________
Did [week_0_arm_1][nbcrf_inf_name] receive any antibiotics for this illness?	◯ No<br>◯ Yes<br>◯ Unsure/Unknown<br>◯ Refused/No Response

What antibiotics were prescribed for this illness?

	Yes	No	Unsure/Unknown	Refused/No Response
Amoxicillin	◯	◯	◯	◯
Amoxicillin/Clavulanate (Augmentin)	◯	◯	◯	◯
Cefdinir (Omnicef)	◯	◯	◯	◯
Azithromycin (Zithromax)	◯	◯	◯	◯

Clindamycin	◯	◯	◯	◯
Ceftriaxone (Rocephin) (shot)	◯	◯	◯	◯
Levofloxacin (Levaquin)	◯	◯	◯	◯
Other	◯	◯	◯	◯
Unknown	◯	◯	◯	◯

Amoxicillin - Start Date: __________________________________

Amoxicillin - Date Description:
- ◯ Exact
- ◯ Approximate
- ◯ Unknown
- ◯ No Response/Refused

Amoxicillin/Clavulanate (Augmentin) - Start Date: __________________________________

Amoxicillin/Clavulanate (Augmentin) - Date Description:
- ◯ Exact
- ◯ Approximate
- ◯ Unknown
- ◯ No Response/Refused

Cefdinir (Omnicef) - Start Date __________________________________

Cefdinir (Omnicef) - Date Description
- ◯ Exact
- ◯ Approximate
- ◯ Unknown
- ◯ No Response/Refused

Azithromycin (Zithromax) - Start Date __________________________________

Azithromycin (Zithromax) - Date Description:
- ◯ Exact
- ◯ Approximate
- ◯ Unknown
- ◯ No Response/Refused

Clindamycin - Start Date: __________________________________

Clindamycin - Date Description:
- ◯ Exact
- ◯ Approximate
- ◯ Unknown
- ◯ No Response/Refused

Ceftriaxone (Rocephin) - Date shot administered: __________________________________

Ceftriaxone (Rocephin) - Date Description:
- ◯ Exact
- ◯ Approximate
- ◯ Unknown
- ◯ No Response/Refused

Levofloxacin (Levoquin) - Start Date: __________________________________

Levofloxacin (Levaquin) - Date Description:
- ◯ Exact
- ◯ Approximate
- ◯ Unknown
- ◯ No Response/Refused

If "Other" antibiotic, please specify the name here: ______________________

Other Antibiotic - Start Date: ______________________

Other Antibiotic - Date Description:
- ◯ Exact
- ◯ Approximate
- ◯ Unknown
- ◯ No Response/Refused

Unknown Antibiotic - Start Date: ______________________

Unknown Antibiotic - Date Description:
- ◯ Exact
- ◯ Approximate
- ◯ Unknown
- ◯ No Response/Refused

Will you be able to find out the name of the unknown antibiotic at a later time?
- ◯ No
- ◯ Yes

A member of the PREVAIL team will contact you soon to complete the unknown antibiotic information

INFANT SLEEP HABITS

On other questionnaires, we have asked you about how your baby sleeps. We are interested in knowing if infants' sleep habits change during illness.
During this illness, in which positions did you lay [week_0_arm_1][nbcrf_inf_name] down to sleep?

	Never	Rarely	Sometimes	Often	Always
On his or her side	◯	◯	◯	◯	◯
On his or her back	◯	◯	◯	◯	◯
On his or her stomach	◯	◯	◯	◯	◯

During this illness, how often has [week_0_arm_1][nbcrf_inf_name] slept alone in his or her own crib or bed?
- ◯ Never
- ◯ Rarely
- ◯ Sometimes
- ◯ Often
- ◯ Always

During this illness, who has [week_0_arm_1][nbcrf_inf_name] slept with then he or she is not sleeping alone?
- ◯ Me
- ◯ My Husband or Partner
- ◯ Someone else
- ◯ Not applicable, my child ALWAYS sleeps alone

If [week_0_arm_1][nbcrf_inf_name] sleeps with someone else, please tell us who: ______________________

During this illness, has your child EVER slept in any of the locations listed below?

Check all that apply

- ☐ In a crib, bassinet, or pack and play
- ☐ On a twin or larger mattress or bed
- ☐ On a couch, sofa or armchair
- ☐ In an infant car seat or swing
- ☐ In a sleeping sack or wearable blanket
- ☐ With a blanket
- ☐ With toys, cushions, or pillows, including nursing pillows
- ☐ With crib bumper pads (mesh or non-mesh)

AGE CASE DEFINITION

?An episode of AGE is defined as 3 or more loose or watery stools and/ or 1 or more vomiting episodes within 24 hours at any time in the previous week.
?An episode of AGE is considered ended when two or more asymptomatic days have occurred.

STAFF REPORT

Does the child appear to meet AGE case definition?

- ◯ Yes
- ◯ No

If no, why?

____

STUDY PHYSICIAN

Does the child appear to meet AGE case definition?

- ◯ Yes
- ◯ No

If no, why?

____
