# Supplementary material for: Pediatric Respiratory and Enteric Virus Acquisition and Immunogenesis in US Mothers and Children Aged 0-2: PREVAIL Cohort Study
Source: JMIR Res Protoc. 2021 Feb 12;10(2):e22222. doi: 10.2196/22222 (PMC7910118; doi:10.2196/22222)
Supplement: Multimedia Appendix 2 [file resprot_v10i2e22222_app2.pdf]

Txt ARI CRF

Please complete the survey below about [week_0_arm_1][nbcrf_inf_name]'s recent episode of cough and/or fever.

If you have any questions or concerns, please contact a PREVAIL Research Coordinator.

About a week ago, you reported that [week_0_arm_1][nbcrf_inf_name] had a cough and/or fever. Please complete the following survey to tell us more about that illness.

What day did [week_0_arm_1][nbcrf_inf_name] become ill?	__________________________________
Did [week_0_arm_1][nbcrf_inf_name] ever have a fever during this illness?	◯ No<br>◯ Yes<br>◯ Unsure/Unknown<br>◯ Refused/No Response
How many days did [week_0_arm_1][nbcrf_inf_name] have a fever?	◯ Unsure/Unknown<br>◯ Refused/No Response<br>◯ 1<br>◯ 2<br>◯ 3<br>◯ 4<br>◯ 5<br>◯ 6<br>◯ 7<br>◯ 8<br>◯ 9<br>◯ 10<br>◯ 11<br>◯ 12<br>◯ 13<br>◯ 14<br>◯ 15<br>◯ 16<br>◯ 17<br>◯ 18<br>◯ 19<br>◯ 20<br>◯ 21<br>◯ 22<br>◯ 23<br>◯ 24<br>◯ 25<br>◯ 26<br>◯ 27<br>◯ 28<br>◯ 29<br>◯ 30<br>◯ 31
What was the highest temperature measured?	__________________________________
Unit of measurement for temperature	◯ Fahrenheit<br>◯ Celsius

Temperature Method	◯ Rectal<br>◯ Armpit<br>◯ Mouth<br>◯ Ear<br>◯ Forehead<br>◯ Unsure/Unknown<br>◯ Other

SYMPTOMS

Did [week_0_arm_1][nbcrf_inf_name] have a cough during this illness?	◯ No<br>◯ Yes<br>◯ Unsure/Unknown<br>◯ Refused/ No Response
How many days did [week_0_arm_1][nbcrf_inf_name] have a cough?	◯ Unsure/Unknown<br>◯ Refused/No Response<br>◯ 1<br>◯ 2<br>◯ 3<br>◯ 4<br>◯ 5<br>◯ 6<br>◯ 7<br>◯ 8<br>◯ 9<br>◯ 10<br>◯ 11<br>◯ 12<br>◯ 13<br>◯ 14<br>◯ 15<br>◯ 16<br>◯ 17<br>◯ 18<br>◯ 19<br>◯ 20<br>◯ 21<br>◯ 22<br>◯ 23<br>◯ 24<br>◯ 25<br>◯ 26<br>◯ 27<br>◯ 28<br>◯ 29<br>◯ 30<br>◯ 31
Did [week_0_arm_1][nbcrf_inf_name] cough up mucous or phlegm?	◯ No<br>◯ Yes<br>◯ Unknown/ Unsure<br>◯ Refused/ No Response
Did [week_0_arm_1][nbcrf_inf_name] cough up blood?	◯ No<br>◯ Yes<br>◯ Unsure/ Unknown<br>◯ Refused/ No Response

Did [week_0_arm_1][nbcrf_inf_name] ever vomit right after coughing?

- ◯ No
- ◯ Yes
- ◯ Unsure/Unknown
- ◯ Refused/ No Response

Did [week_0_arm_1][nbcrf_inf_name] have any of the following symptoms during this illness?

	Yes	No	Unsure/Unknown	Refused/No Response
Earache	◯	◯	◯	◯
Nasal congestion or runny nose	◯	◯	◯	◯
Sore throat	◯	◯	◯	◯
Wheezing	◯	◯	◯	◯
Rapid or shallow breathing/shortness of breath (dyspnea)	◯	◯	◯	◯
Chills	◯	◯	◯	◯
Fatigue/weakness	◯	◯	◯	◯
Muscle aches (myalgias)	◯	◯	◯	◯
Headache	◯	◯	◯	◯
Red/pink eye(s) (conjunctivitis)	◯	◯	◯	◯
Loss of appetite/refusal to eat	◯	◯	◯	◯
Abdominal pain	◯	◯	◯	◯
Vomiting (not associated with coughing)	◯	◯	◯	◯
Diarrhea	◯	◯	◯	◯
Listless/lethargic	◯	◯	◯	◯
Confusion	◯	◯	◯	◯
Irritability/fussiness	◯	◯	◯	◯
Seizures	◯	◯	◯	◯
Skin rash	◯	◯	◯	◯
Other	◯	◯	◯	◯

If "other" symptoms, please describe:

____

Medical Visits

How many times did [week_0_arm_1][nbcrf_inf_name] go to a doctor, clinic, emergency room or hospital during this episode of illness?

- ◯ 0
- ◯ 1
- ◯ 2
- ◯ 3
- ◯ 4 or more times

Medical Visit 1

(1) Date of Visit

____

(1) Date Description	◯ Exact<br>◯ Approximate<br>◯ Unknown<br>◯ No Response/Refused
(1) Location	◯ Doctor's office/Clinic<br>◯ Emergency room<br>◯ Admitted to hospital
Medical Visit 2	
(2) Date of Visit	______________________
(2) Date Description	◯ Exact<br>◯ Approximate<br>◯ Unknown<br>◯ No Response/Refused
(2) Location	◯ Doctor's office/Clinic<br>◯ Emergency room<br>◯ Admitted to hospital
Medical Visit 3	
(3) Date of Visit	______________________
(3) Date Description	◯ Exact<br>◯ Approximate<br>◯ Unknown<br>◯ No Response/Refused
(3) Location	◯ Doctor's office/Clinic<br>◯ Emergency room<br>◯ Admitted to hospital
A member of the PREVAIL study staff will contact you to discuss [week_0_arm_1][nbcrf_inf_name]'s remaining medical visit/visits	
If [week_0_arm_1][nbcrf_inf_name] has had 4 or more medical visits during this episode of illness, please enter the date, date description, and location here:	___________________________

MEDICATIONS

Did [week_0_arm_1][nbcrf_inf_name] receive any antibiotics for this illness?	◯ No<br>◯ Yes<br>◯ Unsure/Unknown<br>◯ Refused/No Response

What antibiotics were prescribed for this illness?

	Yes	No	Unsure/Unknown	Refused/No Response
Amoxicillin	◯	◯	◯	◯
Amoxicillin/Clavulanate (Augmentin)	◯	◯	◯	◯
Cefdinir (Omnicef)	◯	◯	◯	◯
Azithromycin (Zithromax)	◯	◯	◯	◯
Clindamycin	◯	◯	◯	◯
Ceftriaxone (Rocephin) (shot)	◯	◯	◯	◯
Levofloxacin (Levaquin)	◯	◯	◯	◯
Other	◯	◯	◯	◯
Unknown	◯	◯	◯	◯

Amoxicillin - Start Date: ______________________

Amoxicillin - Date Description:
◯ Exact
◯ Approximate
◯ Unknown
◯ No Response/Refused

Amoxicillin/Clavulanate (Augmentin) - Start Date: ______________________

Amoxicillin/Clavulanate (Augmentin) - Date Description:
◯ Exact
◯ Approximate
◯ Unknown
◯ No Response/Refused

Cefdinir (Omnicef) - Start Date ______________________

Cefdinir (Omnicef) - Date Description
◯ Exact
◯ Approximate
◯ Unknown
◯ No Response/Refused

Azithromycin (Zithromax) - Start Date ______________________

Azithromycin (Zithromax) - Date Description:
◯ Exact
◯ Approximate
◯ Unknown
◯ No Response/Refused

Clindamycin - Start Date: ______________________

Clindamycin - Date Description:
◯ Exact
◯ Approximate
◯ Unknown
◯ No Response/Refused

Ceftriaxone (Rocephin) - Date shot administered: __________________________________

Ceftriaxone (Rocephin) - Date Description:
- ◯ Exact
- ◯ Approximate
- ◯ Unknown
- ◯ No Response/Refused

Levofloxacin (Levoquin) - Start Date: __________________________________

Levofloxacin (Levaquin) - Date Description:
- ◯ Exact
- ◯ Approximate
- ◯ Unknown
- ◯ No Response/Refused

If "Other" antibiotic, please specify the name here: __________________________________

Other Antibiotic - Start Date: __________________________________

Other Antibiotic - Date Description:
- ◯ Exact
- ◯ Approximate
- ◯ Unknown
- ◯ No Response/Refused

Unknown Antibiotic - Start Date: __________________________________

Unknown Antibiotic - Date Description:
- ◯ Exact
- ◯ Approximate
- ◯ Unknown
- ◯ No Response/Refused

Will you be able to find out the name of the unknown antibiotic at a later time?
- ◯ No
- ◯ Yes

A member of the PREVAIL team will contact you soon to complete the unknown antibiotic information

Did [week_0_arm_1][nbcrf_inf_name] receive any influenza-specific antiviral medications for this illness?
- ◯ No
- ◯ Yes
- ◯ Unsure/Unknown
- ◯ Refused/No Response

What antivirals were prescribed for this illness?

	Yes	No	Unsure/Unknown	Refused/No Response
Oseltamivir (Generic or Tamiflu)	◯	◯	◯	◯
Other	◯	◯	◯	◯

Unknown	◯	◯	◯	◯

Oseltamivir (generic or Tamiflu), oral - Start Date: __________________

Oseltamivir (generic or Tamiflu), oral - Date Description:
- ◯ Exact
- ◯ Approximate
- ◯ Unknown
- ◯ No Response/Refused

If "Other" antiviral, please specify the name here: __________________

Other Antiviral - Start Date: __________________

Other Antiviral - Date Description:
- ◯ Exact
- ◯ Approximate
- ◯ Unknown
- ◯ No Response/Refused

Unknown Antiviral - Start Date: __________________

Unknown Antiviral - Date Description:
- ◯ Exact
- ◯ Approximate
- ◯ Unknown
- ◯ No Response/Refused

Will you be able to find out the name of the unknown antiviral medication at a later time?
- ◯ No
- ◯ Yes

A member of the PREVAIL study staff will contact you within a week to discuss [week_0_arm_1][nbcrf_inf_name]'s antiviral medication

INFANT SLEEP HABITS

On other questionnaires, we have asked you about how your baby sleeps. We are interested in knowing if infants' sleep habits change during illness.
During this illness, in which positions did you lay [week_0_arm_1][nbcrf_inf_name] down to sleep?

	Never	Rarely	Sometimes	Often	Always
On his or her side	◯	◯	◯	◯	◯
On his or her back	◯	◯	◯	◯	◯
On his or her stomach	◯	◯	◯	◯	◯

During this illness, how often has [week_0_arm_1][nbcrf_inf_name] slept alone in his or her own crib or bed?
- ◯ Never
- ◯ Rarely
- ◯ Sometimes
- ◯ Often
- ◯ Always

During this illness, who has [week_0_arm_1][nbcrf_inf_name] slept with when he or she is not sleeping alone?

- ☐ Me
- ☐ My Husband or Partner
- ☐ Someone else
- ☐ Not applicable, my child ALWAYS sleeps alone

If [week_0_arm_1][nbcrf_inf_name] sleeps with someone else, please tell us who:

____

During this illness, has your child EVER slept in any of the locations listed below?

Check all that apply

- ☐ In a crib, bassinet, or pack and play
- ☐ On a twin or larger mattress or bed
- ☐ On a couch, sofa or armchair
- ☐ In an infant car seat or swing
- ☐ In a sleeping sack or wearable blanket
- ☐ With a blanket
- ☐ With toys, cushions, or pillows, including nursing pillows
- ☐ With crib bumper pads (mesh or non-mesh)

ARI CASE DEFINITION

An ARI episode is defined as the presence of cough OR fever (temperature 38.0 C/100.4 F, rectal/typannic; 37.0 C/98.6 F, axillary/temporal or 37.3 C/99.5 F oral) at any time in the previous week. An episode of ARI will be considered ended when two or more asymptomatic days have occurred.

STUDY STAFF

Does the child appear to meet ARI case definition?

- ◯ Yes
- ◯ No

If no, why?

____

STUDY PHYSICIAN

Does the child appear to meet ARI case definition?

- ◯ Yes
- ◯ No

If no, why?

____
