# Supplementary material for: Pediatric Respiratory and Enteric Virus Acquisition and Immunogenesis in US Mothers and Children Aged 0-2: PREVAIL Cohort Study
Source: JMIR Res Protoc. 2021 Feb 12;10(2):e22222. doi: 10.2196/22222 (PMC7910118; doi:10.2196/22222)
Supplement: Multimedia Appendix 3 [file resprot_v10i2e22222_app3.pdf]

Baseline Questionnaire

Record ID ________________________________

Subject ID ([consent_arm_1][subjectid]) ________________________________

Date ________________________________

CRC Initials ________________________________

Address ________________________________

Latitude ________________________________

Longitude ________________________________

Census Tract ID number ________________________________

Deprivation Index Score ________________________________

Demographic Information

What is your date of birth? ________________________________
(mother's date of birth, not her infant's)

Do you currently have health insurance?
- ◯ No
- ◯ Yes
- ◯ Unsure/Unknown
- ◯ Refused/No Response

What kind of insurance do you have?
- ◯ Public
- ◯ Private
- ◯ Both
- ◯ Unsure if public or private/Unknown
- ◯ Refused/No Response

Are you of Hispanic, Latina, or Spanish origin?
- ◯ No
- ◯ Yes
- ◯ Unsure/Unknown
- ◯ Refused/No Response

Are you: (check all that apply)	☐ White<br>☐ Black, African American<br>☐ American Indian/ Alaskan Native<br>☐ Asian<br>☐ Native Hawaiian/ Pacific Islander<br>☐ None<br>☐ Unsure/ Unknown<br>☐ Refused/ No Response
What is your marital status?	○ Married<br>○ Divorced<br>○ Widowed<br>○ Separated<br>○ Never Married<br>○ A Member of an Unmarried Couple<br>○ Unsure/ Unknown<br>○ Refused/ No Response

Social History

Including yourself, how many people live in your household?	○ 1<br>○ 2<br>○ 3<br>○ 4<br>○ 5<br>○ 6<br>○ 7<br>○ More than 7 people<br>○ Unsure/Unknown<br>○ Refused/No Response
Please list all household members:<br><br>(Including the study participant/mother)	
Household member 1	

(1) Household member's relation to child	◯ Study Infant<br>◯ Study Mother<br>◯ Father<br>◯ Sister<br>◯ Brother<br>◯ Aunt<br>◯ Uncle<br>◯ Cousin<br>◯ Maternal Grandmother<br>◯ Maternal Grandfather<br>◯ Paternal Grandmother<br>◯ Paternal Grandfather<br>◯ Maternal Great Grandmother<br>◯ Maternal Great Grandfather<br>◯ Paternal Great Grandmother<br>◯ Paternal Great Grandfather<br>◯ Family Friend<br>◯ Significant Other (study mother's significant other that is not the infant's father)<br>◯ Other<br>◯ Grandmother<br>◯ Grandfather<br>◯ Great Grandmother<br>◯ Great Grandfather
(1) If relation is "other", please describe:	______________________________
(1) Age	______________________________<br>(Age in years)
(1) Sex	◯ Male<br>◯ Female

Household member 2

(2) Household member's relation to child	◯ Study Infant<br>◯ Study Mother<br>◯ Father<br>◯ Sister<br>◯ Brother<br>◯ Aunt<br>◯ Uncle<br>◯ Cousin<br>◯ Maternal Grandmother<br>◯ Maternal Grandfather<br>◯ Paternal Grandmother<br>◯ Paternal Grandfather<br>◯ Maternal Great Grandmother<br>◯ Maternal Great Grandfather<br>◯ Paternal Great Grandmother<br>◯ Paternal Great Grandfather<br>◯ Family Friend<br>◯ Significant Other (study mother's significant other that is not the infant's father)<br>◯ Other<br>◯ Grandmother<br>◯ Grandfather<br>◯ Great Grandmother<br>◯ Great Grandfather
(2) If relation is "other", please describe:	______________________
(2) Age	______________________<br>(Age in years)
(2) Sex	◯ Male<br>◯ Female

Household member 3

(3) Household member's relation to child	◯ Study Infant<br>◯ Study Mother<br>◯ Father<br>◯ Sister<br>◯ Brother<br>◯ Aunt<br>◯ Uncle<br>◯ Cousin<br>◯ Maternal Grandmother<br>◯ Maternal Grandfather<br>◯ Paternal Grandmother<br>◯ Paternal Grandfather<br>◯ Maternal Great Grandmother<br>◯ Maternal Great Grandfather<br>◯ Paternal Great Grandmother<br>◯ Paternal Great Grandfather<br>◯ Family Friend<br>◯ Significant Other (study mother's significant other that is not the infant's father)<br>◯ Other<br>◯ Grandmother<br>◯ Grandfather<br>◯ Great Grandmother<br>◯ Great Grandfather
(3) If relation is "other", please describe:	____________________________
(3) Age	____________________________<br>(Age in years)
(3) Sex	◯ Male<br>◯ Female

Household member 4

(4) Household member's relation to child	◯ Study Infant<br>◯ Study Mother<br>◯ Father<br>◯ Sister<br>◯ Brother<br>◯ Aunt<br>◯ Uncle<br>◯ Cousin<br>◯ Maternal Grandmother<br>◯ Maternal Grandfather<br>◯ Paternal Grandmother<br>◯ Paternal Grandfather<br>◯ Maternal Great Grandmother<br>◯ Maternal Great Grandfather<br>◯ Paternal Great Grandmother<br>◯ Paternal Great Grandfather<br>◯ Family Friend<br>◯ Significant Other (study mother's significant other that is not the infant's father)<br>◯ Other<br>◯ Grandmother<br>◯ Grandfather<br>◯ Great Grandmother<br>◯ Great Grandfather
(4) If relation is "other", please describe:	______________________
(4) Age	______________________<br>(Age in years)
(4) Sex	◯ Male<br>◯ Female
Household member 5	

(5) Household member's relation to child

◯ Study Infant
◯ Study Mother
◯ Father
◯ Sister
◯ Brother
◯ Aunt
◯ Uncle
◯ Cousin
◯ Maternal Grandmother
◯ Maternal Grandfather
◯ Paternal Grandmother
◯ Paternal Grandfather
◯ Maternal Great Grandmother
◯ Maternal Great Grandfather
◯ Paternal Great Grandmother
◯ Paternal Great Grandfather
◯ Family Friend
◯ Significant Other (study mother's significant other that is not the infant's father)
◯ Other
◯ Grandmother
◯ Grandfather
◯ Great Grandmother
◯ Great Grandfather

(5) If relation is "other", please describe:

____

(5) Age

____
(Age in years)

(5) Sex

◯ Male
◯ Female

Household member 6

(6) Household member's relation to child	◯ Study Infant<br>◯ Study Mother<br>◯ Father<br>◯ Sister<br>◯ Brother<br>◯ Aunt<br>◯ Uncle<br>◯ Cousin<br>◯ Maternal Grandmother<br>◯ Maternal Grandfather<br>◯ Paternal Grandmother<br>◯ Paternal Grandfather<br>◯ Maternal Great Grandmother<br>◯ Maternal Great Grandfather<br>◯ Paternal Great Grandmother<br>◯ Paternal Great Grandfather<br>◯ Family Friend<br>◯ Significant Other (study mother's significant other that is not the infant's father)<br>◯ Other<br>◯ Grandmother<br>◯ Grandfather<br>◯ Great Grandmother<br>◯ Great Grandfather
(6) If relation is "other", please describe:	__________________________________
(6) Age	__________________________________<br>(Age in years)
(6) Sex	◯ Male<br>◯ Female

Household member 7

(7) Household member's relation to child	◯ Study Infant<br>◯ Study Mother<br>◯ Father<br>◯ Sister<br>◯ Brother<br>◯ Aunt<br>◯ Uncle<br>◯ Cousin<br>◯ Maternal Grandmother<br>◯ Maternal Grandfather<br>◯ Paternal Grandmother<br>◯ Paternal Grandfather<br>◯ Maternal Great Grandmother<br>◯ Maternal Great Grandfather<br>◯ Paternal Great Grandmother<br>◯ Paternal Great Grandfather<br>◯ Family Friend<br>◯ Significant Other (study mother's significant other that is not the infant's father)<br>◯ Other<br>◯ Grandmother<br>◯ Grandfather<br>◯ Great Grandmother<br>◯ Great Grandfather
(7) If relation is "other", please describe:	______________________________
(7) Age	______________________________<br>(Age in years)
(7) Sex	◯ Male<br>◯ Female
If there are more than 7 people living in your household, please describe their relationship to the child, their age in years and their sex here:	______________________________

(8) Household member's relation to child

- ◯ Study Infant
- ◯ Study Mother
- ◯ Father
- ◯ Sister
- ◯ Brother
- ◯ Aunt
- ◯ Uncle
- ◯ Cousin
- ◯ Maternal Grandmother
- ◯ Maternal Grandfather
- ◯ Paternal Grandmother
- ◯ Paternal Grandfather
- ◯ Maternal Great Grandmother
- ◯ Maternal Great Grandfather
- ◯ Paternal Great Grandmother
- ◯ Paternal Great Grandfather
- ◯ Family Friend
- ◯ Significant Other (study mother's significant other that is not the infant's father)
- ◯ Other
- ◯ Grandmother
- ◯ Grandfather
- ◯ Great Grandmother
- ◯ Great Grandfather

(9) Household member's relation to child

- ◯ Study Infant
- ◯ Study Mother
- ◯ Father
- ◯ Sister
- ◯ Brother
- ◯ Aunt
- ◯ Uncle
- ◯ Cousin
- ◯ Maternal Grandmother
- ◯ Maternal Grandfather
- ◯ Paternal Grandmother
- ◯ Paternal Grandfather
- ◯ Maternal Great Grandmother
- ◯ Maternal Great Grandfather
- ◯ Paternal Great Grandmother
- ◯ Paternal Great Grandfather
- ◯ Family Friend
- ◯ Significant Other (study mother's significant other that is not the infant's father)
- ◯ Other
- ◯ Grandmother
- ◯ Grandfather
- ◯ Great Grandmother
- ◯ Great Grandfather

(10) Household member's relation to child

- ◯ Study Infant
- ◯ Study Mother
- ◯ Father
- ◯ Sister
- ◯ Brother
- ◯ Aunt
- ◯ Uncle
- ◯ Cousin
- ◯ Maternal Grandmother
- ◯ Maternal Grandfather
- ◯ Paternal Grandmother
- ◯ Paternal Grandfather
- ◯ Maternal Great Grandmother
- ◯ Maternal Great Grandfather
- ◯ Paternal Great Grandmother
- ◯ Paternal Great Grandfather
- ◯ Family Friend
- ◯ Significant Other (study mother's significant other that is not the infant's father)
- ◯ Other
- ◯ Grandmother
- ◯ Grandfather
- ◯ Great Grandmother
- ◯ Great Grandfather

(11) Household member's relation to child

- ◯ Study Infant
- ◯ Study Mother
- ◯ Father
- ◯ Sister
- ◯ Brother
- ◯ Aunt
- ◯ Uncle
- ◯ Cousin
- ◯ Maternal Grandmother
- ◯ Maternal Grandfather
- ◯ Paternal Grandmother
- ◯ Paternal Grandfather
- ◯ Maternal Great Grandmother
- ◯ Maternal Great Grandfather
- ◯ Paternal Great Grandmother
- ◯ Paternal Great Grandfather
- ◯ Family Friend
- ◯ Significant Other (study mother's significant other that is not the infant's father)
- ◯ Other
- ◯ Grandmother
- ◯ Grandfather
- ◯ Great Grandmother
- ◯ Great Grandfather

Total number of people that live in the household?

- ◯ 0
- ◯ 1
- ◯ 2
- ◯ 3
- ◯ 4
- ◯ 5
- ◯ 6
- ◯ 7
- ◯ 8
- ◯ 9
- ◯ 10
- ◯ 11
- ◯ 12
- ◯ 13
- ◯ 14
- ◯ 15
- ◯ 16
- ◯ 17
- ◯ 18
- ◯ 19
- ◯ 20
- ◯ 21
- ◯ 22
- ◯ 23
- ◯ 24
- ◯ 25
- ◯ 26
- ◯ 27
- ◯ 28
- ◯ 29
- ◯ 30
- ◯ 88 Unknown

Total number of people under 5 years of age that live in the household?

- ◯ 0
- ◯ 1
- ◯ 2
- ◯ 3
- ◯ 4
- ◯ 5
- ◯ 6
- ◯ 7
- ◯ 8
- ◯ 9
- ◯ 10
- ◯ 11
- ◯ 12
- ◯ 13
- ◯ 14
- ◯ 15
- ◯ 16
- ◯ 17
- ◯ 18
- ◯ 19
- ◯ 20
- ◯ 21
- ◯ 22
- ◯ 23
- ◯ 24
- ◯ 25
- ◯ 26
- ◯ 27
- ◯ 28
- ◯ 29
- ◯ 30
- ◯ 88 Unknown

Total number of people between 5-17 years that live in the household?

○ 0
○ 1
○ 2
○ 3
○ 4
○ 5
○ 6
○ 7
○ 8
○ 9
○ 10
○ 11
○ 12
○ 13
○ 14
○ 15
○ 16
○ 17
○ 18
○ 19
○ 20
○ 21
○ 22
○ 23
○ 24
○ 25
○ 26
○ 27
○ 28
○ 29
○ 30
○ 88 Unknown

Including the study mother, total number of people between 18-64 that live in the household?

◯ 1
◯ 2
◯ 3
◯ 4
◯ 5
◯ 6
◯ 7
◯ 8
◯ 9
◯ 10
◯ 11
◯ 12
◯ 13
◯ 14
◯ 15
◯ 16
◯ 17
◯ 18
◯ 19
◯ 20
◯ 21
◯ 22
◯ 23
◯ 24
◯ 25
◯ 26
◯ 27
◯ 28
◯ 29
◯ 30
◯ 88 Unknown

Total number of people 65 years and older that live in the household?	◯ 0<br>◯ 1<br>◯ 2<br>◯ 3<br>◯ 4<br>◯ 5<br>◯ 6<br>◯ 7<br>◯ 8<br>◯ 9<br>◯ 10<br>◯ 11<br>◯ 12<br>◯ 13<br>◯ 14<br>◯ 15<br>◯ 16<br>◯ 17<br>◯ 18<br>◯ 19<br>◯ 20<br>◯ 21<br>◯ 22<br>◯ 23<br>◯ 24<br>◯ 25<br>◯ 26<br>◯ 27<br>◯ 28<br>◯ 29<br>◯ 30<br>◯ 88 Unknown
What is the highest degree or diploma you have completed?	◯ None<br>◯ GED<br>◯ High School Diploma<br>◯ 2-Year College Degree (Associate's or Technical Degree)<br>◯ 4-Year College Degree (Bachelor's Degree)<br>◯ Graduate Degree (Master's, Doctorate, Medical, etc.)<br>◯ Unsure/ Unknown<br>◯ Refused/ No Response
Please choose the range that best represents your household income last year (before taxes):	◯ Less than or equal $25,000<br>◯ Between $25 and 50 thousand ($25,001-$50,000)<br>◯ Between $50 and 75 thousand ($50,001-$75,000)<br>◯ Between $75 and 100 thousand ($75,001-$100,000)<br>◯ Between $100 and 125 thousand ($100,001-$125,000)<br>◯ Between $125 and $150 thousand ($125,001-$150,000)<br>◯ Over $150,000<br>◯ Unsure, Unknown<br>◯ Refused/ No Response

Recent Health History

Are you currently or were you ever enrolled in the ADORE Trial?	◯ Yes<br>◯ No
What was your weight at the time you became pregnant with this child?	____________________<br>(lbs.)
During this pregnancy have you had symptoms of a respiratory illness such as a cough, earache, nasal congestion, running nose, shortness of breath, rapid or shallow breathing, sore throat, vomiting after cough or wheezing?	◯ No<br>◯ Yes<br>◯ Unsure/ Unknown<br>◯ Refused/ No Response
If yes, how many times during this pregnancy did you experience one or more of those symptoms listed?	◯ 1<br>◯ 2<br>◯ 3<br>◯ 4 or more times<br>◯ Unsure/ Unknown<br>◯ Refused/ No Response
(1) Date you began to feel sick with an illness	____________________
(1) Date Description:	◯ Exact<br>◯ Approximate<br>◯ Unknown<br>◯ No Response/Refused
(2) Second date you began to feel sick with an illness	____________________
(2) Date Description:	◯ Exact<br>◯ Approximate<br>◯ Unknown<br>◯ No Response/Refused
(3) Third date you began to feel sick with an illness	____________________
(3) Date Description:	◯ Exact<br>◯ Approximate<br>◯ Unknown<br>◯ No Response/Refused
If you felt sick 4 or more times, please list the dates here and provide the date description:	_________________________
If yes, did any of these illnesses result in a doctor's visit or hospital stay?	◯ No<br>◯ Yes, doctor's visit<br>◯ Yes, hospitalization<br>◯ Unsure/ Unknown<br>◯ Refused/ No Response

Did you take oseltamivir (Tamiflu or generic version) or a similar specific prescription antiviral medication as treatment for your illness?	◯ No<br>◯ Yes<br>◯ Unsure/ Unknown<br>◯ Refused/ No Response
If yes, what medication did you take?	☐ Oseltamivir (generic version or Tamiflu)<br>☐ Zanamivir (Relenza)<br>☐ Unsure/ Unknown<br>☐ Refused/ No Response
During this pregnancy have you had any diarrhea or vomiting requiring medical attention?	◯ No<br>◯ Yes<br>◯ Unsure/ Unknown<br>◯ Refused/ No Response
If yes, what was the date of the most recent symptoms?	__________________________
Date Description:	◯ Exact<br>◯ Approximate<br>◯ Unknown<br>◯ No Response/Refused
During this pregnancy have you visited an emergency room or been hospitalized?	◯ No<br>◯ Yes<br>◯ Unsure/ Unknown<br>◯ Refused/ No Response
What was the date of the most recent event?	__________________________
Date Description:	◯ Exact<br>◯ Approximate<br>◯ Unknown<br>◯ No Response/Refused
What was the diagnosis?	◯ Contractions<br>◯ Leaking Fluids<br>◯ Vaginal Bleeding<br>◯ High Blood Pressure<br>◯ GI Disturbance (Nausea, Vomiting, Diarrhea or Dehydration)<br>◯ Abdominal Pain<br>◯ Other<br>◯ Unknown/ Unsure<br>◯ Refused/ No Response
If the diagnosis was "other", please describe:	__________________________
During this pregnancy have you taken any prenatal vitamins, probiotics, DHA (docosahexaenoic acid), or other supplements?	◯ No<br>◯ Yes<br>◯ Unsure/ Unknown<br>◯ Refused/ No Response

If yes, what have you taken during this pregnancy?	☐ Prenatal Vitamins<br>☐ Probiotics<br>☐ DHA (docosahexaenoic acid)<br>☐ Other Supplements
Have you received any antibiotics by mouth, IV or injection during this pregnancy?	◯ No<br>◯ Yes<br>◯ Unknown/ Unsure<br>◯ Refused/ No Response
If yes, how many times during your pregnancy did you receive either antibiotics by mouth, IV or injection?	◯ 1<br>◯ 2<br>◯ 3<br>◯ 4 or more times<br>◯ Unsure/ Unknown<br>◯ Refused/ No Response
(1) Date first antibiotic was prescribed	______________________
(1) Date Description:	◯ Exact<br>◯ Approximate<br>◯ Unknown<br>◯ No Response/Refused
(2) Date second antibiotic was prescribed	______________________
(2) Date Description:	◯ Exact<br>◯ Approximate<br>◯ Unknown<br>◯ No Response/Refused
(3) Date third antibiotic was prescribed	______________________
(3) Date Description:	◯ Exact<br>◯ Approximate<br>◯ Unknown<br>◯ No Response/Refused
If you were prescribed antibiotics four or more times, please list the dates here and provide the date description:	___________________________
If yes, what were the antibiotics prescribed for?<br><br>Check all that apply	☐ Sore Throat<br>☐ Respiratory Infection<br>☐ Ear Infection<br>☐ Diarrhea and/or Vomiting<br>☐ Fever<br>☐ Skin Infection<br>☐ Urinary Tract Infection<br>☐ Sexually Transmitted Disease<br>☐ Other<br>☐ Unsure/ Unknown<br>☐ Refused/ No Response
If other, please specify:	______________________

If STI, please specify:	◯ Chlamydia<br>◯ Gonorrhea<br>◯ Syphilis<br>◯ Trichomiasis<br>◯ Unknown/ Unsure<br>◯ No Response/ Refused
During this pregnancy, did you travel or live outside the United States? (Including Mexico, Canada and the Caribbean)	◯ No<br>◯ Yes<br>◯ Unsure/ Unknown<br>◯ Refused/ No Response
If yes, how many countries did you travel to during this pregnancy?	◯ 1<br>◯ 2<br>◯ 3<br>◯ 4 or more countries<br>◯ Unsure/ Unknown<br>◯ Refused/ No Response
(1) Country 1	
(1) What is the name of the first country?	______________________________
(1) What date did you travel to this country?	______________________________
(1) Date Description:	◯ Exact<br>◯ Approximate<br>◯ Unknown<br>◯ No Response/Refused
(2) Country 2	
(2) What is the name of the second country?	______________________________
(2) What date did you travel to this country?	______________________________
(2) Date Description:	◯ Exact<br>◯ Approximate<br>◯ Unknown<br>◯ No Response/Refused
(3) Country 3	
(3) What is the name of the third country?	______________________________
(3) What date did you travel to this country?	______________________________
(3) Date Description:	◯ Exact<br>◯ Approximate<br>◯ Unknown<br>◯ No Response/Refused

If you traveled to four or more countries during your pregnancy, please specify the remaining countries, dates of travel, and date descriptions:	______________________________
Have you smoked any cigarettes before or during this pregnancy?	◯ No<br>◯ Yes<br>◯ Unsure/ Unknown<br>◯ Refused/ No Response
If yes, which time periods during your pregnancy did you smoke?	☐ Three months before pregnancy<br>☐ First three months of pregnancy<br>☐ Second three months of pregnancy<br>☐ Third trimester of pregnancy
During the three months before pregnancy, what's the average number of cigarettes you smoked per day?	______________________
During the first three months of pregnancy, what's the average number of cigarettes you smoked per day?	______________________
During the second three months of pregnancy, what's the average number of cigarettes you smoked per day?	______________________
During the third trimester of pregnancy, what's the average number of cigarettes you smoked per day?	______________________
How many people, not including yourself, smoke inside your home most days? (Include family members, friends and anyone else)	______________________
During any of your visits to a doctor, nurse or other medical professional, did any of these medical professionals recommend that you get a flu shot or tell you that you need a flu shot?	◯ No<br>◯ Yes<br>◯ Unsure/ Unknown<br>◯ Refused/ No Response
During any of your visits to a doctor, nurse or other medical professional, did any of these medical professionals offer to give you a flu shot or tell you that you needed a flu shot?	◯ No<br>◯ Yes<br>◯ Unsure/ Unknown<br>◯ Refused/ No Response
Did you get a flu shot during this pregnancy?	◯ No<br>◯ Yes<br>◯ Unsure/ Unknown<br>◯ Refused/ No Response
If yes, what was the date of the shot?	______________________
Date Description:	◯ Exact<br>◯ Approximate<br>◯ Unknown<br>◯ No Response/Refused

At what kind of place did you receive the flu vaccination?	◯ My OB/GYN's office<br>◯ My midwife's office<br>◯ My family doctor or other doctor's office<br>◯ Health department clinic<br>◯ Other clinic or health center<br>◯ Hospital<br>◯ Other medically-related place<br>◯ A pharmacy or drug store (like a Walgreens or CVS)<br>◯ A supermarket, grocery store, or superstore (like a Wal-Mart or Target)<br>◯ Workplace<br>◯ Elementary/Middle/High School<br>◯ Other non-medically related place<br>◯ Unsure/Unknown
Please specify "other medically-related place":	__________________________________
Please specify "other non-medically related place":	__________________________________
Please provide the name and location of the vaccination provider:	__________________________________
Did you get a flu shot in the past year, before you became pregnant with this current pregnancy?	◯ No<br>◯ Yes<br>◯ Unsure/Unknown<br>◯ Refused/ No Response
If yes, what was the date of the shot?	__________________________________
Date Description:	◯ Exact<br>◯ Approximate<br>◯ Unknown<br>◯ No Response/Refused
If yes, which of the following is the ONE main reason why you decided to be vaccinated for the flu during this pregnancy?	◯ To protect myself from the flu<br>◯ To protect my baby from the flu<br>◯ To protect my friends and family from the flu<br>◯ A doctor, nurse, or other medical professional recommended the flu shot<br>◯ To avoid missing work or because I had to for work<br>◯ Because the flu might be bad this season<br>◯ It was easy or convenient to get the flu shot<br>◯ Unknown, Unsure<br>◯ Refused/ No Response
Were any of the other options reasons why you decided to be vaccinated for the flu during your pregnancy?<br><br>[Exclude the answer given above; the study participant may choose as many additional answers as applicable]	☐ To protect myself from the flu<br>☐ To protect my baby from the flu<br>☐ To protect my friends and family from the flu<br>☐ A doctor, nurse, or other medical professional recommended the flu shot<br>☐ To avoid missing work or because I had to for work<br>☐ Because the flu might be bad this season<br>☐ It was easy or convenient to get the flu shot<br>☐ Unknown, Unsure<br>☐ Refused/ No Response

How effective do you think the flu shot is in preventing you from getting the flu?	◯ Very effective<br>◯ Somewhat effective<br>◯ Not too effective<br>◯ Not at all effective<br>◯ Unknown/ Unsure<br>◯ Refused/ No Response
When a pregnant woman receives the flu vaccination, how effective do you think it is in protecting her baby from the flu?	◯ Very effective<br>◯ Somewhat effective<br>◯ Not too effective<br>◯ Not at all effective<br>◯ Unknown/ Unsure<br>◯ Refused/ No Response
Based on what you know or have heard, how safe are flu shots for most adult women?	◯ Completely safe<br>◯ Very safe<br>◯ Somewhat safe<br>◯ Somewhat unsafe<br>◯ Very unsafe<br>◯ Completely unsafe<br>◯ Unknown/ Unsure<br>◯ Refused/ No Response
How safe do you think flu vaccinations are for pregnant women?	◯ Completely safe<br>◯ Very safe<br>◯ Somewhat safe<br>◯ Somewhat unsafe<br>◯ Very unsafe<br>◯ Completely unsafe<br>◯ Unknown/ Unsure<br>◯ Refused/ No Response
When a pregnant woman receives the flu vaccination, how safe do you think that vaccination is for her baby?	◯ Completely safe<br>◯ Very safe<br>◯ Somewhat safe<br>◯ Somewhat unsafe<br>◯ Very unsafe<br>◯ Completely unsafe<br>◯ Unknown/ Unsure<br>◯ Refused/ No Response
During this pregnancy, did a doctor, nurse or health care worker offer you a Tdap shot or tell you to get one?	◯ No<br>◯ Yes<br>◯ Unsure/ Unknown<br>◯ Refused/ No Response
Did you get a Tdap shot during this pregnancy?	◯ No<br>◯ Yes<br>◯ Unsure/ Unknown<br>◯ Refused/ No Response
If yes, what was the date when you received the tdap shot?	__________________________________
Date Description:	◯ Exact<br>◯ Approximate<br>◯ Unknown<br>◯ No Response/Refused

Have you ever refused a vaccination for yourself?	◯ No<br>◯ Yes<br>◯ Unsure/ Unknown<br>◯ Refused/ No Response
If you have a previous child, have you ever refused a vaccination for a child of yours?	◯ No<br>◯ Yes<br>◯ Unsure/ Unknown<br>◯ Refused/ No Response

Please choose the answer that most closely matches your opinion:

	Strongly agree	Agree	Not sure	Disagree	Strongly disagree
I trust the information I receive about shots	◯	◯	◯	◯	◯
It is better for my child to develop immunity by getting sick than to get a shot	◯	◯	◯	◯	◯
It is better for children to get fewer shots at the same time	◯	◯	◯	◯	◯
Children get more shots than are good for them	◯	◯	◯	◯	◯

Please choose the answer that most closely matches your opinion

	Not at all hesitant	Not too hesitant	Not sure	Somewhat hesitant	Very hesitant
Overall, how hesitant about childhood shots would you consider yourself to be?	◯	◯	◯	◯	◯

Over the past 30 days, on average how many hours per day did you spend out of doors for work, school or recreationally? Would you say...?	◯ Less than 1 hour a day<br>◯ 1 hour a day<br>◯ 2 hours a day<br>◯ 3 hours a day<br>◯ 4 hours a day<br>◯ 5 hours a day<br>◯ Unknown/ Unsure<br>◯ Refused/ No Response

You may not know exactly what your plans are for feeding your baby, but you may have ideas about what you would like or are planning to do. I am going to read you statements about feeding your baby and I would like you to please choose the answer that most closely matches your opinion, considering both your current feeding plans and the likelihood you will carry out those plans.

	Very much agree	Somewhat agree	Unsure	Somewhat disagree	Very much disagree

I am planning to only formula feed my baby (I will not breastfeed at all)	◯	◯	◯	◯	◯
I am planning to breastfeed my baby or at least try.	◯	◯	◯	◯	◯
When my baby is one month old, I will be breastfeeding my baby without any formula or other milk.	◯	◯	◯	◯	◯
When my baby is three months old, I will be breastfeeding my baby without using any formula or other milk.	◯	◯	◯	◯	◯
When my baby is six months old, I will be breastfeeding my baby without using any formula or other milk.	◯	◯	◯	◯	◯

How old do you think your baby will be when you completely stop breastfeeding?

____
(months)

How comfortable are you with the idea of breastfeeding your baby?

◯ Very comfortable
◯ Somewhat comfortable
◯ Somewhat uncomfortable
◯ Very uncomfortable
◯ Unknown/ Unsure
◯ Refused/ No Response

During this pregnancy, have you received any counseling on how best to feed your infant?

◯ No
◯ Yes
◯ Unknown/ Unsure
◯ Refused/ No Response

If yes, what best describes the advice you received.

◯ Breastfeeding is recommended
◯ Formula is recommended
◯ Both breastfeeding and formula are recommended
◯ Unknown, Unsure
◯ Refused/ No Response
