# Supplementary material for: Pediatric Respiratory and Enteric Virus Acquisition and Immunogenesis in US Mothers and Children Aged 0-2: PREVAIL Cohort Study
Source: JMIR Res Protoc. 2021 Feb 12;10(2):e22222. doi: 10.2196/22222 (PMC7910118; doi:10.2196/22222)
Supplement: Multimedia Appendix 4 [file resprot_v10i2e22222_app4.pdf]

Follow-up Week 2

Please complete the survey below.

Thank you!

Subject ID ([consent_arm_1][subjectid]) ______________________

Follow-up attempts

Mother's primary phone number: [consent_arm_1][mother_phone_1] ______________________

Mother's Name: [consent_arm_1][mother_first_name] [consent_arm_1][mother_last_name]

Date ______________________

CRC Initials ______________________

After mother completes the EPDS, please review her score:

0 - 9 points: Low probability of depression

10 - 30: Possible to high probability of depression

ALWAYS look at question 10 - suicidal ideation

Thank you for agreeing to participate in the PREVAIL study and for allowing me to visit your home. I am going to ask you a series of questions that are part of the "Follow-up Questionnaire." These questions will help us to collect information about your health and the health of your child. At future study visits, we will ask you some of these questions again to update the information as your child grows.

Mother's Name: [consent_arm_1][mother_first_name] [consent_arm_1][mother_last_name]

Date of Enrollment: [consent_arm_1][bl_date]

Confirm current address ______________________

Street

Baseline
[consent_arm_1][address_street]

Latitude ______________________

Longitude ______________________

Census Tract ID ______________________

Deprivation Index Score ______________________

Infant Feeding

Now I am going to ask you a few questions about how you have been feeding your infant since birth

Since delivery, have you ever breastfed or pumped breast milk to feed [week_0_arm_1][nbcrf_inf_name]?
- ◯ No
- ◯ Yes
- ◯ Unsure/Unknown
- ◯ Refused/No Response

Are you still feeding breast milk to [week_0_arm_1][nbcrf_inf_name]?

This includes breast milk in ANY amount, whether exclusively or intermittently
- ◯ No
- ◯ Yes
- ◯ Unsure/Unknown
- ◯ Refused/No Response

If no, what was the date you stopped any breastfeeding?

____

Since [week_0_arm_1][nbcrf_inf_name] was born, which of the following best describes the type of milk [week_0_arm_1][nbcrf_inf_name] was fed?
- ◯ All breast milk - your infant has NEVER been fed formula or other milk
- ◯ Mostly breast milk, some formula or other milk
- ◯ About half breast milk, half formula or other milk
- ◯ Mostly formula or other milk, some breast milk
- ◯ All formula or other milk - your infant has NEVER been fed breast milk

Since delivery, how has your breast milk usually been fed to [week_0_arm_1][nbcrf_inf_name]?
- ◯ At the breast
- ◯ Pumped, then fed by a bottle
- ◯ A combination of at the breast and pumped milk

Has [week_0_arm_1][nbcrf_inf_name] ever been fed formula?
- ◯ No
- ◯ Yes
- ◯ Unsure/Unknown
- ◯ Refused/No Response

On what date was formula first introduced?

____
(Use Calendar to assist the mother)

Since this time yesterday, how many times has[week_0_arm_1][nbcrf_inf_name] been fed the following:

(If the child has not been fed an item, enter the number zero)

Breast milk

____

Juice

____

Infant formula

____

Baby food

____

Water

____

Soda ____________________

Tea ____________________

Other ____________________

If "other", describe: ________________________

Lactogenesis & Breastfeeding Concerns

Most women will experience a noticeable change in their breast fullness a few days after giving birth as compared to before giving birth. This happens when the breasts change from making small amounts of colostrum to making larger amounts of mature milk. This change is often referred to as the milk "coming in."

Do you remember a noticeable increase in the feeling of your breast fullness after giving birth?
- ◯ No
- ◯ Yes
- ◯ Unsure/Unknown
- ◯ Refused/No Response

If yes or unsure, about how many hours after the delivery of your baby did you notice that your breasts were becoming fuller or heavier?
- ◯ Unsure/Unknown
- ◯ 0-23.9 hours
- ◯ 24-47.9 hours
- ◯ 48-71.9 hours
- ◯ 72-95.9 hours
- ◯ 96 or more hours
- ◯ Refused/ No Response

Please rate, from 1-5, the maximum breast fullness or heaviness you have experienced as compared to before giving birth
- ◯ Unsure/Unknown
- ◯ 1 - No change
- ◯ 2
- ◯ 3 - Noticeably fuller
- ◯ 4
- ◯ 5 - Uncomfortably full or heavy
- ◯ Refused/No response

Since your baby was born, have you had any breastfeeding difficulties or concerns?
- ◯ Yes, I am currently concerned
- ◯ Yes, for a while, but I am no longer concerned
- ◯ No

Since your baby was born, have you been concerned about having enough breast milk for your baby?
- ◯ Yes, I am currently concerned
- ◯ Yes, for a while, but I am no longer concerned
- ◯ No

Since your baby was born, have you experienced breast/nipple pain or discomfort?
- ◯ Yes, I am currently concerned
- ◯ Yes, for a while, but I am no longer concerned
- ◯ No

Maternal Immunization

If the mother reported NOT receiving a flu or Tdap vaccine on her baseline questionnaire AND there is no record of either vaccination in her maternal immunization record in the OB CRF, please ask the follow-up question(s) below:

After you were enrolled in this study, did you get a flu shot during the rest of your pregnancy?	◯ No<br>◯ Yes<br>◯ Not applicable, flu shot already administered<br>◯ Unsure/ Unknown<br>◯ Refused/ No response
If yes, when was the flu shot administered?	__________________________________
Date Description:	◯ Exact<br>◯ Approximate<br>◯ Unknown<br>◯ No Response/Refused
If yes, which of the following is the ONE main reason why you decided to be vaccinated for the flu during your pregnancy?	◯ To protect myself from the flu<br>◯ To protect my baby from the flu<br>◯ To protect my friends and family from the flu<br>◯ A doctor, nurse or other medical professional recommended the flu shot<br>◯ To avoid missing work or because I had to for work<br>◯ Because the flu might be bad this season<br>◯ It was easy or convenient to get the flu shot<br>◯ Unknown/Unsure<br>◯ Refused/ No response
Similarly, since enrollment, have you received a Tdap shot?	◯ No<br>◯ Yes<br>◯ Not applicable, Tdap shot already administered<br>◯ Unsure/ Unknown<br>◯ Refused/ No response
If yes, when was the Tdap administered?	__________________________________
Date Description:	◯ Exact<br>◯ Approximate<br>◯ Unknown<br>◯ No Response/Refused
Since your enrollment and your first questionnaire, have you had symptoms of a respiratory illness such as a fever (or felt feverish), cough, earache, nasal congestion, runny nose, shortness of breath, rapid or shallow breathing, sore throat, vomiting after cough, or wheezing?	◯ No<br>◯ Yes<br>◯ Unsure/Unknown<br>◯ Refused/No Response
If yes, did you have a fever with this illness?	◯ No<br>◯ Yes<br>◯ Unsure/Unknown<br>◯ Refused/No Response

If yes, how many times did you feel sick?	◯ 1<br>◯ 2<br>◯ 3<br>◯ 4 or more times
Please provides the dates that you felt ill	
(1) Date	__________________________
(1) Date Description	◯ Exact<br>◯ Approximate<br>◯ Unknown<br>◯ No Response/Refused
(2) Date	__________________________
(2) Date Description	◯ Exact<br>◯ Approximate<br>◯ Unknown<br>◯ No Response/Refused
(3) Date	__________________________
(3) Date Description	◯ Exact<br>◯ Approximate<br>◯ Unknown<br>◯ No Response/Refused
If you felt sick on more than 4 occasions, please provide the dates and date descriptions here:	________________________________
If yes, did any of these illnesses result in a doctor's visit or hospital stay?	◯ No<br>◯ Yes, doctor's visit<br>◯ Yes, hospitalization<br>◯ Unsure/Unknown<br>◯ Refused/No Response
If yes, did you take oseltamivir (Tamiflu or generic version) or a similar specific prescription antiviral medication as treatment for your illness	◯ No<br>◯ Yes<br>◯ Unsure/Unknown<br>◯ Refused/No Response
If yes, what medication did you take?	◯ Oseltamivir (generic version or Tamiflu)<br>◯ Zanamivir (Relenza)<br>◯ Unsure/Unknown<br>◯ Refused/ No Response

Edinburg Postnatal Depression Scale (EPDS)

Since you recently had a baby, we would like to know how you are feeling. Please give the answer that comes closest to how you have felt in the past 7 days, not just how you feel today.

I have been able to laugh and see the funny side of things.
- ◯ As much as I always could
- ◯ Not quite so much now
- ◯ Definitely not so much now
- ◯ Not at all

I have looked forward with enjoyment to things.
- ◯ As much as I ever did
- ◯ Rather less than I used to
- ◯ Definitely less than I used to
- ◯ Hardly at all

I have blamed myself unnecessarily when things went wrong.
- ◯ Yes, most of the time
- ◯ Yes, some of the time
- ◯ Not very often
- ◯ No never

I have been anxious or worried for no good reason.
- ◯ No not at all
- ◯ Hardly ever
- ◯ Yes, sometimes
- ◯ Yes, very often

I have felt scared or panicky for no very good reason.
- ◯ Yes, quite a lot
- ◯ Yes, sometimes
- ◯ No, not much
- ◯ No, not at all

Things have been getting on top of me.
- ◯ Yes, most of the time I haven't been able to cope at all
- ◯ Yes, sometimes I haven't been coping as well as usual
- ◯ No, most of the time I have coped quite well
- ◯ No, I have been coping as well as ever

I have been so unhappy that I have had difficulty sleeping.
- ◯ Yes, most of the time
- ◯ Yes, sometimes
- ◯ Not very often
- ◯ No, not at all

I have felt sad or miserable.
- ◯ Yes, most of the time
- ◯ Yes, quite often
- ◯ Not very often
- ◯ No, not at all

I have been so unhappy that I have been crying.
- ◯ Yes, most of the time
- ◯ Yes, quite often
- ◯ Only occasionally
- ◯ No, never

The thought of harming myself has occurred to me.
- ◯ Yes, quite often
- ◯ Sometimes
- ◯ Hardly ever
- ◯ Never

EPDS Score

____
(Minimum=0 Maximum=30)

Maternal Medication Use

Types of Medications

Since your child was born, have you taken any of the following:

Vitamins or dietary supplements
- ◯ No
- ◯ Yes
- ◯ Unsure/Unknown
- ◯ Refused/No Response

Over the counter medications
- ◯ No
- ◯ Yes
- ◯ Unsure/Unknown
- ◯ Refused/No Response

Herbal or folk remedies
- ◯ No
- ◯ Yes
- ◯ Unsure/Unknown
- ◯ Refused/No Response

Prescription medications
- ◯ No
- ◯ Yes
- ◯ Unsure/Unknown
- ◯ Refused/No Response

Maternal Medication List

How many vitamins/dietary supplements, over the counter medications, herbal/folk remedies or prescription medications is the mother taking in total?
- ◯ 1
- ◯ 2
- ◯ 3
- ◯ 4
- ◯ 5
- ◯ 6
- ◯ 7
- ◯ 8
- ◯ 9
- ◯ 10
- ◯ 11 or More

Maternal Medication 1

(1) Medication Name	◯ DHA<br>◯ Docusate sodium<br>◯ Ibuprofen<br>◯ Iron<br>◯ Miralax<br>◯ Oxycodone- acetaminophen (percocet)<br>◯ Prenatal vitamins<br>◯ Prenatal vitamins + DHA<br>◯ Tylenol<br>◯ Vitamin D<br>◯ Antimicrobial<br>◯ Other vitamin<br>◯ Birth control pills<br>◯ Antihistamine<br>◯ Herbal Supplement<br>◯ Other<br>◯ Unsure/ Unknown<br>◯ Refused/ No Response
(1) If medication is "antimicrobial" please describe	◯ Acyclovir<br>◯ Amoxicillin<br>◯ Ancef/ Cefazolin<br>◯ Azithromycin<br>◯ Clindamycin<br>◯ Diflucan/ Fluconazole<br>◯ Flagyl<br>◯ Macrobid<br>◯ Penicillin G<br>◯ Vancomycin<br>◯ Oseltamivir/Tamiflu<br>◯ Other<br>◯ Unsure/ Unknown<br>◯ No Response/ Refused
(1) If medication is "other", please describe:	__________________________________
(1) Dosage/Strength	__________________________________
(1) Units	◯ Capsule<br>◯ Cup<br>◯ Drop<br>◯ Gram<br>◯ Kilogram<br>◯ Liter<br>◯ Microgram<br>◯ Milligram<br>◯ Milliliter<br>◯ Ounce<br>◯ Pint<br>◯ Puff<br>◯ Quart<br>◯ Tablespoon<br>◯ Teaspoon<br>◯ Unit<br>◯ Other<br>◯ Unknowns/ Unsure<br>◯ No Response/ Refused
(1) If units is "other", please describe:	__________________________________

(1) Route	◯ Buccal<br>◯ Enteral<br>◯ Intradermal<br>◯ Intramuscular<br>◯ Intranasal<br>◯ Intravenous<br>◯ Ophthalmic<br>◯ Oral<br>◯ Otic<br>◯ Rectal<br>◯ Subcutaneous<br>◯ Sublingual<br>◯ Topical<br>◯ Transdermal<br>◯ Vaginally<br>◯ Other<br>◯ Unknown/ Unsure<br>◯ No Response/ Refused
(1) If route is "other", please describe:	__________________________________
(1) Frequency	◯ QD<br>◯ PRN<br>◯ BID<br>◯ TID<br>◯ QID<br>◯ Q 1 Hour<br>◯ Q 2 Hours<br>◯ Q 3 Hours<br>◯ Q 4 Hours<br>◯ Q 4-6 Hours<br>◯ Q 6 Hours<br>◯ Q 8 Hours<br>◯ Q 12 Hours<br>◯ QD PRN<br>◯ BID PRN<br>◯ TID PRN<br>◯ QID PRN<br>◯ Q 1 Hour PRN<br>◯ Q 2 Hours PRN<br>◯ Q 3 Hours PRN<br>◯ Q 4 Hours PRN<br>◯ Q 4-6 Hours PRN<br>◯ Q 6 Hours PRN<br>◯ Q 8 Hours PRN<br>◯ Q 12 Hours PRN<br>◯ Other<br>◯ Unknown/ Unsure<br>◯ Refused/ No Response
(1) If frequency is "other", please describe:	__________________________________

(1) Indication	◯ Anti-Inflammatory<br>◯ Constipation<br>◯ Depression/Anxiety<br>◯ Headache<br>◯ Low Iron<br>◯ Pain<br>◯ Vitamins<br>◯ Allergies<br>◯ Hypertension<br>◯ Birth Control<br>◯ Lactation Support<br>◯ Mastitis<br>◯ Influenza<br>◯ Other<br>◯ Unknown/ Unsure<br>◯ No Response/ Refused
(1) If indication is "other", please describe:	__________________________________
Maternal Medication 2	
(2) Medication Name	◯ DHA<br>◯ Docusate sodium<br>◯ Ibuprofen<br>◯ Iron<br>◯ Miralax<br>◯ Oxycodone- acetaminophen (percocet)<br>◯ Prenatal vitamins<br>◯ Prenatal vitamins + DHA<br>◯ Tylenol<br>◯ Vitamin D<br>◯ Antimicrobial<br>◯ Other vitamin<br>◯ Birth control pills<br>◯ Antihistamine<br>◯ Herbal Supplement<br>◯ Other<br>◯ Unsure/ Unknown<br>◯ Refused/ No Response
(2) If medication is "antimicrobial" please describe	◯ Acyclovir<br>◯ Amoxicillin<br>◯ Ancef/ Cefazolin<br>◯ Azithromycin<br>◯ Clindamycin<br>◯ Diflucan/ Fluconazole<br>◯ Flagyl<br>◯ Macrobid<br>◯ Penicillin G<br>◯ Vancomycin<br>◯ Oseltamivir/Tamiflu<br>◯ Other<br>◯ Unsure/ Unknown<br>◯ No Response/ Refused
(2) If medication is "other", please describe:	__________________________________
(2) Dosage/Strength	__________________________________

(2) Units

- ◯ Capsule
- ◯ Cup
- ◯ Drop
- ◯ Gram
- ◯ Kilogram
- ◯ Liter
- ◯ Microgram
- ◯ Milligram
- ◯ Milliliter
- ◯ Ounce
- ◯ Pint
- ◯ Puff
- ◯ Quart
- ◯ Tablespoon
- ◯ Teaspoon
- ◯ Unit
- ◯ Other
- ◯ Unknowns/ Unsure
- ◯ No Response/ Refused

(2) If units is "other", please describe: ______________________

(2) Route

- ◯ Buccal
- ◯ Enteral
- ◯ Intradermal
- ◯ Intramuscular
- ◯ Intranasal
- ◯ Intravenous
- ◯ Ophthalmic
- ◯ Oral
- ◯ Otic
- ◯ Rectal
- ◯ Subcutaneous
- ◯ Sublingual
- ◯ Topical
- ◯ Transdermal
- ◯ Vaginally
- ◯ Other
- ◯ Unknown/ Unsure
- ◯ No Response/ Refused

(2) If route is "other", please describe: ______________________

(2) Frequency	◯ QD<br>◯ PRN<br>◯ BID<br>◯ TID<br>◯ QID<br>◯ Q 1 Hour<br>◯ Q 2 Hours<br>◯ Q 3 Hours<br>◯ Q 4 Hours<br>◯ Q 4-6 Hours<br>◯ Q 6 Hours<br>◯ Q 8 Hours<br>◯ Q 12 Hours<br>◯ QD PRN<br>◯ BID PRN<br>◯ TID PRN<br>◯ QID PRN<br>◯ Q 1 Hour PRN<br>◯ Q 2 Hours PRN<br>◯ Q 3 Hours PRN<br>◯ Q 4 Hours PRN<br>◯ Q 4-6 Hours PRN<br>◯ Q 6 Hours PRN<br>◯ Q 8 Hours PRN<br>◯ Q 12 Hours PRN<br>◯ Other<br>◯ Unknown/ Unsure<br>◯ Refused/ No Response
(2) If frequency is "other", please describe:	__________________________________
(2) Indication	◯ Anti-Inflammatory<br>◯ Constipation<br>◯ Depression/Anxiety<br>◯ Headache<br>◯ Low Iron<br>◯ Pain<br>◯ Vitamins<br>◯ Allergies<br>◯ Hypertension<br>◯ Birth Control<br>◯ Lactation Support<br>◯ Mastitis<br>◯ Influenza<br>◯ Other<br>◯ Unknown/ Unsure<br>◯ No Response/ Refused
(2) If indication is "other", please describe:	__________________________________
Maternal Medication 3	

(3) Medication Name	◯ DHA<br>◯ Docusate sodium<br>◯ Ibuprofen<br>◯ Iron<br>◯ Miralax<br>◯ Oxycodone- acetaminophen (percocet)<br>◯ Prenatal vitamins<br>◯ Prenatal vitamins + DHA<br>◯ Tylenol<br>◯ Vitamin D<br>◯ Antimicrobial<br>◯ Other vitamin<br>◯ Birth control pills<br>◯ Antihistamine<br>◯ Herbal Supplement<br>◯ Other<br>◯ Unsure/ Unknown<br>◯ Refused/ No Response
(3) If medication is "antimicrobial" please describe	◯ Acyclovir<br>◯ Amoxicillin<br>◯ Ancef/ Cefazolin<br>◯ Azithromycin<br>◯ Clindamycin<br>◯ Diflucan/ Fluconazole<br>◯ Flagyl<br>◯ Macrobid<br>◯ Penicillin G<br>◯ Vancomycin<br>◯ Oseltamivir/Tamiflu<br>◯ Other<br>◯ Unsure/ Unknown<br>◯ No Response/ Refused
(3) If medication is "other", please describe:	__________________________________
(3) Dosage/Strength	__________________________________
(3) Units	◯ Capsule<br>◯ Cup<br>◯ Drop<br>◯ Gram<br>◯ Kilogram<br>◯ Liter<br>◯ Microgram<br>◯ Milligram<br>◯ Milliliter<br>◯ Ounce<br>◯ Pint<br>◯ Puff<br>◯ Quart<br>◯ Tablespoon<br>◯ Teaspoon<br>◯ Unit<br>◯ Other<br>◯ Unknowns/ Unsure<br>◯ No Response/ Refused
(3) If units is "other", please describe:	__________________________________

(3) Route	◯ Buccal<br>◯ Enteral<br>◯ Intradermal<br>◯ Intramuscular<br>◯ Intranasal<br>◯ Intravenous<br>◯ Ophthalmic<br>◯ Oral<br>◯ Otic<br>◯ Rectal<br>◯ Subcutaneous<br>◯ Sublingual<br>◯ Topical<br>◯ Transdermal<br>◯ Vaginally<br>◯ Other<br>◯ Unknown/ Unsure<br>◯ No Response/ Refused
(3) If route is "other", please describe:	__________________________________
(3) Frequency	◯ QD<br>◯ PRN<br>◯ BID<br>◯ TID<br>◯ QID<br>◯ Q 1 Hour<br>◯ Q 2 Hours<br>◯ Q 3 Hours<br>◯ Q 4 Hours<br>◯ Q 4-6 Hours<br>◯ Q 6 Hours<br>◯ Q 8 Hours<br>◯ Q 12 Hours<br>◯ QD PRN<br>◯ BID PRN<br>◯ TID PRN<br>◯ QID PRN<br>◯ Q 1 Hour PRN<br>◯ Q 2 Hours PRN<br>◯ Q 3 Hours PRN<br>◯ Q 4 Hours PRN<br>◯ Q 4-6 Hours PRN<br>◯ Q 6 Hours PRN<br>◯ Q 8 Hours PRN<br>◯ Q 12 Hours PRN<br>◯ Other<br>◯ Unknown/ Unsure<br>◯ Refused/ No Response
(3) If frequency is "other", please describe:	__________________________________

(3) Indication	◯ Anti-Inflammatory<br>◯ Constipation<br>◯ Depression/Anxiety<br>◯ Headache<br>◯ Low Iron<br>◯ Pain<br>◯ Vitamins<br>◯ Allergies<br>◯ Hypertension<br>◯ Birth Control<br>◯ Lactation Support<br>◯ Mastitis<br>◯ Influenza<br>◯ Other<br>◯ Unknown/ Unsure<br>◯ No Response/ Refused
(3) If indication is "other", please describe:	__________________________
Maternal Medication 4	
(4) Medication Name	◯ DHA<br>◯ Docusate sodium<br>◯ Ibuprofen<br>◯ Iron<br>◯ Miralax<br>◯ Oxycodone- acetaminophen (percocet)<br>◯ Prenatal vitamins<br>◯ Prenatal vitamins + DHA<br>◯ Tylenol<br>◯ Vitamin D<br>◯ Antimicrobial<br>◯ Other vitamin<br>◯ Birth control pills<br>◯ Antihistamine<br>◯ Herbal Supplement<br>◯ Other<br>◯ Unsure/ Unknown<br>◯ Refused/ No Response
(4) If medication is "antimicrobial" please describe	◯ Acyclovir<br>◯ Amoxicillin<br>◯ Ancef/ Cefazolin<br>◯ Azithromycin<br>◯ Clindamycin<br>◯ Diflucan/ Fluconazole<br>◯ Flagyl<br>◯ Macrobid<br>◯ Penicillin G<br>◯ Vancomycin<br>◯ Oseltamivir/Tamiflu<br>◯ Other<br>◯ Unsure/ Unknown<br>◯ No Response/ Refused
(4) If medication is "other", please describe:	__________________________
(4) Dosage/Strength	__________________________

(4) Units	◯ Capsule<br>◯ Cup<br>◯ Drop<br>◯ Gram<br>◯ Kilogram<br>◯ Liter<br>◯ Microgram<br>◯ Milligram<br>◯ Milliliter<br>◯ Ounce<br>◯ Pint<br>◯ Puff<br>◯ Quart<br>◯ Tablespoon<br>◯ Teaspoon<br>◯ Unit<br>◯ Other<br>◯ Unknowns/ Unsure<br>◯ No Response/ Refused
(4) If units is "other", please describe:	______________________________
(4) Route	◯ Buccal<br>◯ Enteral<br>◯ Intradermal<br>◯ Intramuscular<br>◯ Intranasal<br>◯ Intravenous<br>◯ Ophthalmic<br>◯ Oral<br>◯ Otic<br>◯ Rectal<br>◯ Subcutaneous<br>◯ Sublingual<br>◯ Topical<br>◯ Transdermal<br>◯ Vaginally<br>◯ Other<br>◯ Unknown/ Unsure<br>◯ No Response/ Refused
(4) If route is "other", please describe:	______________________________

(4) Frequency

- ◯ QD
- ◯ PRN
- ◯ BID
- ◯ TID
- ◯ QID
- ◯ Q 1 Hour
- ◯ Q 2 Hours
- ◯ Q 3 Hours
- ◯ Q 4 Hours
- ◯ Q 4-6 Hours
- ◯ Q 6 Hours
- ◯ Q 8 Hours
- ◯ Q 12 Hours
- ◯ QD PRN
- ◯ BID PRN
- ◯ TID PRN
- ◯ QID PRN
- ◯ Q 1 Hour PRN
- ◯ Q 2 Hours PRN
- ◯ Q 3 Hours PRN
- ◯ Q 4 Hours PRN
- ◯ Q 4-6 Hours PRN
- ◯ Q 6 Hours PRN
- ◯ Q 8 Hours PRN
- ◯ Q 12 Hours PRN
- ◯ Other
- ◯ Unknown/ Unsure
- ◯ Refused/ No Response

(4) If frequency is "other", please describe: ______________________________

(4) Indication

- ◯ Anti-Inflammatory
- ◯ Constipation
- ◯ Depression/Anxiety
- ◯ Headache
- ◯ Low Iron
- ◯ Pain
- ◯ Vitamins
- ◯ Allergies
- ◯ Hypertension
- ◯ Birth Control
- ◯ Lactation Support
- ◯ Mastitis
- ◯ Influenza
- ◯ Other
- ◯ Unknown/ Unsure
- ◯ No Response/ Refused

(4) If indication is "other", please describe: ______________________________

Maternal Medication 5

(5) Medication Name	◯ DHA<br>◯ Docusate sodium<br>◯ Ibuprofen<br>◯ Iron<br>◯ Miralax<br>◯ Oxycodone- acetaminophen (percocet)<br>◯ Prenatal vitamins<br>◯ Prenatal vitamins + DHA<br>◯ Tylenol<br>◯ Vitamin D<br>◯ Antimicrobial<br>◯ Other vitamin<br>◯ Birth control pills<br>◯ Antihistamine<br>◯ Herbal Supplement<br>◯ Other<br>◯ Unsure/ Unknown<br>◯ Refused/ No Response
(5) If medication is "antimicrobial" please describe	◯ Acyclovir<br>◯ Amoxicillin<br>◯ Ancef/ Cefazolin<br>◯ Azithromycin<br>◯ Clindamycin<br>◯ Diflucan/ Fluconazole<br>◯ Flagyl<br>◯ Macrobid<br>◯ Penicillin G<br>◯ Vancomycin<br>◯ Oseltamivir/Tamiflu<br>◯ Other<br>◯ Unsure/ Unknown<br>◯ No Response/ Refused
(5) If medication is "other", please describe:	________________________
(5) Dosage/Strength	________________________
(5) Units	◯ Capsule<br>◯ Cup<br>◯ Drop<br>◯ Gram<br>◯ Kilogram<br>◯ Liter<br>◯ Microgram<br>◯ Milligram<br>◯ Milliliter<br>◯ Ounce<br>◯ Pint<br>◯ Puff<br>◯ Quart<br>◯ Tablespoon<br>◯ Teaspoon<br>◯ Unit<br>◯ Other<br>◯ Unknowns/ Unsure<br>◯ No Response/ Refused
(5) If units is "other", please describe:	________________________

(5) Route	◯ Buccal<br>◯ Enteral<br>◯ Intradermal<br>◯ Intramuscular<br>◯ Intranasal<br>◯ Intravenous<br>◯ Ophthalmic<br>◯ Oral<br>◯ Otic<br>◯ Rectal<br>◯ Subcutaneous<br>◯ Sublingual<br>◯ Topical<br>◯ Transdermal<br>◯ Vaginally<br>◯ Other<br>◯ Unknown/ Unsure<br>◯ No Response/ Refused
(5) If route is "other", please describe:	__________________________________
(5) Frequency	◯ QD<br>◯ PRN<br>◯ BID<br>◯ TID<br>◯ QID<br>◯ Q 1 Hour<br>◯ Q 2 Hours<br>◯ Q 3 Hours<br>◯ Q 4 Hours<br>◯ Q 4-6 Hours<br>◯ Q 6 Hours<br>◯ Q 8 Hours<br>◯ Q 12 Hours<br>◯ QD PRN<br>◯ BID PRN<br>◯ TID PRN<br>◯ QID PRN<br>◯ Q 1 Hour PRN<br>◯ Q 2 Hours PRN<br>◯ Q 3 Hours PRN<br>◯ Q 4 Hours PRN<br>◯ Q 4-6 Hours PRN<br>◯ Q 6 Hours PRN<br>◯ Q 8 Hours PRN<br>◯ Q 12 Hours PRN<br>◯ Other<br>◯ Unknown/ Unsure<br>◯ Refused/ No Response
(5) If frequency is "other", please describe:	__________________________________

(5) Indication

- ◯ Anti-Inflammatory
- ◯ Constipation
- ◯ Depression/Anxiety
- ◯ Headache
- ◯ Low Iron
- ◯ Pain
- ◯ Vitamins
- ◯ Allergies
- ◯ Hypertension
- ◯ Birth Control
- ◯ Lactation Support
- ◯ Mastitis
- ◯ Influenza
- ◯ Other
- ◯ Unknown/ Unsure
- ◯ No Response/ Refused

(5) If indication is "other", please describe: ________________________________

Maternal Medication 6

(6) Medication Name

- ◯ DHA
- ◯ Docusate sodium
- ◯ Ibuprofen
- ◯ Iron
- ◯ Miralax
- ◯ Oxycodone- acetaminophen (percocet)
- ◯ Prenatal vitamins
- ◯ Prenatal vitamins + DHA
- ◯ Tylenol
- ◯ Vitamin D
- ◯ Antimicrobial
- ◯ Other vitamin
- ◯ Birth control pills
- ◯ Antihistamine
- ◯ Herbal Supplement
- ◯ Other
- ◯ Unsure/ Unknown
- ◯ Refused/ No Response

(6) If medication is "antimicrobial" please describe

- ◯ Acyclovir
- ◯ Amoxicillin
- ◯ Ancef/ Cefazolin
- ◯ Azithromycin
- ◯ Clindamycin
- ◯ Diflucan/ Fluconazole
- ◯ Flagyl
- ◯ Macrobid
- ◯ Penicillin G
- ◯ Vancomycin
- ◯ Oseltamivir/Tamiflu
- ◯ Other
- ◯ Unsure/ Unknown
- ◯ No Response/ Refused

(6) If medication is "other", please describe: ________________________________

(6) Dosage/Strength ________________________________

(6) Units	◯ Capsule<br>◯ Cup<br>◯ Drop<br>◯ Gram<br>◯ Kilogram<br>◯ Liter<br>◯ Microgram<br>◯ Milligram<br>◯ Milliliter<br>◯ Ounce<br>◯ Pint<br>◯ Puff<br>◯ Quart<br>◯ Tablespoon<br>◯ Teaspoon<br>◯ Unit<br>◯ Other<br>◯ Unknowns/ Unsure<br>◯ No Response/ Refused
(6) If units is "other", please describe:	__________________________________
(6) Route	◯ Buccal<br>◯ Enteral<br>◯ Intradermal<br>◯ Intramuscular<br>◯ Intranasal<br>◯ Intravenous<br>◯ Ophthalmic<br>◯ Oral<br>◯ Otic<br>◯ Rectal<br>◯ Subcutaneous<br>◯ Sublingual<br>◯ Topical<br>◯ Transdermal<br>◯ Vaginally<br>◯ Other<br>◯ Unknown/ Unsure<br>◯ No Response/ Refused
(6) If route is "other", please describe:	__________________________________

(6) Frequency	◯ QD<br>◯ PRN<br>◯ BID<br>◯ TID<br>◯ QID<br>◯ Q 1 Hour<br>◯ Q 2 Hours<br>◯ Q 3 Hours<br>◯ Q 4 Hours<br>◯ Q 4-6 Hours<br>◯ Q 6 Hours<br>◯ Q 8 Hours<br>◯ Q 12 Hours<br>◯ QD PRN<br>◯ BID PRN<br>◯ TID PRN<br>◯ QID PRN<br>◯ Q 1 Hour PRN<br>◯ Q 2 Hours PRN<br>◯ Q 3 Hours PRN<br>◯ Q 4 Hours PRN<br>◯ Q 4-6 Hours PRN<br>◯ Q 6 Hours PRN<br>◯ Q 8 Hours PRN<br>◯ Q 12 Hours PRN<br>◯ Other<br>◯ Unknown/ Unsure<br>◯ Refused/ No Response
(6) If frequency is "other", please describe:	__________________________________
(6) Indication	◯ Anti-Inflammatory<br>◯ Constipation<br>◯ Depression/Anxiety<br>◯ Headache<br>◯ Low Iron<br>◯ Pain<br>◯ Vitamins<br>◯ Allergies<br>◯ Hypertension<br>◯ Birth Control<br>◯ Lactation Support<br>◯ Mastitis<br>◯ Influenza<br>◯ Other<br>◯ Unknown/ Unsure<br>◯ No Response/ Refused
(6) If indication is "other", please describe:	__________________________________
Maternal Medication 7	

(7) Medication Name	◯ DHA<br>◯ Docusate sodium<br>◯ Ibuprofen<br>◯ Iron<br>◯ Miralax<br>◯ Oxycodone- acetaminophen (percocet)<br>◯ Prenatal vitamins<br>◯ Prenatal vitamins + DHA<br>◯ Tylenol<br>◯ Vitamin D<br>◯ Antimicrobial<br>◯ Other vitamin<br>◯ Birth control pills<br>◯ Antihistamine<br>◯ Herbal Supplement<br>◯ Other<br>◯ Unsure/ Unknown<br>◯ Refused/ No Response
(7) If medication is "antimicrobial" please describe	◯ Acyclovir<br>◯ Amoxicillin<br>◯ Ancef/ Cefazolin<br>◯ Azithromycin<br>◯ Clindamycin<br>◯ Diflucan/ Fluconazole<br>◯ Flagyl<br>◯ Macrobid<br>◯ Penicillin G<br>◯ Vancomycin<br>◯ Oseltamivir/Tamiflu<br>◯ Other<br>◯ Unsure/ Unknown<br>◯ No Response/ Refused
(7) If medication is "other", please describe:	______________________________
(7) Dosage/Strength	______________________________
(7) Units	◯ Capsule<br>◯ Cup<br>◯ Drop<br>◯ Gram<br>◯ Kilogram<br>◯ Liter<br>◯ Microgram<br>◯ Milligram<br>◯ Milliliter<br>◯ Ounce<br>◯ Pint<br>◯ Puff<br>◯ Quart<br>◯ Tablespoon<br>◯ Teaspoon<br>◯ Unit<br>◯ Other<br>◯ Unknowns/ Unsure<br>◯ No Response/ Refused
(7) If units is "other", please describe:	______________________________

(7) Route

◯ Buccal
◯ Enteral
◯ Intradermal
◯ Intramuscular
◯ Intranasal
◯ Intravenous
◯ Ophthalmic
◯ Oral
◯ Otic
◯ Rectal
◯ Subcutaneous
◯ Sublingual
◯ Topical
◯ Transdermal
◯ Vaginally
◯ Other
◯ Unknown/ Unsure
◯ No Response/ Refused

(7) If route is "other", please describe:

____

(7) Frequency

◯ QD
◯ PRN
◯ BID
◯ TID
◯ QID
◯ Q 1 Hour
◯ Q 2 Hours
◯ Q 3 Hours
◯ Q 4 Hours
◯ Q 4-6 Hours
◯ Q 6 Hours
◯ Q 8 Hours
◯ Q 12 Hours
◯ QD PRN
◯ BID PRN
◯ TID PRN
◯ QID PRN
◯ Q 1 Hour PRN
◯ Q 2 Hours PRN
◯ Q 3 Hours PRN
◯ Q 4 Hours PRN
◯ Q 4-6 Hours PRN
◯ Q 6 Hours PRN
◯ Q 8 Hours PRN
◯ Q 12 Hours PRN
◯ Other
◯ Unknown/ Unsure
◯ Refused/ No Response

(7) If frequency is "other", please describe:

____

(7) Indication	◯ Anti-Inflammatory<br>◯ Constipation<br>◯ Depression/Anxiety<br>◯ Headache<br>◯ Low Iron<br>◯ Pain<br>◯ Vitamins<br>◯ Allergies<br>◯ Hypertension<br>◯ Birth Control<br>◯ Lactation Support<br>◯ Mastitis<br>◯ Influenza<br>◯ Other<br>◯ Unknown/ Unsure<br>◯ No Response/ Refused
(7) If indication is "other", please describe:	__________________________
Maternal Medication 8	
(8) Medication Name	◯ DHA<br>◯ Docusate sodium<br>◯ Ibuprofen<br>◯ Iron<br>◯ Miralax<br>◯ Oxycodone- acetaminophen (percocet)<br>◯ Prenatal vitamins<br>◯ Prenatal vitamins + DHA<br>◯ Tylenol<br>◯ Vitamin D<br>◯ Antimicrobial<br>◯ Other vitamin<br>◯ Birth control pills<br>◯ Antihistamine<br>◯ Herbal Supplement<br>◯ Other<br>◯ Unsure/ Unknown<br>◯ Refused/ No Response
(8) If medication is "antimicrobial" please describe	◯ Acyclovir<br>◯ Amoxicillin<br>◯ Ancef/ Cefazolin<br>◯ Azithromycin<br>◯ Clindamycin<br>◯ Diflucan/ Fluconazole<br>◯ Flagyl<br>◯ Macrobid<br>◯ Penicillin G<br>◯ Vancomycin<br>◯ Oseltamivir/Tamiflu<br>◯ Other<br>◯ Unsure/ Unknown<br>◯ No Response/ Refused
(8) If medication is "other", please describe:	__________________________
(8) Dosage/Strength	__________________________

(8) Units	◯ Capsule<br>◯ Cup<br>◯ Drop<br>◯ Gram<br>◯ Kilogram<br>◯ Liter<br>◯ Microgram<br>◯ Milligram<br>◯ Milliliter<br>◯ Ounce<br>◯ Pint<br>◯ Puff<br>◯ Quart<br>◯ Tablespoon<br>◯ Teaspoon<br>◯ Unit<br>◯ Other<br>◯ Unknowns/ Unsure<br>◯ No Response/ Refused
(8) If units is "other", please describe:	__________________________________
(8) Route	◯ Buccal<br>◯ Enteral<br>◯ Intradermal<br>◯ Intramuscular<br>◯ Intranasal<br>◯ Intravenous<br>◯ Ophthalmic<br>◯ Oral<br>◯ Otic<br>◯ Rectal<br>◯ Subcutaneous<br>◯ Sublingual<br>◯ Topical<br>◯ Transdermal<br>◯ Vaginally<br>◯ Other<br>◯ Unknown/ Unsure<br>◯ No Response/ Refused
(8) If route is "other", please describe:	__________________________________

(8) Frequency	◯ QD<br>◯ PRN<br>◯ BID<br>◯ TID<br>◯ QID<br>◯ Q 1 Hour<br>◯ Q 2 Hours<br>◯ Q 3 Hours<br>◯ Q 4 Hours<br>◯ Q 4-6 Hours<br>◯ Q 6 Hours<br>◯ Q 8 Hours<br>◯ Q 12 Hours<br>◯ QD PRN<br>◯ BID PRN<br>◯ TID PRN<br>◯ QID PRN<br>◯ Q 1 Hour PRN<br>◯ Q 2 Hours PRN<br>◯ Q 3 Hours PRN<br>◯ Q 4 Hours PRN<br>◯ Q 4-6 Hours PRN<br>◯ Q 6 Hours PRN<br>◯ Q 8 Hours PRN<br>◯ Q 12 Hours PRN<br>◯ Other<br>◯ Unknown/ Unsure<br>◯ Refused/ No Response
(8) If frequency is "other", please describe:	__________________________
(8) Indication	◯ Anti-Inflammatory<br>◯ Constipation<br>◯ Depression/Anxiety<br>◯ Headache<br>◯ Low Iron<br>◯ Pain<br>◯ Vitamins<br>◯ Allergies<br>◯ Hypertension<br>◯ Birth Control<br>◯ Lactation Support<br>◯ Mastitis<br>◯ Influenza<br>◯ Other<br>◯ Unknown/ Unsure<br>◯ No Response/ Refused
(8) If indication is "other", please describe:	__________________________
Maternal Medication 9	

(9) Medication Name	◯ DHA<br>◯ Docusate sodium<br>◯ Ibuprofen<br>◯ Iron<br>◯ Miralax<br>◯ Oxycodone- acetaminophen (percocet)<br>◯ Prenatal vitamins<br>◯ Prenatal vitamins + DHA<br>◯ Tylenol<br>◯ Vitamin D<br>◯ Antimicrobial<br>◯ Other vitamin<br>◯ Birth control pills<br>◯ Antihistamine<br>◯ Herbal Supplement<br>◯ Other<br>◯ Unsure/ Unknown<br>◯ Refused/ No Response
(9) If medication is "antimicrobial" please describe	◯ Acyclovir<br>◯ Amoxicillin<br>◯ Ancef/ Cefazolin<br>◯ Azithromycin<br>◯ Clindamycin<br>◯ Diflucan/ Fluconazole<br>◯ Flagyl<br>◯ Macrobid<br>◯ Penicillin G<br>◯ Vancomycin<br>◯ Oseltamivir/Tamiflu<br>◯ Other<br>◯ Unsure/ Unknown<br>◯ No Response/ Refused
(9) If medication is "other", please describe:	________________________________
(9) Dosage/Strength	________________________________
(9) Units	◯ Capsule<br>◯ Cup<br>◯ Drop<br>◯ Gram<br>◯ Kilogram<br>◯ Liter<br>◯ Microgram<br>◯ Milligram<br>◯ Milliliter<br>◯ Ounce<br>◯ Pint<br>◯ Puff<br>◯ Quart<br>◯ Tablespoon<br>◯ Teaspoon<br>◯ Unit<br>◯ Other<br>◯ Unknowns/ Unsure<br>◯ No Response/ Refused
(9) If units is "other", please describe:	________________________________

(9) Route	◯ Buccal<br>◯ Enteral<br>◯ Intradermal<br>◯ Intramuscular<br>◯ Intranasal<br>◯ Intravenous<br>◯ Ophthalmic<br>◯ Oral<br>◯ Otic<br>◯ Rectal<br>◯ Subcutaneous<br>◯ Sublingual<br>◯ Topical<br>◯ Transdermal<br>◯ Vaginally<br>◯ Other<br>◯ Unknown/ Unsure<br>◯ No Response/ Refused
(9) If route is "other", please describe:	__________________________________
(9) Frequency	◯ QD<br>◯ PRN<br>◯ BID<br>◯ TID<br>◯ QID<br>◯ Q 1 Hour<br>◯ Q 2 Hours<br>◯ Q 3 Hours<br>◯ Q 4 Hours<br>◯ Q 4-6 Hours<br>◯ Q 6 Hours<br>◯ Q 8 Hours<br>◯ Q 12 Hours<br>◯ QD PRN<br>◯ BID PRN<br>◯ TID PRN<br>◯ QID PRN<br>◯ Q 1 Hour PRN<br>◯ Q 2 Hours PRN<br>◯ Q 3 Hours PRN<br>◯ Q 4 Hours PRN<br>◯ Q 4-6 Hours PRN<br>◯ Q 6 Hours PRN<br>◯ Q 8 Hours PRN<br>◯ Q 12 Hours PRN<br>◯ Other<br>◯ Unknown/ Unsure<br>◯ Refused/ No Response
(9) If frequency is "other", please describe:	__________________________________

(9) Indication	◯ Anti-Inflammatory<br>◯ Constipation<br>◯ Depression/Anxiety<br>◯ Headache<br>◯ Low Iron<br>◯ Pain<br>◯ Vitamins<br>◯ Allergies<br>◯ Hypertension<br>◯ Birth Control<br>◯ Lactation Support<br>◯ Mastitis<br>◯ Influenza<br>◯ Other<br>◯ Unknown/ Unsure<br>◯ No Response/ Refused
(9) If indication is "other", please describe:	__________________________
Maternal Medication 10	
(10) Medication Name	◯ DHA<br>◯ Docusate sodium<br>◯ Ibuprofen<br>◯ Iron<br>◯ Miralax<br>◯ Oxycodone- acetaminophen (percocet)<br>◯ Prenatal vitamins<br>◯ Prenatal vitamins + DHA<br>◯ Tylenol<br>◯ Vitamin D<br>◯ Antimicrobial<br>◯ Other vitamin<br>◯ Birth control pills<br>◯ Antihistamine<br>◯ Herbal Supplement<br>◯ Other<br>◯ Unsure/ Unknown<br>◯ Refused/ No Response
(10) If medication is "antimicrobial" please describe	◯ Acyclovir<br>◯ Amoxicillin<br>◯ Ancef/ Cefazolin<br>◯ Azithromycin<br>◯ Clindamycin<br>◯ Diflucan/ Fluconazole<br>◯ Flagyl<br>◯ Macrobid<br>◯ Penicillin G<br>◯ Vancomycin<br>◯ Oseltamivir/Tamiflu<br>◯ Other<br>◯ Unsure/ Unknown<br>◯ No Response/ Refused
(10) If medication is "other", please describe:	__________________________
(10) Dosage/Strength	__________________________

(10) Units

◯ Capsule
◯ Cup
◯ Drop
◯ Gram
◯ Kilogram
◯ Liter
◯ Microgram
◯ Milligram
◯ Milliliter
◯ Ounce
◯ Pint
◯ Puff
◯ Quart
◯ Tablespoon
◯ Teaspoon
◯ Unit
◯ Other
◯ Unknowns/ Unsure
◯ No Response/ Refused

(10) If units is "other", please describe: __________________________________

(10) Route

◯ Buccal
◯ Enteral
◯ Intradermal
◯ Intramuscular
◯ Intranasal
◯ Intravenous
◯ Ophthalmic
◯ Oral
◯ Otic
◯ Rectal
◯ Subcutaneous
◯ Sublingual
◯ Topical
◯ Transdermal
◯ Vaginally
◯ Other
◯ Unknown/ Unsure
◯ No Response/ Refused

(10) If route is "other", please describe: __________________________________

(10) Frequency	◯ QD<br>◯ PRN<br>◯ BID<br>◯ TID<br>◯ QID<br>◯ Q 1 Hour<br>◯ Q 2 Hours<br>◯ Q 3 Hours<br>◯ Q 4 Hours<br>◯ Q 4-6 Hours<br>◯ Q 6 Hours<br>◯ Q 8 Hours<br>◯ Q 12 Hours<br>◯ QD PRN<br>◯ BID PRN<br>◯ TID PRN<br>◯ QID PRN<br>◯ Q 1 Hour PRN<br>◯ Q 2 Hours PRN<br>◯ Q 3 Hours PRN<br>◯ Q 4 Hours PRN<br>◯ Q 4-6 Hours PRN<br>◯ Q 6 Hours PRN<br>◯ Q 8 Hours PRN<br>◯ Q 12 Hours PRN<br>◯ Other<br>◯ Unknown/ Unsure<br>◯ Refused/ No Response
(10) If frequency is "other", please describe:	______________________________
(10) Indication	◯ Anti-Inflammatory<br>◯ Constipation<br>◯ Depression/Anxiety<br>◯ Headache<br>◯ Low Iron<br>◯ Pain<br>◯ Vitamins<br>◯ Allergies<br>◯ Hypertension<br>◯ Birth Control<br>◯ Lactation Support<br>◯ Mastitis<br>◯ Influenza<br>◯ Other<br>◯ Unknown/ Unsure<br>◯ No Response/ Refused
(10) If indication is "other", please describe:	______________________________
If the mother is taking more than 10 medications, please record the name, dosage, units, route, frequency and indications of those medications here:	_____________________________________

Please ask for the mother's permission to take a photo of the labels of any of her dietary supplements. Please indicate that these supplements belong to the mother.

Household Structure

Including yourself, how many people live in your household?

- ◯ 1
- ◯ 2
- ◯ 3
- ◯ 4
- ◯ 5
- ◯ 6
- ◯ 7
- ◯ More than 7 people
- ◯ Unsure/Unknown
- ◯ Refused/No Response

Please list all household members, including the mother and study infant:

Household Member 1

(1) Household member's relation to the child

- ◯ Study Infant
- ◯ Study Mother
- ◯ Father
- ◯ Sister
- ◯ Brother
- ◯ Aunt
- ◯ Uncle
- ◯ Cousin
- ◯ Maternal Grandmother
- ◯ Maternal Grandfather
- ◯ Paternal Grandmother
- ◯ Paternal Grandfather
- ◯ Maternal Great Grandmother
- ◯ Maternal Great Grandfather
- ◯ Paternal Great Grandmother
- ◯ Paternal Great Grandfather
- ◯ Family Friend
- ◯ Significant Other (study mother's significant other that is not the infant's father)
- ◯ Other
- ◯ Grandmother
- ◯ Grandfather
- ◯ Great Grandmother
- ◯ Great Grandfather

(1) If member is "other", please describe:

____

(1) Age

____

(1) Sex

- ◯ Male
- ◯ Female

Household Member 2

(2) Household member's relation to the child	◯ Study Infant<br>◯ Study Mother<br>◯ Father<br>◯ Sister<br>◯ Brother<br>◯ Aunt<br>◯ Uncle<br>◯ Cousin<br>◯ Maternal Grandmother<br>◯ Maternal Grandfather<br>◯ Paternal Grandmother<br>◯ Paternal Grandfather<br>◯ Maternal Great Grandmother<br>◯ Maternal Great Grandfather<br>◯ Paternal Great Grandmother<br>◯ Paternal Great Grandfather<br>◯ Family Friend<br>◯ Significant Other (study mother's significant other that is not the infant's father)<br>◯ Other<br>◯ Grandmother<br>◯ Grandfather<br>◯ Great Grandmother<br>◯ Great Grandfather
(2) If member is "other", please describe:	__________________________________
(2) Age	__________________________________
(2) Sex	◯ Male<br>◯ Female

Household Member 3

(3) Household member's relation to the child	◯ Study Infant<br>◯ Study Mother<br>◯ Father<br>◯ Sister<br>◯ Brother<br>◯ Aunt<br>◯ Uncle<br>◯ Cousin<br>◯ Maternal Grandmother<br>◯ Maternal Grandfather<br>◯ Paternal Grandmother<br>◯ Paternal Grandfather<br>◯ Maternal Great Grandmother<br>◯ Maternal Great Grandfather<br>◯ Paternal Great Grandmother<br>◯ Paternal Great Grandfather<br>◯ Family Friend<br>◯ Significant Other (study mother's significant other that is not the infant's father)<br>◯ Other<br>◯ Grandmother<br>◯ Grandfather<br>◯ Great Grandmother<br>◯ Great Grandfather
(3) If member is "other", please describe:	________________________
(3) Age	________________________
(3) Sex	◯ Male<br>◯ Female

Household Member 4

(4) Household member's relation to the child	◯ Study Infant<br>◯ Study Mother<br>◯ Father<br>◯ Sister<br>◯ Brother<br>◯ Aunt<br>◯ Uncle<br>◯ Cousin<br>◯ Maternal Grandmother<br>◯ Maternal Grandfather<br>◯ Paternal Grandmother<br>◯ Paternal Grandfather<br>◯ Maternal Great Grandmother<br>◯ Maternal Great Grandfather<br>◯ Paternal Great Grandmother<br>◯ Paternal Great Grandfather<br>◯ Family Friend<br>◯ Significant Other (study mother's significant other that is not the infant's father)<br>◯ Other<br>◯ Grandmother<br>◯ Grandfather<br>◯ Great Grandmother<br>◯ Great Grandfather
(4) If member is "other", please describe:	__________________________
(4) Age	__________________________
(4) Sex	◯ Male<br>◯ Female

Household Member 5

(5) Household member's relation to the child	◯ Study Infant<br>◯ Study Mother<br>◯ Father<br>◯ Sister<br>◯ Brother<br>◯ Aunt<br>◯ Uncle<br>◯ Cousin<br>◯ Maternal Grandmother<br>◯ Maternal Grandfather<br>◯ Paternal Grandmother<br>◯ Paternal Grandfather<br>◯ Maternal Great Grandmother<br>◯ Maternal Great Grandfather<br>◯ Paternal Great Grandmother<br>◯ Paternal Great Grandfather<br>◯ Family Friend<br>◯ Significant Other (study mother's significant other that is not the infant's father)<br>◯ Other<br>◯ Grandmother<br>◯ Grandfather<br>◯ Great Grandmother<br>◯ Great Grandfather
(5) If member is "other", please describe:	__________________________________
(5) Age	__________________________________
(5) Sex	◯ Male<br>◯ Female

Household Member 6

(6) Household member's relation to the child	◯ Study Infant<br>◯ Study Mother<br>◯ Father<br>◯ Sister<br>◯ Brother<br>◯ Aunt<br>◯ Uncle<br>◯ Cousin<br>◯ Maternal Grandmother<br>◯ Maternal Grandfather<br>◯ Paternal Grandmother<br>◯ Paternal Grandfather<br>◯ Maternal Great Grandmother<br>◯ Maternal Great Grandfather<br>◯ Paternal Great Grandmother<br>◯ Paternal Great Grandfather<br>◯ Family Friend<br>◯ Significant Other (study mother's significant other that is not the infant's father)<br>◯ Other<br>◯ Grandmother<br>◯ Grandfather<br>◯ Great Grandmother<br>◯ Great Grandfather
(6) If member is "other", please describe:	__________________________________
(6) Age	__________________________________
(6) Sex	◯ Male<br>◯ Female

Household Member 7

(7) Household member's relation to the child

- ◯ Study Infant
- ◯ Study Mother
- ◯ Father
- ◯ Sister
- ◯ Brother
- ◯ Aunt
- ◯ Uncle
- ◯ Cousin
- ◯ Maternal Grandmother
- ◯ Maternal Grandfather
- ◯ Paternal Grandmother
- ◯ Paternal Grandfather
- ◯ Maternal Great Grandmother
- ◯ Maternal Great Grandfather
- ◯ Paternal Great Grandmother
- ◯ Paternal Great Grandfather
- ◯ Family Friend
- ◯ Significant Other (study mother's significant other that is not the infant's father)
- ◯ Other
- ◯ Grandmother
- ◯ Grandfather
- ◯ Great Grandmother
- ◯ Great Grandfather

---

(7) If member is "other", please describe: ______________________

---

(7) Age ______________________

---

(7) Sex

- ◯ Male
- ◯ Female

If there are more than 7 people living in your household, please describe their relationship to the child, their age and their sex here: ______________________

(8) Household member's relation to child

- ◯ Study Infant
- ◯ Study Mother
- ◯ Father
- ◯ Sister
- ◯ Brother
- ◯ Aunt
- ◯ Uncle
- ◯ Cousin
- ◯ Maternal Grandmother
- ◯ Maternal Grandfather
- ◯ Paternal Grandmother
- ◯ Paternal Grandfather
- ◯ Maternal Great Grandmother
- ◯ Maternal Great Grandfather
- ◯ Paternal Great Grandmother
- ◯ Paternal Great Grandfather
- ◯ Family Friend
- ◯ Significant Other (study mother's significant other that is not the infant's father)
- ◯ Other
- ◯ Grandmother
- ◯ Grandfather
- ◯ Great Grandmother
- ◯ Great Grandfather

(9) Household member's relation to child

- ◯ Study Infant
- ◯ Study Mother
- ◯ Father
- ◯ Sister
- ◯ Brother
- ◯ Aunt
- ◯ Uncle
- ◯ Cousin
- ◯ Maternal Grandmother
- ◯ Maternal Grandfather
- ◯ Paternal Grandmother
- ◯ Paternal Grandfather
- ◯ Maternal Great Grandmother
- ◯ Maternal Great Grandfather
- ◯ Paternal Great Grandmother
- ◯ Paternal Great Grandfather
- ◯ Family Friend
- ◯ Significant Other (study mother's significant other that is not the infant's father)
- ◯ Other
- ◯ Grandmother
- ◯ Grandfather
- ◯ Great Grandmother
- ◯ Great Grandfather

(10) Household member's relation to child

- ◯ Study Infant
- ◯ Study Mother
- ◯ Father
- ◯ Sister
- ◯ Brother
- ◯ Aunt
- ◯ Uncle
- ◯ Cousin
- ◯ Maternal Grandmother
- ◯ Maternal Grandfather
- ◯ Paternal Grandmother
- ◯ Paternal Grandfather
- ◯ Maternal Great Grandmother
- ◯ Maternal Great Grandfather
- ◯ Paternal Great Grandmother
- ◯ Paternal Great Grandfather
- ◯ Family Friend
- ◯ Significant Other (study mother's significant other that is not the infant's father)
- ◯ Other
- ◯ Grandmother
- ◯ Grandfather
- ◯ Great Grandmother
- ◯ Great Grandfather

(11) Household member's relation to child

- ◯ Study Infant
- ◯ Study Mother
- ◯ Father
- ◯ Sister
- ◯ Brother
- ◯ Aunt
- ◯ Uncle
- ◯ Cousin
- ◯ Maternal Grandmother
- ◯ Maternal Grandfather
- ◯ Paternal Grandmother
- ◯ Paternal Grandfather
- ◯ Maternal Great Grandmother
- ◯ Maternal Great Grandfather
- ◯ Paternal Great Grandmother
- ◯ Paternal Great Grandfather
- ◯ Family Friend
- ◯ Significant Other (study mother's significant other that is not the infant's father)
- ◯ Other
- ◯ Grandmother
- ◯ Grandfather
- ◯ Great Grandmother
- ◯ Great Grandfather

Including the index child, total number of people that live in the household?

◯ 1
◯ 2
◯ 3
◯ 4
◯ 5
◯ 6
◯ 7
◯ 8
◯ 9
◯ 10
◯ 11
◯ 12
◯ 13
◯ 14
◯ 15
◯ 16
◯ 17
◯ 18
◯ 19
◯ 20
◯ 21
◯ 22
◯ 23
◯ 24
◯ 25
◯ 26
◯ 27
◯ 28
◯ 29
◯ 30
◯ 88 Unknown

Including the index child, total number of people < 5 years old	◯ 1<br>◯ 2<br>◯ 3<br>◯ 4<br>◯ 5<br>◯ 6<br>◯ 7<br>◯ 8<br>◯ 9<br>◯ 10<br>◯ 11<br>◯ 12<br>◯ 13<br>◯ 14<br>◯ 15<br>◯ 16<br>◯ 17<br>◯ 18<br>◯ 19<br>◯ 20<br>◯ 21<br>◯ 22<br>◯ 23<br>◯ 24<br>◯ 25<br>◯ 26<br>◯ 27<br>◯ 28<br>◯ 29<br>◯ 30<br>◯ 88 Unknown

Total number of people 5-17 years old	◯ 0<br>◯ 1<br>◯ 2<br>◯ 3<br>◯ 4<br>◯ 5<br>◯ 6<br>◯ 7<br>◯ 8<br>◯ 9<br>◯ 10<br>◯ 11<br>◯ 12<br>◯ 13<br>◯ 14<br>◯ 15<br>◯ 16<br>◯ 17<br>◯ 18<br>◯ 19<br>◯ 20<br>◯ 21<br>◯ 22<br>◯ 23<br>◯ 24<br>◯ 25<br>◯ 26<br>◯ 27<br>◯ 28<br>◯ 29<br>◯ 30<br>◯ Unknown

Including study mother, total number of people 18-64 years old	◯ 1<br>◯ 2<br>◯ 3<br>◯ 4<br>◯ 5<br>◯ 6<br>◯ 7<br>◯ 8<br>◯ 9<br>◯ 10<br>◯ 11<br>◯ 12<br>◯ 13<br>◯ 14<br>◯ 15<br>◯ 16<br>◯ 17<br>◯ 18<br>◯ 19<br>◯ 20<br>◯ 21<br>◯ 22<br>◯ 23<br>◯ 24<br>◯ 25<br>◯ 26<br>◯ 27<br>◯ 28<br>◯ 29<br>◯ 30<br>◯ 88 Unknown

Total number of people 65 years old and older

- ◯ 0
- ◯ 1
- ◯ 2
- ◯ 3
- ◯ 4
- ◯ 5
- ◯ 6
- ◯ 7
- ◯ 8
- ◯ 9
- ◯ 10
- ◯ 11
- ◯ 12
- ◯ 13
- ◯ 14
- ◯ 15
- ◯ 16
- ◯ 17
- ◯ 18
- ◯ 19
- ◯ 20
- ◯ 21
- ◯ 22
- ◯ 23
- ◯ 24
- ◯ 25
- ◯ 26
- ◯ 27
- ◯ 28
- ◯ 29
- ◯ 30
- ◯ Unknown

Childcare

Since [week_0_arm_1][nbcrf_inf_name] was born, have you worked outside the home?

- ◯ No
- ◯ Yes
- ◯ Unsure/Unknown
- ◯ Refused/No Response

If yes, how many days have you worked outside the home since [week_0_arm_1][nbcrf_inf_name] was born?

____

Who cares for [week_0_arm_1][nbcrf_inf_name]throughout the day?

Check all that apply

- ☐ Mother
- ☐ Child's Father
- ☐ Adult relative other than the father
- ☐ Unrelated adult, including licensed caregivers
- ☐ Children under age 18

If an adult relative, other than the father, cares for [week_0_arm_1][nbcrf_inf_name] throughout the day, please specify the relation to [week_0_arm_1][nbcrf_inf_name]:

____

If a child under age 18 cares for [week_0_arm_1][nbcrf_inf_name] throughout the day, please specify the relation to [week_0_arm_1][nbcrf_inf_name]:

____

Where does the care of [week_0_arm_1][nbcrf_inf_name] occur?

- ◯ Solely in my home
- ◯ Out of home child care at least once a week

If out of home child care, which is used most often:
◯ In someone else's home
◯ In a child care center/Headstart/Preschool

How many other children are in care in [week_0_arm_1][nbcrf_inf_name]'s group?
____

How many days per week is [week_0_arm_1][nbcrf_inf_name] in care outside of the home?
____

Between now and the time we see you next, at your child's 6 week clinic visit, do you plan to work outside the home?
◯ Yes
◯ No

Between now and the 6 week clinic visit, will you continue your current child care arrangements or change them?
◯ I plan to continue with my current arrangements
◯ I plan to change my current arrangements

Please briefly describe the change that you expect:
____

Infant Sleep Habits

We will now ask you questions about how and where your baby has slept since they were born

In which positions do you lay your baby down to sleep?

	Never	Rarely	Sometimes	Often	Always
On his or her side	◯	◯	◯	◯	◯
On his or her back	◯	◯	◯	◯	◯
On his or her stomach	◯	◯	◯	◯	◯

In the past 2 weeks, how often has [week_0_arm_1][nbcrf_inf_name] slept alone in his or her crib or bed?
◯ Never
◯ Rarely
◯ Sometimes
◯ Often
◯ Always

Who does [week_0_arm_1][nbcrf_inf_name] usually sleep with when he or she is not sleeping alone?
☐ Me
☐ My Husband or Partner
☐ Someone else
☐ Not applicable (My child always sleeps alone)

If [week_0_arm_1][nbcrf_inf_name] sleeps with someone else, please tell us who:
____

When [week_0_arm_1][nbcrf_inf_name] sleeps alone, is his or her crib in the same room where you sleep?
◯ No
◯ Yes
◯ Not applicable (my child never sleeps alone)

In the past two weeks, where has [week_0_arm_1][nbcrf_inf_name] USUALLY slept?<br><br>(Select one)	◯ In a crib, bassinet, or pack and play<br>◯ On a twin or larger mattress or bed ?<br>◯ On a couch, sofa, or armchair<br>◯ In an infant car seat or swing<br>◯ In a sleeping sack or wearable blanket<br>◯ With a blanket<br>◯ With toys, cushions, or pillows, including nursing pillows<br>◯ With crib bumper pads (mesh or non-mesh)
In the past two weeks, has [week_0_arm_1][nbcrf_inf_name] EVER slept in any of the locations listed below?<br><br>(Select all that apply)	☐ In a crib, bassinet, or pack and play<br>☐ On a twin or larger mattress or bed ?<br>☐ On a couch, sofa, or armchair<br>☐ In an infant car seat or swing<br>☐ In a sleeping sack or wearable blanket<br>☐ With a blanket<br>☐ With toys, cushions, or pillows, including nursing pillows<br>☐ With crib bumper pads (mesh or non-mesh)

Did a doctor, nurse or other health care worker tell you any of the following?

For each item, check "No" if they did not tell you or "Yes" if they did tell you.

	No	Yes
Place my baby on his or her back to sleep	◯	◯
Place my baby to sleep in a crib, bassinet or pack and play	◯	◯
Place my baby's crib or bed in my room	◯	◯
What things should and should not go in bed with my baby	◯	◯

Infant Race/Ethnicity

Is your child of Hispanic, Latina or Spanish origin?	◯ No<br>◯ Yes<br>◯ Unknown/ Unsure<br>◯ Refused/ No Response
How would you classify your child? (check all that apply)	☐ White<br>☐ Black, African American<br>☐ American Indian/ Alaskan Native<br>☐ Asian<br>☐ Native Hawaiian/ Pacific Islander<br>☐ None<br>☐ Unsure/ Unknown<br>☐ Refused/ No Response

Infant Health

What is the name of your child's pediatrician and/or the name of the practice?

____

Name of pediatric practice

____

Name of physician

(if non-specific, enter "rotating")

____

Since [week_0_arm_1][nbcrf_inf_name]'s birth, how many times has he or she been to the doctor's office, clinic or hospital for any reason?

- ◯ 0
- ◯ 1
- ◯ 2
- ◯ 3
- ◯ 4 or more times

Medical Visit #1

(1) Date of visit

____

(1) Date Description:

- ◯ Exact
- ◯ Approximate
- ◯ Unknown
- ◯ No Response/Refused

(1) Location

- ◯ Doctor's Office
- ◯ Clinic
- ◯ ED/Hospital

(1) Reason for visit

- ◯ Wellness/ Newborn Check-up
- ◯ Lactation Consultation
- ◯ Other
- ◯ Unknown/ Not Sure
- ◯ No Response/ Refused

(1) If reason is "other", please describe:

____

Medical Visit #2

(2) Date of visit

____

(2) Date Description:

- ◯ Exact
- ◯ Approximate
- ◯ Unknown
- ◯ No Response/Refused

(2) Location

- ◯ Doctor's Office
- ◯ Clinic
- ◯ ED/Hospital

(2) Reason for visit	◯ Wellness/ Newborn Check-up<br>◯ Lactation Consultation<br>◯ Other<br>◯ Unknown/ Not Sure<br>◯ No Response/ Refused
(2) If reason is "other", please describe:	__________________________
Medical Visit #3	
(3) Date of visit	__________________________
(3) Date Description:	◯ Exact<br>◯ Approximate<br>◯ Unknown<br>◯ No Response/Refused
(3) Location	◯ Doctor's Office<br>◯ Clinic<br>◯ ED/Hospital
(3) Reason for visit	◯ Wellness/ Newborn Check-up<br>◯ Lactation Consultation<br>◯ Other<br>◯ Unknown/ Not Sure<br>◯ No Response/ Refused
(3) If reason is "other", please describe:	__________________________
If [week_0_arm_1][nbcrf_inf_name] has had 4 or more medical visits since their birth, please enter the date, date description, location and reason for visit here:	________________________________
Is [week_0_arm_1][nbcrf_inf_name] currently ill?	◯ No<br>◯ Yes<br>◯ Unsure/Unknown<br>◯ Refused/ No Response
What is [week_0_arm_1][nbcrf_inf_name]'s current health problem?	________________________________
On average, how many bowel movements does [week_0_arm_1][nbcrf_inf_name] typically have each day?	__________________________<br>(record a number, not a range)

Infant Medication Use

Types of Medications

Vitamins or dietary supplements	◯ No<br>◯ Yes<br>◯ Unsure/Unknown<br>◯ Refused/No Response
Over the counter medications	◯ No<br>◯ Yes<br>◯ Unsure/Unknown<br>◯ Refused/No Response
Herbal or folk remedies	◯ No<br>◯ Yes<br>◯ Unsure/Unknown<br>◯ Refused/No Response
Prescription medications	◯ No<br>◯ Yes<br>◯ Unsure/Unknown<br>◯ Refused/No Response

Infant Medication List

How many vitamins/dietary supplements, over the counter medications, herbal/folk remedies or prescription medications is the infant taking in total?	◯ 1<br>◯ 2<br>◯ 3<br>◯ 4<br>◯ 5<br>◯ 6<br>◯ 7<br>◯ 8<br>◯ 9<br>◯ 10<br>◯ 11 or More

Infant Medication 1

(1) Medication Name	◯ Erythromycin eye cream<br>◯ Gripe water<br>◯ Multivitamin<br>◯ Nystatin<br>◯ Simethicone<br>◯ Tri-Vi-Sol<br>◯ Vitamin D<br>◯ White petrolatum<br>◯ Zinc oxide cream<br>◯ Other<br>◯ Unsure/ Unknown<br>◯ No Response/ Refused
(1) If medication is "other", please describe:	__________________________________
(1) Dosage/Strength	__________________________________

(1) Units	◯ Capsule<br>◯ Cup<br>◯ Drop<br>◯ Gram<br>◯ Kilogram<br>◯ Liter<br>◯ Microgram<br>◯ Milligram<br>◯ Milliliter<br>◯ Ounce<br>◯ Pint<br>◯ Puff<br>◯ Quart<br>◯ Tablespoon<br>◯ Teaspoon<br>◯ Unit<br>◯ Other<br>◯ Unknowns/ Unsure<br>◯ No Response/ Refused
(1) If units is "other", please describe:	______________________________
(1) Route	◯ Buccal<br>◯ Enteral<br>◯ Intradermal<br>◯ Intramuscular<br>◯ Intranasal<br>◯ Intravenous<br>◯ Ophthalmic<br>◯ Oral<br>◯ Otic<br>◯ Rectal<br>◯ Subcutaneous<br>◯ Sublingual<br>◯ Topical<br>◯ Transdermal<br>◯ Vaginally<br>◯ Other<br>◯ Unknown/ Unsure<br>◯ No Response/ Refused
(1) If route is "other", please describe:	______________________________

(1) Frequency	◯ QD<br>◯ PRN<br>◯ BID<br>◯ TID<br>◯ QID<br>◯ Q 1 Hour<br>◯ Q 2 Hours<br>◯ Q 3 Hours<br>◯ Q 4 Hours<br>◯ Q 4-6 Hours<br>◯ Q 6 Hours<br>◯ Q 8 Hours<br>◯ Q 12 Hours<br>◯ QD PRN<br>◯ BID PRN<br>◯ TID PRN<br>◯ QID PRN<br>◯ Q 1 Hour PRN<br>◯ Q 2 Hours PRN<br>◯ Q 3 Hours PRN<br>◯ Q 4 Hours PRN<br>◯ Q 4-6 Hours PRN<br>◯ Q 6 Hours PRN<br>◯ Q 8 Hours PRN<br>◯ Q 12 Hours PRN<br>◯ Other<br>◯ Unknown/ Unsure<br>◯ Refused/ No Response
(1) If frequency is "other", please describe:	__________________________________
(1) Indication	◯ Diaper Rash<br>◯ Fussy<br>◯ Oral Thrush<br>◯ Other<br>◯ Unsure/ Unknown<br>◯ Refused/ No Response
(1) If indication is "other", please describe:	__________________________________
Infant Medication 2	
(2) Medication Name	◯ Erythromycin eye cream<br>◯ Gripe water<br>◯ Multivitamin<br>◯ Nystatin<br>◯ Simethicone<br>◯ Tri-Vi-Sol<br>◯ Vitamin D<br>◯ White petrolatum<br>◯ Zinc oxide cream<br>◯ Other<br>◯ Unsure/ Unknown<br>◯ No Response/ Refused
(2) If medication is "other", please describe:	__________________________________

(2) Dosage/Strength

____

(2) Units

- ◯ Capsule
- ◯ Cup
- ◯ Drop
- ◯ Gram
- ◯ Kilogram
- ◯ Liter
- ◯ Microgram
- ◯ Milligram
- ◯ Milliliter
- ◯ Ounce
- ◯ Pint
- ◯ Puff
- ◯ Quart
- ◯ Tablespoon
- ◯ Teaspoon
- ◯ Unit
- ◯ Other
- ◯ Unknowns/ Unsure
- ◯ No Response/ Refused

(2) If units is "other", please describe:

____

(2) Route

- ◯ Buccal
- ◯ Enteral
- ◯ Intradermal
- ◯ Intramuscular
- ◯ Intranasal
- ◯ Intravenous
- ◯ Ophthalmic
- ◯ Oral
- ◯ Otic
- ◯ Rectal
- ◯ Subcutaneous
- ◯ Sublingual
- ◯ Topical
- ◯ Transdermal
- ◯ Vaginally
- ◯ Other
- ◯ Unknown/ Unsure
- ◯ No Response/ Refused

(2) If route is "other", please describe:

____

(2) Frequency	◯ QD<br>◯ PRN<br>◯ BID<br>◯ TID<br>◯ QID<br>◯ Q 1 Hour<br>◯ Q 2 Hours<br>◯ Q 3 Hours<br>◯ Q 4 Hours<br>◯ Q 4-6 Hours<br>◯ Q 6 Hours<br>◯ Q 8 Hours<br>◯ Q 12 Hours<br>◯ QD PRN<br>◯ BID PRN<br>◯ TID PRN<br>◯ QID PRN<br>◯ Q 1 Hour PRN<br>◯ Q 2 Hours PRN<br>◯ Q 3 Hours PRN<br>◯ Q 4 Hours PRN<br>◯ Q 4-6 Hours PRN<br>◯ Q 6 Hours PRN<br>◯ Q 8 Hours PRN<br>◯ Q 12 Hours PRN<br>◯ Other<br>◯ Unknown/ Unsure<br>◯ Refused/ No Response
(2) If frequency is "other", please describe:	__________________________________
(2) Indication	◯ Diaper Rash<br>◯ Fussy<br>◯ Oral Thrush<br>◯ Other<br>◯ Unsure/ Unknown<br>◯ Refused/ No Response
(2) If indication is "other", please describe:	__________________________________
Infant Medication 3	
(3) Medication Name	◯ Erythromycin eye cream<br>◯ Gripe water<br>◯ Multivitamin<br>◯ Nystatin<br>◯ Simethicone<br>◯ Tri-Vi-Sol<br>◯ Vitamin D<br>◯ White petrolatum<br>◯ Zinc oxide cream<br>◯ Other<br>◯ Unsure/ Unknown<br>◯ No Response/ Refused
(3) If medication is "other", please describe:	__________________________________

(3) Dosage/Strength

____

(3) Units
◯ Capsule
◯ Cup
◯ Drop
◯ Gram
◯ Kilogram
◯ Liter
◯ Microgram
◯ Milligram
◯ Milliliter
◯ Ounce
◯ Pint
◯ Puff
◯ Quart
◯ Tablespoon
◯ Teaspoon
◯ Unit
◯ Other
◯ Unknowns/ Unsure
◯ No Response/ Refused

(3) If units is "other", please describe:

____

(3) Route
◯ Buccal
◯ Enteral
◯ Intradermal
◯ Intramuscular
◯ Intranasal
◯ Intravenous
◯ Ophthalmic
◯ Oral
◯ Otic
◯ Rectal
◯ Subcutaneous
◯ Sublingual
◯ Topical
◯ Transdermal
◯ Vaginally
◯ Other
◯ Unknown/ Unsure
◯ No Response/ Refused

(3) If route is "other", please describe:

____

(3) Frequency	◯ QD<br>◯ PRN<br>◯ BID<br>◯ TID<br>◯ QID<br>◯ Q 1 Hour<br>◯ Q 2 Hours<br>◯ Q 3 Hours<br>◯ Q 4 Hours<br>◯ Q 4-6 Hours<br>◯ Q 6 Hours<br>◯ Q 8 Hours<br>◯ Q 12 Hours<br>◯ QD PRN<br>◯ BID PRN<br>◯ TID PRN<br>◯ QID PRN<br>◯ Q 1 Hour PRN<br>◯ Q 2 Hours PRN<br>◯ Q 3 Hours PRN<br>◯ Q 4 Hours PRN<br>◯ Q 4-6 Hours PRN<br>◯ Q 6 Hours PRN<br>◯ Q 8 Hours PRN<br>◯ Q 12 Hours PRN<br>◯ Other<br>◯ Unknown/ Unsure<br>◯ Refused/ No Response
(3) If frequency is "other", please describe:	__________________________________
(3) Indication	◯ Diaper Rash<br>◯ Fussy<br>◯ Oral Thrush<br>◯ Other<br>◯ Unsure/ Unknown<br>◯ Refused/ No Response
(3) If indication is "other", please describe:	__________________________________
Infant Medication 4	
(4) Medication Name	◯ Erythromycin eye cream<br>◯ Gripe water<br>◯ Multivitamin<br>◯ Nystatin<br>◯ Simethicone<br>◯ Tri-Vi-Sol<br>◯ Vitamin D<br>◯ White petrolatum<br>◯ Zinc oxide cream<br>◯ Other<br>◯ Unsure/ Unknown<br>◯ No Response/ Refused
(4) If medication is "other", please describe:	__________________________________

(4) Dosage/Strength

____

(4) Units

◯ Capsule
◯ Cup
◯ Drop
◯ Gram
◯ Kilogram
◯ Liter
◯ Microgram
◯ Milligram
◯ Milliliter
◯ Ounce
◯ Pint
◯ Puff
◯ Quart
◯ Tablespoon
◯ Teaspoon
◯ Unit
◯ Other
◯ Unknowns/ Unsure
◯ No Response/ Refused

(4) If units is "other", please describe:

____

(4) Route

◯ Buccal
◯ Enteral
◯ Intradermal
◯ Intramuscular
◯ Intranasal
◯ Intravenous
◯ Ophthalmic
◯ Oral
◯ Otic
◯ Rectal
◯ Subcutaneous
◯ Sublingual
◯ Topical
◯ Transdermal
◯ Vaginally
◯ Other
◯ Unknown/ Unsure
◯ No Response/ Refused

(4) If route is "other", please describe:

____

(4) Frequency	◯ QD<br>◯ PRN<br>◯ BID<br>◯ TID<br>◯ QID<br>◯ Q 1 Hour<br>◯ Q 2 Hours<br>◯ Q 3 Hours<br>◯ Q 4 Hours<br>◯ Q 4-6 Hours<br>◯ Q 6 Hours<br>◯ Q 8 Hours<br>◯ Q 12 Hours<br>◯ QD PRN<br>◯ BID PRN<br>◯ TID PRN<br>◯ QID PRN<br>◯ Q 1 Hour PRN<br>◯ Q 2 Hours PRN<br>◯ Q 3 Hours PRN<br>◯ Q 4 Hours PRN<br>◯ Q 4-6 Hours PRN<br>◯ Q 6 Hours PRN<br>◯ Q 8 Hours PRN<br>◯ Q 12 Hours PRN<br>◯ Other<br>◯ Unknown/ Unsure<br>◯ Refused/ No Response
(4) If frequency is "other", please describe:	________________________________
(4) Indication	◯ Diaper Rash<br>◯ Fussy<br>◯ Oral Thrush<br>◯ Other<br>◯ Unsure/ Unknown<br>◯ Refused/ No Response
(4) If indication is "other", please describe:	________________________________
Infant Medication 5	
(5) Medication Name	◯ Erythromycin eye cream<br>◯ Gripe water<br>◯ Multivitamin<br>◯ Nystatin<br>◯ Simethicone<br>◯ Tri-Vi-Sol<br>◯ Vitamin D<br>◯ White petrolatum<br>◯ Zinc oxide cream<br>◯ Other<br>◯ Unsure/ Unknown<br>◯ No Response/ Refused
(5) If medication is "other", please describe:	________________________________

(5) Dosage/Strength

____

(5) Units

- ◯ Capsule
- ◯ Cup
- ◯ Drop
- ◯ Gram
- ◯ Kilogram
- ◯ Liter
- ◯ Microgram
- ◯ Milligram
- ◯ Milliliter
- ◯ Ounce
- ◯ Pint
- ◯ Puff
- ◯ Quart
- ◯ Tablespoon
- ◯ Teaspoon
- ◯ Unit
- ◯ Other
- ◯ Unknowns/ Unsure
- ◯ No Response/ Refused

(5) If units is "other", please describe:

____

(5) Route

- ◯ Buccal
- ◯ Enteral
- ◯ Intradermal
- ◯ Intramuscular
- ◯ Intranasal
- ◯ Intravenous
- ◯ Ophthalmic
- ◯ Oral
- ◯ Otic
- ◯ Rectal
- ◯ Subcutaneous
- ◯ Sublingual
- ◯ Topical
- ◯ Transdermal
- ◯ Vaginally
- ◯ Other
- ◯ Unknown/ Unsure
- ◯ No Response/ Refused

(5) If route is "other", please describe:

____

(5) Frequency	◯ QD<br>◯ PRN<br>◯ BID<br>◯ TID<br>◯ QID<br>◯ Q 1 Hour<br>◯ Q 2 Hours<br>◯ Q 3 Hours<br>◯ Q 4 Hours<br>◯ Q 4-6 Hours<br>◯ Q 6 Hours<br>◯ Q 8 Hours<br>◯ Q 12 Hours<br>◯ QD PRN<br>◯ BID PRN<br>◯ TID PRN<br>◯ QID PRN<br>◯ Q 1 Hour PRN<br>◯ Q 2 Hours PRN<br>◯ Q 3 Hours PRN<br>◯ Q 4 Hours PRN<br>◯ Q 4-6 Hours PRN<br>◯ Q 6 Hours PRN<br>◯ Q 8 Hours PRN<br>◯ Q 12 Hours PRN<br>◯ Other<br>◯ Unknown/ Unsure<br>◯ Refused/ No Response
(5) If frequency is "other", please describe:	__________________________________
(5) Indication	◯ Diaper Rash<br>◯ Fussy<br>◯ Oral Thrush<br>◯ Other<br>◯ Unsure/ Unknown<br>◯ Refused/ No Response
(5) If indication is "other", please describe:	__________________________________
Infant Medication 6	
(6) Medication Name	◯ Erythromycin eye cream<br>◯ Gripe water<br>◯ Multivitamin<br>◯ Nystatin<br>◯ Simethicone<br>◯ Tri-Vi-Sol<br>◯ Vitamin D<br>◯ White petrolatum<br>◯ Zinc oxide cream<br>◯ Other<br>◯ Unsure/ Unknown<br>◯ No Response/ Refused
(6) If medication is "other", please describe:	__________________________________

(6) Dosage/Strength	__________________________
(6) Units	◯ Capsule<br>◯ Cup<br>◯ Drop<br>◯ Gram<br>◯ Kilogram<br>◯ Liter<br>◯ Microgram<br>◯ Milligram<br>◯ Milliliter<br>◯ Ounce<br>◯ Pint<br>◯ Puff<br>◯ Quart<br>◯ Tablespoon<br>◯ Teaspoon<br>◯ Unit<br>◯ Other<br>◯ Unknowns/ Unsure<br>◯ No Response/ Refused
(6) If units is "other", please describe:	__________________________
(6) Route	◯ Buccal<br>◯ Enteral<br>◯ Intradermal<br>◯ Intramuscular<br>◯ Intranasal<br>◯ Intravenous<br>◯ Ophthalmic<br>◯ Oral<br>◯ Otic<br>◯ Rectal<br>◯ Subcutaneous<br>◯ Sublingual<br>◯ Topical<br>◯ Transdermal<br>◯ Vaginally<br>◯ Other<br>◯ Unknown/ Unsure<br>◯ No Response/ Refused
(6) If route is "other", please describe:	__________________________

(6) Frequency	◯ QD<br>◯ PRN<br>◯ BID<br>◯ TID<br>◯ QID<br>◯ Q 1 Hour<br>◯ Q 2 Hours<br>◯ Q 3 Hours<br>◯ Q 4 Hours<br>◯ Q 4-6 Hours<br>◯ Q 6 Hours<br>◯ Q 8 Hours<br>◯ Q 12 Hours<br>◯ QD PRN<br>◯ BID PRN<br>◯ TID PRN<br>◯ QID PRN<br>◯ Q 1 Hour PRN<br>◯ Q 2 Hours PRN<br>◯ Q 3 Hours PRN<br>◯ Q 4 Hours PRN<br>◯ Q 4-6 Hours PRN<br>◯ Q 6 Hours PRN<br>◯ Q 8 Hours PRN<br>◯ Q 12 Hours PRN<br>◯ Other<br>◯ Unknown/ Unsure<br>◯ Refused/ No Response
(6) If frequency is "other", please describe:	__________________________________
(6) Indication	◯ Diaper Rash<br>◯ Fussy<br>◯ Oral Thrush<br>◯ Other<br>◯ Unsure/ Unknown<br>◯ Refused/ No Response
(6) If indication is "other", please describe:	__________________________________
Infant Medication 7	
(7) Medication Name	◯ Erythromycin eye cream<br>◯ Gripe water<br>◯ Multivitamin<br>◯ Nystatin<br>◯ Simethicone<br>◯ Tri-Vi-Sol<br>◯ Vitamin D<br>◯ White petrolatum<br>◯ Zinc oxide cream<br>◯ Other<br>◯ Unsure/ Unknown<br>◯ No Response/ Refused
(7) If medication is "other", please describe:	__________________________________

(7) Dosage/Strength

____

(7) Units

- ◯ Capsule
- ◯ Cup
- ◯ Drop
- ◯ Gram
- ◯ Kilogram
- ◯ Liter
- ◯ Microgram
- ◯ Milligram
- ◯ Milliliter
- ◯ Ounce
- ◯ Pint
- ◯ Puff
- ◯ Quart
- ◯ Tablespoon
- ◯ Teaspoon
- ◯ Unit
- ◯ Other
- ◯ Unknowns/ Unsure
- ◯ No Response/ Refused

(7) If units is "other", please describe:

____

(7) Route

- ◯ Buccal
- ◯ Enteral
- ◯ Intradermal
- ◯ Intramuscular
- ◯ Intranasal
- ◯ Intravenous
- ◯ Ophthalmic
- ◯ Oral
- ◯ Otic
- ◯ Rectal
- ◯ Subcutaneous
- ◯ Sublingual
- ◯ Topical
- ◯ Transdermal
- ◯ Vaginally
- ◯ Other
- ◯ Unknown/ Unsure
- ◯ No Response/ Refused

(7) If route is "other", please describe:

____

(7) Frequency	◯ QD<br>◯ PRN<br>◯ BID<br>◯ TID<br>◯ QID<br>◯ Q 1 Hour<br>◯ Q 2 Hours<br>◯ Q 3 Hours<br>◯ Q 4 Hours<br>◯ Q 4-6 Hours<br>◯ Q 6 Hours<br>◯ Q 8 Hours<br>◯ Q 12 Hours<br>◯ QD PRN<br>◯ BID PRN<br>◯ TID PRN<br>◯ QID PRN<br>◯ Q 1 Hour PRN<br>◯ Q 2 Hours PRN<br>◯ Q 3 Hours PRN<br>◯ Q 4 Hours PRN<br>◯ Q 4-6 Hours PRN<br>◯ Q 6 Hours PRN<br>◯ Q 8 Hours PRN<br>◯ Q 12 Hours PRN<br>◯ Other<br>◯ Unknown/ Unsure<br>◯ Refused/ No Response
(7) If frequency is "other", please describe:	__________________________________
(7) Indication	◯ Diaper Rash<br>◯ Fussy<br>◯ Oral Thrush<br>◯ Other<br>◯ Unsure/ Unknown<br>◯ Refused/ No Response
(7) If indication is "other", please describe:	__________________________________
Infant Medication 8	
(8) Medication Name	◯ Erythromycin eye cream<br>◯ Gripe water<br>◯ Multivitamin<br>◯ Nystatin<br>◯ Simethicone<br>◯ Tri-Vi-Sol<br>◯ Vitamin D<br>◯ White petrolatum<br>◯ Zinc oxide cream<br>◯ Other<br>◯ Unsure/ Unknown<br>◯ No Response/ Refused
(8) If medication is "other", please describe:	__________________________________

(8) Dosage/Strength

____

(8) Units

- ◯ Capsule
- ◯ Cup
- ◯ Drop
- ◯ Gram
- ◯ Kilogram
- ◯ Liter
- ◯ Microgram
- ◯ Milligram
- ◯ Milliliter
- ◯ Ounce
- ◯ Pint
- ◯ Puff
- ◯ Quart
- ◯ Tablespoon
- ◯ Teaspoon
- ◯ Unit
- ◯ Other
- ◯ Unknowns/ Unsure
- ◯ No Response/ Refused

(8) If units is "other", please describe:

____

(8) Route

- ◯ Buccal
- ◯ Enteral
- ◯ Intradermal
- ◯ Intramuscular
- ◯ Intranasal
- ◯ Intravenous
- ◯ Ophthalmic
- ◯ Oral
- ◯ Otic
- ◯ Rectal
- ◯ Subcutaneous
- ◯ Sublingual
- ◯ Topical
- ◯ Transdermal
- ◯ Vaginally
- ◯ Other
- ◯ Unknown/ Unsure
- ◯ No Response/ Refused

(8) If route is "other", please describe:

____

(8) Frequency	◯ QD<br>◯ PRN<br>◯ BID<br>◯ TID<br>◯ QID<br>◯ Q 1 Hour<br>◯ Q 2 Hours<br>◯ Q 3 Hours<br>◯ Q 4 Hours<br>◯ Q 4-6 Hours<br>◯ Q 6 Hours<br>◯ Q 8 Hours<br>◯ Q 12 Hours<br>◯ QD PRN<br>◯ BID PRN<br>◯ TID PRN<br>◯ QID PRN<br>◯ Q 1 Hour PRN<br>◯ Q 2 Hours PRN<br>◯ Q 3 Hours PRN<br>◯ Q 4 Hours PRN<br>◯ Q 4-6 Hours PRN<br>◯ Q 6 Hours PRN<br>◯ Q 8 Hours PRN<br>◯ Q 12 Hours PRN<br>◯ Other<br>◯ Unknown/ Unsure<br>◯ Refused/ No Response
(8) If frequency is "other", please describe:	__________________________________
(8) Indication	◯ Diaper Rash<br>◯ Fussy<br>◯ Oral Thrush<br>◯ Other<br>◯ Unsure/ Unknown<br>◯ Refused/ No Response
(8) If indication is "other", please describe:	__________________________________
Infant Medication 9	
(9) Medication Name	◯ Erythromycin eye cream<br>◯ Gripe water<br>◯ Multivitamin<br>◯ Nystatin<br>◯ Simethicone<br>◯ Tri-Vi-Sol<br>◯ Vitamin D<br>◯ White petrolatum<br>◯ Zinc oxide cream<br>◯ Other<br>◯ Unsure/ Unknown<br>◯ No Response/ Refused
(9) If medication is "other", please describe:	__________________________________

(9) Dosage/Strength

____

(9) Units

◯ Capsule
◯ Cup
◯ Drop
◯ Gram
◯ Kilogram
◯ Liter
◯ Microgram
◯ Milligram
◯ Milliliter
◯ Ounce
◯ Pint
◯ Puff
◯ Quart
◯ Tablespoon
◯ Teaspoon
◯ Unit
◯ Other
◯ Unknowns/ Unsure
◯ No Response/ Refused

(9) If units is "other", please describe:

____

(9) Route

◯ Buccal
◯ Enteral
◯ Intradermal
◯ Intramuscular
◯ Intranasal
◯ Intravenous
◯ Ophthalmic
◯ Oral
◯ Otic
◯ Rectal
◯ Subcutaneous
◯ Sublingual
◯ Topical
◯ Transdermal
◯ Vaginally
◯ Other
◯ Unknown/ Unsure
◯ No Response/ Refused

(9) If route is "other", please describe:

____

(9) Frequency	◯ QD<br>◯ PRN<br>◯ BID<br>◯ TID<br>◯ QID<br>◯ Q 1 Hour<br>◯ Q 2 Hours<br>◯ Q 3 Hours<br>◯ Q 4 Hours<br>◯ Q 4-6 Hours<br>◯ Q 6 Hours<br>◯ Q 8 Hours<br>◯ Q 12 Hours<br>◯ QD PRN<br>◯ BID PRN<br>◯ TID PRN<br>◯ QID PRN<br>◯ Q 1 Hour PRN<br>◯ Q 2 Hours PRN<br>◯ Q 3 Hours PRN<br>◯ Q 4 Hours PRN<br>◯ Q 4-6 Hours PRN<br>◯ Q 6 Hours PRN<br>◯ Q 8 Hours PRN<br>◯ Q 12 Hours PRN<br>◯ Other<br>◯ Unknown/ Unsure<br>◯ Refused/ No Response
(9) If frequency is "other", please describe:	__________________________________
(9) Indication	◯ Diaper Rash<br>◯ Fussy<br>◯ Oral Thrush<br>◯ Other<br>◯ Unsure/ Unknown<br>◯ Refused/ No Response
(9) If indication is "other", please describe:	__________________________________
Infant Medication 10	
(10) Medication Name	◯ Erythromycin eye cream<br>◯ Gripe water<br>◯ Multivitamin<br>◯ Nystatin<br>◯ Simethicone<br>◯ Tri-Vi-Sol<br>◯ Vitamin D<br>◯ White petrolatum<br>◯ Zinc oxide cream<br>◯ Other<br>◯ Unsure/ Unknown<br>◯ No Response/ Refused
(10) If medication is "other", please describe:	__________________________________

(10) Dosage/Strength

____

(10) Units

◯ Capsule
◯ Cup
◯ Drop
◯ Gram
◯ Kilogram
◯ Liter
◯ Microgram
◯ Milligram
◯ Milliliter
◯ Ounce
◯ Pint
◯ Puff
◯ Quart
◯ Tablespoon
◯ Teaspoon
◯ Unit
◯ Other
◯ Unknowns/ Unsure
◯ No Response/ Refused

(10) If units is "other", please describe:

____

(10) Route

◯ Buccal
◯ Enteral
◯ Intradermal
◯ Intramuscular
◯ Intranasal
◯ Intravenous
◯ Ophthalmic
◯ Oral
◯ Otic
◯ Rectal
◯ Subcutaneous
◯ Sublingual
◯ Topical
◯ Transdermal
◯ Vaginally
◯ Other
◯ Unknown/ Unsure
◯ No Response/ Refused

(10) If route is "other", please describe:

____

(10) Frequency	◯ QD<br>◯ PRN<br>◯ BID<br>◯ TID<br>◯ QID<br>◯ Q 1 Hour<br>◯ Q 2 Hours<br>◯ Q 3 Hours<br>◯ Q 4 Hours<br>◯ Q 4-6 Hours<br>◯ Q 6 Hours<br>◯ Q 8 Hours<br>◯ Q 12 Hours<br>◯ QD PRN<br>◯ BID PRN<br>◯ TID PRN<br>◯ QID PRN<br>◯ Q 1 Hour PRN<br>◯ Q 2 Hours PRN<br>◯ Q 3 Hours PRN<br>◯ Q 4 Hours PRN<br>◯ Q 4-6 Hours PRN<br>◯ Q 6 Hours PRN<br>◯ Q 8 Hours PRN<br>◯ Q 12 Hours PRN<br>◯ Other<br>◯ Unknown/ Unsure<br>◯ Refused/ No Response
(10) If frequency is "other", please describe:	__________________________________
(10) Indication	◯ Diaper Rash<br>◯ Fussy<br>◯ Oral Thrush<br>◯ Other<br>◯ Unsure/ Unknown<br>◯ Refused/ No Response
(10) If indication is "other", please describe:	__________________________________
If the child is taking more than 10 medications, please record the name, dosage, units, route, frequency and indications of those medications here:	____

Please ask for the mother's permission to take a photo of the labels of any of her child's dietary supplements. Please indicate that these supplements belong to the child
