# Supplementary material for: Pediatric Respiratory and Enteric Virus Acquisition and Immunogenesis in US Mothers and Children Aged 0-2: PREVAIL Cohort Study
Source: JMIR Res Protoc. 2021 Feb 12;10(2):e22222. doi: 10.2196/22222 (PMC7910118; doi:10.2196/22222)
Supplement: Multimedia Appendix 5 [file resprot_v10i2e22222_app5.pdf]

Followup Month 24

Record ID	__________________________
Subject ID ([consent_arm_1][subjectid])	__________________________
Date	__________________________
SRC visit date (if different from above date)	__________________________
CRC Initials	__________________________
Comments<br><br>Mother's primary phone number: [consent_arm_1][mother_phone_1]<br><br>Mother's Name: [consent_arm_1][mother_first_name] [consent_arm_1][mother_last_name]	__________________________________
Visit Completion	◯ Mother & Infant completed visit<br>◯ Mother & Infant did NOT complete visit (Unable to reach to schedule an initial visit OR No show to first visit and unable to reschedule before the child is out of the visit window)<br>◯ Mother completed questionnaires by phone but did not complete SRC portion of visit

Thank you for your continued participation in the PREVAIL study and for making time for this clinic visit. I am going to ask you a series of questions that are part of the "Follow-up Questionnaire." These questions will help us to collect information about your health and the health of your child. Many of these questions were asked at your last clinic visit or over text message when your child was 21 months old and some of them will be for the entire 2 years since [week_0_arm_1][nbcrf_inf_name] was born.

Mother's Name: [consent_arm_1][mother_first_name] [consent_arm_1][mother_last_name]

Date of Month 18 Clinic Visit: [month_18_arm_1][m18_date]

Now I am going to ask you a few questions about how you have been feeding your infant

Are you still feeding breast milk to [week_0_arm_1][nbcrf_inf_name]?<br><br>This includes breast milk in ANY amount, whether exclusively or intermittently<br><br>month 18: [month_18_arm_1][m18_currentbf]<br>month 21: [month_21_arm_1][m21_currentbf]	◯ No<br>◯ Yes<br>◯ Unsure/Unknown<br>◯ Refused/No Response

If no, what was the date you stopped any breastfeeding?<br><br>Month 18: [month_18_arm_1][m18_date_stoppedbf]<br>Month 21: [month_21_arm_1][m21_date_stoppedbf]	______________________
Since [week_0_arm_1][nbcrf_inf_name] was 18 months old, how has your breast milk usually been fed to him/her?<br><br>month 18: [month_18_arm_1][m18_bf_method]<br>month 21: [month_21_arm_1][m21_bf_method]	◯ At the breast<br>◯ Pumped, then fed by a bottle<br>◯ A combination of at the breast and pumped milk
Has [week_0_arm_1][nbcrf_inf_name] ever been fed formula?<br><br>Month 18: [month_18_arm_1][m18_everformula]<br>Month 21: [month_21_arm_1][m21_everformula]	◯ No<br>◯ Yes<br>◯ Unsure/Unknown<br>◯ Refused/No Response
On what date was formula first introduced?<br><br>Month 18: [month_18_arm_1][m18_date_formula]<br>Month 21: [month_21_arm_1][m21_date_formula]	______________________<br>(Use Calendar to assist the mother)
Verify Address<br><br>Street Address<br><br>[consent_arm_1][address_street]	______________________
City<br><br>[consent_arm_1][address_city]	______________________
State<br><br>[consent_arm_1][address_state]	◯ IN<br>◯ KY<br>◯ OH
Zipcode<br><br>[consent_arm_1][address_zip]	______________________
Latitude	______________________
Longitude	______________________
Census Tract ID	______________________
Deprivation Index	______________________

Maternal Primary Care Provider

Since the birth of your child, have you seen a primary care provider?

Month 12: [month_12_arm_1][m12_mat_pcp]
Month 18: [month_18_arm_1][m18_mat_pcp]

- ◯ Yes
- ◯ No

Please list the names of primary care practices at which you have received care in the past two years:

Month 12: [month_12_arm_1][m12_mat_pcp_practice]
Month 18: [month_18_arm_1][m18_mat_pcp_practice]

____

Please list the name/names of primary care providers that have provided care to you in the past two years:

Month 12: [month_12_arm_1][m12_mat_pcp_name]
Month 18: [month_18_arm_1][m18_mat_pcp_name]

____

Since [week_0_arm_1][nbcrf_inf_name] was born, have you gotten a flu shot?

- ◯ No
- ◯ Yes
- ◯ Unknown
- ◯ Refused

Since [week_0_arm_1][nbcrf_inf_name] was born, how many times have you gotten a flu shot?

- ◯ 1
- ◯ 2
- ◯ 3
- ◯ 4

Date of first flu shot

(CRC: use MM/01/YYYY format. Mom's best guess if fine)

____

At what kind of place did you receive the first flu vaccination?

- ◯ My OB/GYN's office
- ◯ My midwife's office
- ◯ My family doctor or other doctor's office
- ◯ Health department clinic
- ◯ Other clinic or health center
- ◯ Hospital
- ◯ Other medically-related place
- ◯ A pharmacy or drug store (like a Walgreens or CVS)
- ◯ A supermarket, grocery store, or superstore (like a Wal-Mart or Target)
- ◯ Workplace
- ◯ Elementary/Middle/High School
- ◯ Other non-medically related place
- ◯ Unsure/Unknown

Please specify "other medically-related place":

____

Please specify "other non-medically related place":

____

Please provide the name and location of the vaccination provider:
(i.e. Walgreens, Clifton
or name/location of OB practice
or name/location of Employer etc)

____

Did you employer at this time require you to get a flu shot?

◯ No
◯ Yes
◯ Unknown
◯ Refused

Date of second flu shot

(CRC: use MM/01/YYYY format. Mom's best guess if fine)

____

At what kind of place did you receive the 2nd flu vaccination?

◯ My OB/GYN's office
◯ My midwife's office
◯ My family doctor or other doctor's office
◯ Health department clinic
◯ Other clinic or health center
◯ Hospital
◯ Other medically-related place
◯ A pharmacy or drug store (like a Walgreens or CVS)
◯ A supermarket, grocery store, or superstore (like a Wal-Mart or Target)
◯ Workplace
◯ Elementary/Middle/High School
◯ Other non-medically related place
◯ Unsure/Unknown

Please specify "other medically-related place":

____

Please specify "other non-medically related place":

____

Please provide the name and location of the vaccination provider:
(i.e. Walgreens, Clifton
or name/location of OB practice
or name/location of Employer etc)

____

Did you employer at this time require you to get a flu shot?

◯ No
◯ Yes
◯ Unknown
◯ Refused

Date of 3rd flu shot

(CRC: use MM/01/YYYY format. Mom's best guess if fine)

____

At what kind of place did you receive the 3rd flu vaccination?

◯ My OB/GYN's office
◯ My midwife's office
◯ My family doctor or other doctor's office
◯ Health department clinic
◯ Other clinic or health center
◯ Hospital
◯ Other medically-related place
◯ A pharmacy or drug store (like a Walgreens or CVS)
◯ A supermarket, grocery store, or superstore (like a Wal-Mart or Target)
◯ Workplace
◯ Elementary/Middle/High School
◯ Other non-medically related place
◯ Unsure/Unknown

Please specify "other medically-related place": __________________________________

Please specify "other non-medically related place": __________________________________

Please provide the name and location of the vaccination provider:
(i.e. Walgreens, Clifton
or name/location of OB practice
or name/location of Employer etc) __________________________________

Did you employer at this time require you to get a flu shot?

- ◯ No
- ◯ Yes
- ◯ Unknown
- ◯ Refused

Date of 4th flu shot __________________________________

(CRC: use MM/01/YYYY format. Mom's best guess if fine)

At what kind of place did you receive the 4th flu vaccination?

- ◯ My OB/GYN's office
- ◯ My midwife's office
- ◯ My family doctor or other doctor's office
- ◯ Health department clinic
- ◯ Other clinic or health center
- ◯ Hospital
- ◯ Other medically-related place
- ◯ A pharmacy or drug store (like a Walgreens or CVS)
- ◯ A supermarket, grocery store, or superstore (like a Wal-Mart or Target)
- ◯ Workplace
- ◯ Elementary/Middle/High School
- ◯ Other non-medically related place
- ◯ Unsure/Unknown

Please specify "other medically-related place": __________________________________

Please specify "other non-medically related place": __________________________________

Please provide the name and location of the vaccination provider:
(i.e. Walgreens, Clifton
or name/location of OB practice
or name/location of Employer etc) __________________________________

Did you employer at this time require you to get a flu shot?

- ◯ No
- ◯ Yes
- ◯ Unknown
- ◯ Refused

Types of Medications

Since your child was 18 months old, have you taken any of the following:

Vitamins or dietary supplements<br><br>Month 18: [month_18_arm_1][m18_mat_vitamin_supplement]	◯ No<br>◯ Yes<br>◯ Unsure/Unknown<br>◯ Refused/No Response
Over the counter medications<br><br>Month 18: [month_18_arm_1][m18_mat_otcmeds]	◯ No<br>◯ Yes<br>◯ Unsure/Unknown<br>◯ Refused/No Response
Herbal or folk remedies<br><br>Month 18: [month_18_arm_1][m18_mat_herbalremedies]	◯ No<br>◯ Yes<br>◯ Unsure/Unknown<br>◯ Refused/No Response
Prescription medications<br><br>Month 18: [month_18_arm_1][m18_mat_prescriptions]	◯ No<br>◯ Yes<br>◯ Unsure/Unknown<br>◯ Refused/No Response

Maternal Medication List

Please list any medications that you have taken since [week_0_arm_1][nbcrf_inf_name] was 18 months old.

How many vitamins/dietary supplements, over the counter medications, herbal/folk remedies or prescription medications is the mother taking in total?<br><br>Month 18: [month_18_arm_1][m18_maternalmedications]	◯ 1<br>◯ 2<br>◯ 3<br>◯ 4<br>◯ 5<br>◯ 6<br>◯ 7<br>◯ 8<br>◯ 9<br>◯ 10<br>◯ 11 or More

Maternal Medication 1

(1) Medication Name

Month 18: [month_18_arm_1] [m18_matmed_name1]

- ◯ DHA
- ◯ Docusate sodium
- ◯ Ibuprofen
- ◯ Iron
- ◯ Miralax
- ◯ Oxycodone- acetaminophen (percocet)
- ◯ Prenatal vitamins
- ◯ Prenatal vitamins + DHA
- ◯ Tylenol
- ◯ Vitamin D
- ◯ Antimicrobials
- ◯ Other vitamin
- ◯ Birth control pills
- ◯ Antihistamine
- ◯ Herbal Supplement
- ◯ Other
- ◯ Unsure/ Unknown
- ◯ Refused/ No Response

---

(1) If medication is "antimicrobial" please describe

- ◯ Acyclovir
- ◯ Amoxicillin
- ◯ Ancef/ Cefazolin
- ◯ Azithromycin
- ◯ Clindamycin
- ◯ Diflucan/ Fluconazole
- ◯ Flagyl
- ◯ Macrobid
- ◯ Penicillin G
- ◯ Vancomycin
- ◯ Oseltamivir/Tamiflu
- ◯ Other
- ◯ Unsure/ Unknown
- ◯ No Response/ Refused

---

(1) If medication is "other", please describe:

____

---

(1) Dosage/Strength

Month 18: [month_18_arm_1][m18_matmed_dose1]

____

---

(1) Units

Month 18: [month_18_arm_1][m18_matmed_units1]

____

---

(1) Route

Month 18: [month_18_arm_1][m18_matmed_route1]

____

---

(1) Frequency

Month 18: [month_18_arm_1][m18_matmed_freq1]

____

(1) Indication<br><br>Month 18: [month_18_arm_1][m18_matmed_indication1]	◯ Anti-Inflammatory<br>◯ Constipation<br>◯ Depression/Anxiety<br>◯ Headache<br>◯ Low Iron<br>◯ Pain<br>◯ Vitamins<br>◯ Allergies<br>◯ Hypertension<br>◯ Birth Control<br>◯ Lactation Support<br>◯ Mastitis<br>◯ Influenza<br>◯ Other<br>◯ Unknown/ Unsure<br>◯ No Response/ Refused
(1) If indication is "other", please describe:	__________________________________
Maternal Medication 2	
(2) Medication Name<br><br>Month 18: [month_18_arm_1][m18_matmed_name2]	◯ DHA<br>◯ Docusate sodium<br>◯ Ibuprofen<br>◯ Iron<br>◯ Miralax<br>◯ Oxycodone- acetaminophen (percocet)<br>◯ Prenatal vitamins<br>◯ Prenatal vitamins + DHA<br>◯ Tylenol<br>◯ Vitamin D<br>◯ Antimicrobials<br>◯ Other vitamin<br>◯ Birth control pills<br>◯ Antihistamine<br>◯ Herbal Supplement<br>◯ Other<br>◯ Unsure/ Unknown<br>◯ Refused/ No Response
(2) If medication is "antimicrobial" please describe	◯ Acyclovir<br>◯ Amoxicillin<br>◯ Ancef/ Cefazolin<br>◯ Azithromycin<br>◯ Clindamycin<br>◯ Diflucan/ Fluconazole<br>◯ Flagyl<br>◯ Macrobid<br>◯ Penicillin G<br>◯ Vancomycin<br>◯ Oseltamivir/Tamiflu<br>◯ Other<br>◯ Unsure/ Unknown<br>◯ No Response/ Refused
(2) If medication is "other", please describe:	__________________________________

(2) Dosage/Strength

Month 18: [month_18_arm_1][m18_matmed_dose2] ______________________________

(2) Units

Month 18: [month_18_arm_1][m18_matmed_units2] ______________________________

(2) Route

Month 18: [month_18_arm_1][m18_matmed_route2] ______________________________

(2) Frequency

Month 18: [month_18_arm_1][m18_matmed_freq2] ______________________________

(2) Indication

Month 18: [month_18_arm_1][m18_matmed_indication2]

- ◯ Anti-Inflammatory
- ◯ Constipation
- ◯ Depression/Anxiety
- ◯ Headache
- ◯ Low Iron
- ◯ Pain
- ◯ Vitamins
- ◯ Allergies
- ◯ Hypertension
- ◯ Birth Control
- ◯ Lactation Support
- ◯ Mastitis
- ◯ Influenza
- ◯ Other
- ◯ Unknown/ Unsure
- ◯ No Response/ Refused

(2) If indication is "other", please describe: ______________________________

Maternal Medication 3

(3) Medication Name

Month 18: [month_18_arm_1][m18_matmed_name3]

- ◯ DHA
- ◯ Docusate sodium
- ◯ Ibuprofen
- ◯ Iron
- ◯ Miralax
- ◯ Oxycodone- acetaminophen (percocet)
- ◯ Prenatal vitamins
- ◯ Prenatal vitamins + DHA
- ◯ Tylenol
- ◯ Vitamin D
- ◯ Antimicrobials
- ◯ Other vitamin
- ◯ Birth control pills
- ◯ Antihistamine
- ◯ Herbal Supplement
- ◯ Other
- ◯ Unsure/ Unknown
- ◯ Refused/ No Response

(3) If medication is "antimicrobial" please describe	◯ Acyclovir<br>◯ Amoxicillin<br>◯ Ancef/ Cefazolin<br>◯ Azithromycin<br>◯ Clindamycin<br>◯ Diflucan/ Fluconazole<br>◯ Flagyl<br>◯ Macrobid<br>◯ Penicillin G<br>◯ Vancomycin<br>◯ Oseltamivir/Tamiflu<br>◯ Other<br>◯ Unsure/ Unknown<br>◯ No Response/ Refused
(3) If medication is "other", please describe:	__________________________________
(3) Dosage/Strength<br><br>Month 18: [month_18_arm_1][m18_matmed_dose3]	__________________________________
(3) Units<br><br>Month 18: [month_18_arm_1][m18_matmed_units3]	__________________________________
(3) Route<br><br>Month 18: [month_18_arm_1][m18_matmed_route3]	__________________________________
(3) Frequency<br><br>Month 18: [month_18_arm_1][m18_matmed_freq3]	__________________________________
(3) Indication<br><br>Month 18: [month_18_arm_1][m18_matmed_indication3]	◯ Anti-Inflammatory<br>◯ Constipation<br>◯ Depression/Anxiety<br>◯ Headache<br>◯ Low Iron<br>◯ Pain<br>◯ Vitamins<br>◯ Allergies<br>◯ Hypertension<br>◯ Birth Control<br>◯ Lactation Support<br>◯ Mastitis<br>◯ Influenza<br>◯ Other<br>◯ Unknown/ Unsure<br>◯ No Response/ Refused
(3) If indication is "other", please describe:	__________________________________

Maternal Medication 4

(4) Medication Name<br><br>Month 18: [month_18_arm_1][m18_matmed_name4]	◯ DHA<br>◯ Docusate sodium<br>◯ Ibuprofen<br>◯ Iron<br>◯ Miralax<br>◯ Oxycodone- acetaminophen (percocet)<br>◯ Prenatal vitamins<br>◯ Prenatal vitamins + DHA<br>◯ Tylenol<br>◯ Vitamin D<br>◯ Antimicrobials<br>◯ Other vitamin<br>◯ Birth control pills<br>◯ Antihistamine<br>◯ Herbal Supplement<br>◯ Other<br>◯ Unsure/ Unknown<br>◯ Refused/ No Response
(4) If medication is "antimicrobial" please describe	◯ Acyclovir<br>◯ Amoxicillin<br>◯ Ancef/ Cefazolin<br>◯ Azithromycin<br>◯ Clindamycin<br>◯ Diflucan/ Fluconazole<br>◯ Flagyl<br>◯ Macrobid<br>◯ Penicillin G<br>◯ Vancomycin<br>◯ Oseltamivir/Tamiflu<br>◯ Other<br>◯ Unsure/ Unknown<br>◯ No Response/ Refused
(4) If medication is "other", please describe:	__________________________________
(4) Dosage/Strength<br><br>Month 18: [month_18_arm_1][m18_matmed_dose4]	__________________________________
(4) Units<br><br>Month 18: [month_18_arm_1][m18_matmed_units4]	__________________________________
(4) Route<br><br>Month 18: [month_18_arm_1][m18_matmed_route4]	__________________________________
(4) Frequency<br><br>Month 18: [month_18_arm_1][m18_matmed_freq4]	__________________________________

(4) Indication

Month 18: [month_18_arm_1][m18_matmed_indication4]

- ◯ Anti-Inflammatory
- ◯ Constipation
- ◯ Depression/Anxiety
- ◯ Headache
- ◯ Low Iron
- ◯ Pain
- ◯ Vitamins
- ◯ Allergies
- ◯ Hypertension
- ◯ Birth Control
- ◯ Lactation Support
- ◯ Mastitis
- ◯ Influenza
- ◯ Other
- ◯ Unknown/ Unsure
- ◯ No Response/ Refused

(4) If indication is "other", please describe:

____

Maternal Medication 5

(5) Medication Name

Month 18: [month_18_arm_1][m18_matmed_name5]

- ◯ DHA
- ◯ Docusate sodium
- ◯ Ibuprofen
- ◯ Iron
- ◯ Miralax
- ◯ Oxycodone- acetaminophen (percocet)
- ◯ Prenatal vitamins
- ◯ Prenatal vitamins + DHA
- ◯ Tylenol
- ◯ Vitamin D
- ◯ Antimicrobials
- ◯ Other vitamin
- ◯ Birth control pills
- ◯ Antihistamine
- ◯ Herbal Supplement
- ◯ Other
- ◯ Unsure/ Unknown
- ◯ Refused/ No Response

(5) If medication is "antimicrobial" please describe

- ◯ Acyclovir
- ◯ Amoxicillin
- ◯ Ancef/ Cefazolin
- ◯ Azithromycin
- ◯ Clindamycin
- ◯ Diflucan/ Fluconazole
- ◯ Flagyl
- ◯ Macrobid
- ◯ Penicillin G
- ◯ Vancomycin
- ◯ Oseltamivir/Tamiflu
- ◯ Other
- ◯ Unsure/ Unknown
- ◯ No Response/ Refused

(5) If medication is "other", please describe:

____

(5) Dosage/Strength

Month 18: [month_18_arm_1][m18_matmed_dose5] __________________________________

(5) Units

Month 18: [month_18_arm_1][m18_matmed_units5] __________________________________

(5) Route

Month 18: [month_18_arm_1][m18_matmed_route5] __________________________________

(5) Frequency

Month 18: [month_18_arm_1][m18_matmed_freq5] __________________________________

(5) Indication

Month 18: [month_18_arm_1][m18_matmed_indication5]

- ◯ Anti-Inflammatory
- ◯ Constipation
- ◯ Depression/Anxiety
- ◯ Headache
- ◯ Low Iron
- ◯ Pain
- ◯ Vitamins
- ◯ Allergies
- ◯ Hypertension
- ◯ Birth Control
- ◯ Lactation Support
- ◯ Mastitis
- ◯ Influenza
- ◯ Other
- ◯ Unknown/ Unsure
- ◯ No Response/ Refused

(5) If indication is "other", please describe: __________________________________

Maternal Medication 6

(6) Medication Name

Month 18: [month_18_arm_1][m18_matmed_name6]

- ◯ DHA
- ◯ Docusate sodium
- ◯ Ibuprofen
- ◯ Iron
- ◯ Miralax
- ◯ Oxycodone- acetaminophen (percocet)
- ◯ Prenatal vitamins
- ◯ Prenatal vitamins + DHA
- ◯ Tylenol
- ◯ Vitamin D
- ◯ Antimicrobials
- ◯ Other vitamin
- ◯ Birth control pills
- ◯ Antihistamine
- ◯ Herbal Supplement
- ◯ Other
- ◯ Unsure/ Unknown
- ◯ Refused/ No Response

(6) If medication is "antimicrobial" please describe	◯ Acyclovir<br>◯ Amoxicillin<br>◯ Ancef/ Cefazolin<br>◯ Azithromycin<br>◯ Clindamycin<br>◯ Diflucan/ Fluconazole<br>◯ Flagyl<br>◯ Macrobid<br>◯ Penicillin G<br>◯ Vancomycin<br>◯ Oseltamivir/Tamiflu<br>◯ Other<br>◯ Unsure/ Unknown<br>◯ No Response/ Refused
(6) If medication is "other", please describe:	__________________________________
(6) Dosage/Strength<br><br>Month 18: [month_18_arm_1][m18_matmed_dose6]	__________________________________
(6) Units<br><br>Month 18: [month_18_arm_1][m18_matmed_units6]	__________________________________
(6) Route<br><br>Month 18: [month_18_arm_1][m18_matmed_route6]	__________________________________
(6) Frequency<br><br>Month 18: [month_18_arm_1][m18_matmed_freq6]	__________________________________
(6) Indication<br><br>Month 18: [month_18_arm_1][m18_matmed_indication6]	◯ Anti-Inflammatory<br>◯ Constipation<br>◯ Depression/Anxiety<br>◯ Headache<br>◯ Low Iron<br>◯ Pain<br>◯ Vitamins<br>◯ Allergies<br>◯ Hypertension<br>◯ Birth Control<br>◯ Lactation Support<br>◯ Mastitis<br>◯ Influenza<br>◯ Other<br>◯ Unknown/ Unsure<br>◯ No Response/ Refused
(6) If indication is "other", please describe:	__________________________________
Maternal Medication 7	

(7) Medication Name

Month 18: [month_18_arm_1][m18_matmed_name7]

◯ DHA
◯ Docusate sodium
◯ Ibuprofen
◯ Iron
◯ Miralax
◯ Oxycodone- acetaminophen (percocet)
◯ Prenatal vitamins
◯ Prenatal vitamins + DHA
◯ Tylenol
◯ Vitamin D
◯ Antimicrobials
◯ Other vitamin
◯ Birth control pills
◯ Antihistamine
◯ Herbal Supplement
◯ Other
◯ Unsure/ Unknown
◯ Refused/ No Response

(7) If medication is "antimicrobial" please describe

◯ Acyclovir
◯ Amoxicillin
◯ Ancef/ Cefazolin
◯ Azithromycin
◯ Clindamycin
◯ Diflucan/ Fluconazole
◯ Flagyl
◯ Macrobid
◯ Penicillin G
◯ Vancomycin
◯ Oseltamivir/Tamiflu
◯ Other
◯ Unsure/ Unknown
◯ No Response/ Refused

(7) If medication is "other", please describe:

____

(7) Dosage/Strength

Month 18: [month_18_arm_1][m18_matmed_dose7]

____

(7) Units

Month 18: [month_18_arm_1][m18_matmed_units7]

____

(7) Route

Month 18: [month_18_arm_1][m18_matmed_route7]

____

(7) Frequency

Month 18: [month_18_arm_1][m18_matmed_freq7]

____

(7) Indication<br><br>Month 18: [month_18_arm_1][m18_matmed_indication7]	◯ Anti-Inflammatory<br>◯ Constipation<br>◯ Depression/Anxiety<br>◯ Headache<br>◯ Low Iron<br>◯ Pain<br>◯ Vitamins<br>◯ Allergies<br>◯ Hypertension<br>◯ Birth Control<br>◯ Lactation Support<br>◯ Mastitis<br>◯ Influenza<br>◯ Other<br>◯ Unknown/ Unsure<br>◯ No Response/ Refused
(7) If indication is "other", please describe:	________________________
Maternal Medication 8	
(8) Medication Name<br><br>Month 18: [month_18_arm_1][m18_matmed_name8]	◯ DHA<br>◯ Docusate sodium<br>◯ Ibuprofen<br>◯ Iron<br>◯ Miralax<br>◯ Oxycodone- acetaminophen (percocet)<br>◯ Prenatal vitamins<br>◯ Prenatal vitamins + DHA<br>◯ Tylenol<br>◯ Vitamin D<br>◯ Antimicrobials<br>◯ Other vitamin<br>◯ Birth control pills<br>◯ Antihistamine<br>◯ Herbal Supplement<br>◯ Other<br>◯ Unsure/ Unknown<br>◯ Refused/ No Response
(8) If medication is "antimicrobial" please describe	◯ Acyclovir<br>◯ Amoxicillin<br>◯ Ancef/ Cefazolin<br>◯ Azithromycin<br>◯ Clindamycin<br>◯ Diflucan/ Fluconazole<br>◯ Flagyl<br>◯ Macrobid<br>◯ Penicillin G<br>◯ Vancomycin<br>◯ Oseltamivir/Tamiflu<br>◯ Other<br>◯ Unsure/ Unknown<br>◯ No Response/ Refused
(8) If medication is "other", please describe:	________________________

(8) Dosage/Strength

Month 18: [month_18_arm_1][m18_matmed_dose8] ______________________

(8) Units

Month 18: [month_18_arm_1][m18_matmed_units8] ______________________

(8) Route

Month 18: [month_18_arm_1][m18_matmed_route8] ______________________

(8) Frequency

Month 18: [month_18_arm_1][m18_matmed_freq8] ______________________

(8) Indication

Month 18: [month_18_arm_1][m18_matmed_indication8]

- ◯ Anti-Inflammatory
- ◯ Constipation
- ◯ Depression/Anxiety
- ◯ Headache
- ◯ Low Iron
- ◯ Pain
- ◯ Vitamins
- ◯ Allergies
- ◯ Hypertension
- ◯ Birth Control
- ◯ Lactation Support
- ◯ Mastitis
- ◯ Influenza
- ◯ Other
- ◯ Unknown/ Unsure
- ◯ No Response/ Refused

(8) If indication is "other", please describe: ______________________

Maternal Medication 9

(9) Medication Name

Month 18: [month_18_arm_1][m18_matmed_name9]

- ◯ DHA
- ◯ Docusate sodium
- ◯ Ibuprofen
- ◯ Iron
- ◯ Miralax
- ◯ Oxycodone- acetaminophen (percocet)
- ◯ Prenatal vitamins
- ◯ Prenatal vitamins + DHA
- ◯ Tylenol
- ◯ Vitamin D
- ◯ Antimicrobials
- ◯ Other vitamin
- ◯ Birth control pills
- ◯ Antihistamine
- ◯ Herbal Supplement
- ◯ Other
- ◯ Unsure/ Unknown
- ◯ Refused/ No Response

(9) If medication is "antimicrobial" please describe

- ◯ Acyclovir
- ◯ Amoxicillin
- ◯ Ancef/ Cefazolin
- ◯ Azithromycin
- ◯ Clindamycin
- ◯ Diflucan/ Fluconazole
- ◯ Flagyl
- ◯ Macrobid
- ◯ Penicillin G
- ◯ Vancomycin
- ◯ Oseltamivir/Tamiflu
- ◯ Other
- ◯ Unsure/ Unknown
- ◯ No Response/ Refused

(9) If medication is "other", please describe:

____

(9) Dosage/Strength

Month 18: [month_18_arm_1][m18_matmed_dose9]

____

(9) Units

Month 18: [month_18_arm_1][m18_matmed_units9]

____

(9) Route

Month 18: [month_18_arm_1][m18_matmed_route9]

____

(9) Frequency

Month 18: [month_18_arm_1][m18_matmed_freq9]

____

(9) Indication

Month 18: [month_18_arm_1][m18_matmed_indication9]

- ◯ Anti-Inflammatory
- ◯ Constipation
- ◯ Depression/Anxiety
- ◯ Headache
- ◯ Low Iron
- ◯ Pain
- ◯ Vitamins
- ◯ Allergies
- ◯ Hypertension
- ◯ Birth Control
- ◯ Lactation Support
- ◯ Mastitis
- ◯ Influenza
- ◯ Other
- ◯ Unknown/ Unsure
- ◯ No Response/ Refused

(9) If indication is "other", please describe:

____

Maternal Medication 10

(10) Medication Name<br><br>Month 18: [month_18_arm_1][m18_matmed_name10]	◯ DHA<br>◯ Docusate sodium<br>◯ Ibuprofen<br>◯ Iron<br>◯ Miralax<br>◯ Oxycodone- acetaminophen (percocet)<br>◯ Prenatal vitamins<br>◯ Prenatal vitamins + DHA<br>◯ Tylenol<br>◯ Vitamin D<br>◯ Antimicrobials<br>◯ Other vitamin<br>◯ Birth control pills<br>◯ Antihistamine<br>◯ Herbal Supplement<br>◯ Other<br>◯ Unsure/ Unknown<br>◯ Refused/ No Response
(10) If medication is "antimicrobial" please describe	◯ Acyclovir<br>◯ Amoxicillin<br>◯ Ancef/ Cefazolin<br>◯ Azithromycin<br>◯ Clindamycin<br>◯ Diflucan/ Fluconazole<br>◯ Flagyl<br>◯ Macrobid<br>◯ Penicillin G<br>◯ Vancomycin<br>◯ Oseltamivir/Tamiflu<br>◯ Other<br>◯ Unsure/ Unknown<br>◯ No Response/ Refused
(10) If medication is "other", please describe:	________________________
(10) Dosage/Strength<br><br>Month 18: [month_18_arm_1][m18_matmed_dose10]	________________________
(10) Units<br><br>Month 18: [month_18_arm_1][m18_matmed_units10]	________________________
(10) Route<br><br>Month 18: [month_18_arm_1][m18_matmed_route10]	________________________
(10) Frequency<br><br>Month 18: [month_18_arm_1][m18_matmed_freq10]	________________________

(10) Indication<br><br>Month 18: [month_18_arm_1][m18_matmed_indication10]	◯ Anti-Inflammatory<br>◯ Constipation<br>◯ Depression/Anxiety<br>◯ Headache<br>◯ Low Iron<br>◯ Pain<br>◯ Vitamins<br>◯ Allergies<br>◯ Hypertension<br>◯ Birth Control<br>◯ Lactation Support<br>◯ Mastitis<br>◯ Influenza<br>◯ Other<br>◯ Unknown/ Unsure<br>◯ No Response/ Refused
(10) If indication is "other", please describe:	__________________________________
If the mother is taking more than 10 medications, please record the name, dosage, units, route, frequency and indications of those medications here:	____

Household Structure

Including yourself, how many people live in your household?<br><br>Month 18: [month_18_arm_1][m18_household]	◯ 1<br>◯ 2<br>◯ 3<br>◯ 4<br>◯ 5<br>◯ 6<br>◯ 7<br>◯ More than 7 people<br>◯ Unsure/Unknown<br>◯ Refused/No Response

Please list all household members, including the mother and study infant:

Household Member 1

(1) Household member's relation to the child<br><br>Month 18: [month_18_arm_1][m18_hhmember1_relation]	◯ Study Infant<br>◯ Study Mother<br>◯ Father<br>◯ Sister<br>◯ Brother<br>◯ Aunt<br>◯ Uncle<br>◯ Cousin<br>◯ Maternal Grandmother<br>◯ Maternal Grandfather<br>◯ Paternal Grandmother<br>◯ Paternal Grandfather<br>◯ Maternal Great Grandmother<br>◯ Maternal Great Grandfather<br>◯ Paternal Great Grandmother<br>◯ Paternal Great Grandfather<br>◯ Family Friend<br>◯ Significant Other (study mother's significant other that is not the infant's father)<br>◯ Other
(1) If relation is "other", please describe:	______________________________
(1) Age<br><br>Month 18: [month_18_arm_1][m18_hhmember1_age]	______________________________
(1) Sex<br><br>Month 18: [month_18_arm_1][m18_hhmember1_sex]	◯ Male<br>◯ Female
Household Member 2	
(2) Household member's relation to the child<br><br>Month 18: [month_18_arm_1][m18_hhmember2_relation]	◯ Study Infant<br>◯ Study Mother<br>◯ Father<br>◯ Sister<br>◯ Brother<br>◯ Aunt<br>◯ Uncle<br>◯ Cousin<br>◯ Maternal Grandmother<br>◯ Maternal Grandfather<br>◯ Paternal Grandmother<br>◯ Paternal Grandfather<br>◯ Maternal Great Grandmother<br>◯ Maternal Great Grandfather<br>◯ Paternal Great Grandmother<br>◯ Paternal Great Grandfather<br>◯ Family Friend<br>◯ Significant Other (study mother's significant other that is not the infant's father)<br>◯ Other
(2) If relation is "other", please describe:	______________________________

(2) Age

Month 18: [month_18_arm_1][m18_hhmember2_age]

____

(2) Sex

Month 18: [month_18_arm_1][m18_hhmember2_sex]

- ◯ Male
- ◯ Female

Household Member 3

(3) Household member's relation to the child

Month 18: [month_18_arm_1][m18_hhmember3_relation]

- ◯ Study Infant
- ◯ Study Mother
- ◯ Father
- ◯ Sister
- ◯ Brother
- ◯ Aunt
- ◯ Uncle
- ◯ Cousin
- ◯ Maternal Grandmother
- ◯ Maternal Grandfather
- ◯ Paternal Grandmother
- ◯ Paternal Grandfather
- ◯ Maternal Great Grandmother
- ◯ Maternal Great Grandfather
- ◯ Paternal Great Grandmother
- ◯ Paternal Great Grandfather
- ◯ Family Friend
- ◯ Significant Other (study mother's significant other that is not the infant's father)
- ◯ Other

(3) If relation is "other", please describe:

____

(3) Age

Month 18: [month_18_arm_1][m18_hhmember3_age]

____

(3) Sex

Month 18: [month_18_arm_1][m18_hhmember3_sex]

- ◯ Male
- ◯ Female

Household Member 4

(4) Household member's relation to the child<br><br>Month 18: [month_18_arm_1][m18_hhmember4_relation]	◯ Study Infant<br>◯ Study Mother<br>◯ Father<br>◯ Sister<br>◯ Brother<br>◯ Aunt<br>◯ Uncle<br>◯ Cousin<br>◯ Maternal Grandmother<br>◯ Maternal Grandfather<br>◯ Paternal Grandmother<br>◯ Paternal Grandfather<br>◯ Maternal Great Grandmother<br>◯ Maternal Great Grandfather<br>◯ Paternal Great Grandmother<br>◯ Paternal Great Grandfather<br>◯ Family Friend<br>◯ Significant Other (study mother's significant other that is not the infant's father)<br>◯ Other
(4) If relation is "other", please describe:	__________________________________
(4) Age<br><br>Month 18: [month_18_arm_1][m18_hhmember4_age]	__________________________________
(4) Sex<br><br>Month 18: [month_18_arm_1][m18_hhmember4_sex]	◯ Male<br>◯ Female
Household Member 5	
(5) Household member's relation to the child<br><br>Month 18: [month_18_arm_1][m18_hhmember5_relation]	◯ Study Infant<br>◯ Study Mother<br>◯ Father<br>◯ Sister<br>◯ Brother<br>◯ Aunt<br>◯ Uncle<br>◯ Cousin<br>◯ Maternal Grandmother<br>◯ Maternal Grandfather<br>◯ Paternal Grandmother<br>◯ Paternal Grandfather<br>◯ Maternal Great Grandmother<br>◯ Maternal Great Grandfather<br>◯ Paternal Great Grandmother<br>◯ Paternal Great Grandfather<br>◯ Family Friend<br>◯ Significant Other (study mother's significant other that is not the infant's father)<br>◯ Other
(5) If relation is "other", please describe:	__________________________________

(5) Age

Month 18: [month_18_arm_1][m18_hhmember5_age] __________________________________

(5) Sex

Month 18: [month_18_arm_1][m18_hhmember5_sex]

- ◯ Male
- ◯ Female

Household Member 6

(6) Household member's relation to the child

Month 18: [month_18_arm_1][m18_hhmember6_relation]

- ◯ Study Infant
- ◯ Study Mother
- ◯ Father
- ◯ Sister
- ◯ Brother
- ◯ Aunt
- ◯ Uncle
- ◯ Cousin
- ◯ Maternal Grandmother
- ◯ Maternal Grandfather
- ◯ Paternal Grandmother
- ◯ Paternal Grandfather
- ◯ Maternal Great Grandmother
- ◯ Maternal Great Grandfather
- ◯ Paternal Great Grandmother
- ◯ Paternal Great Grandfather
- ◯ Family Friend
- ◯ Significant Other (study mother's significant other that is not the infant's father)
- ◯ Other

(6) If relation is "other", please describe: __________________________________

(6) Age

Month 18: [month_18_arm_1][m18_hhmember6_age] __________________________________

(6) Sex

Month 18: [month_18_arm_1][m18_hhmember6_sex]

- ◯ Male
- ◯ Female

Household Member 7

(7) Household member's relation to the child<br><br>Month 18: [month_18_arm_1][m18_hhmember7_relation]	◯ Study Infant<br>◯ Study Mother<br>◯ Father<br>◯ Sister<br>◯ Brother<br>◯ Aunt<br>◯ Uncle<br>◯ Cousin<br>◯ Maternal Grandmother<br>◯ Maternal Grandfather<br>◯ Paternal Grandmother<br>◯ Paternal Grandfather<br>◯ Maternal Great Grandmother<br>◯ Maternal Great Grandfather<br>◯ Paternal Great Grandmother<br>◯ Paternal Great Grandfather<br>◯ Family Friend<br>◯ Significant Other (study mother's significant other that is not the infant's father)<br>◯ Other
(7) If relation is "other", please describe:	______________________________
(7) Age<br><br>Month 18: [month_18_arm_1][m18_hhmember7_age]	______________________________
(7) Sex<br><br>Month 18: [month_18_arm_1][m18_hhmember7_sex]	◯ Male<br>◯ Female
If there are more than 7 people living in your household, please describe their relationship to the child, their age in years and their gender:	______________________________
(8) Household member's relation to the child	◯ Study Infant<br>◯ Study Mother<br>◯ Father<br>◯ Sister<br>◯ Brother<br>◯ Aunt<br>◯ Uncle<br>◯ Cousin<br>◯ Maternal Grandmother<br>◯ Maternal Grandfather<br>◯ Paternal Grandmother<br>◯ Paternal Grandfather<br>◯ Maternal Great Grandmother<br>◯ Maternal Great Grandfather<br>◯ Paternal Great Grandmother<br>◯ Paternal Great Grandfather<br>◯ Family Friend<br>◯ Significant Other (study mother's significant other that is not the infant's father)<br>◯ Other

(9) Household member's relation to the child	◯ Study Infant<br>◯ Study Mother<br>◯ Father<br>◯ Sister<br>◯ Brother<br>◯ Aunt<br>◯ Uncle<br>◯ Cousin<br>◯ Maternal Grandmother<br>◯ Maternal Grandfather<br>◯ Paternal Grandmother<br>◯ Paternal Grandfather<br>◯ Maternal Great Grandmother<br>◯ Maternal Great Grandfather<br>◯ Paternal Great Grandmother<br>◯ Paternal Great Grandfather<br>◯ Family Friend<br>◯ Significant Other (study mother's significant other that is not the infant's father)<br>◯ Other
(10) Household member's relation to the child	◯ Study Infant<br>◯ Study Mother<br>◯ Father<br>◯ Sister<br>◯ Brother<br>◯ Aunt<br>◯ Uncle<br>◯ Cousin<br>◯ Maternal Grandmother<br>◯ Maternal Grandfather<br>◯ Paternal Grandmother<br>◯ Paternal Grandfather<br>◯ Maternal Great Grandmother<br>◯ Maternal Great Grandfather<br>◯ Paternal Great Grandmother<br>◯ Paternal Great Grandfather<br>◯ Family Friend<br>◯ Significant Other (study mother's significant other that is not the infant's father)<br>◯ Other

(11) Household member's relation to the child	◯ Study Infant<br>◯ Study Mother<br>◯ Father<br>◯ Sister<br>◯ Brother<br>◯ Aunt<br>◯ Uncle<br>◯ Cousin<br>◯ Maternal Grandmother<br>◯ Maternal Grandfather<br>◯ Paternal Grandmother<br>◯ Paternal Grandfather<br>◯ Maternal Great Grandmother<br>◯ Maternal Great Grandfather<br>◯ Paternal Great Grandmother<br>◯ Paternal Great Grandfather<br>◯ Family Friend<br>◯ Significant Other (study mother's significant other that is not the infant's father)<br>◯ Other
Including the index child, total number of people that live in the household?	◯ 1<br>◯ 2<br>◯ 3<br>◯ 4<br>◯ 5<br>◯ 6<br>◯ 7<br>◯ 8<br>◯ 9<br>◯ 10<br>◯ 11<br>◯ 12<br>◯ 13<br>◯ 14<br>◯ 15<br>◯ 16<br>◯ 17<br>◯ 18<br>◯ 19<br>◯ 20<br>◯ 21<br>◯ 22<br>◯ 23<br>◯ 24<br>◯ 25<br>◯ 26<br>◯ 27<br>◯ 28<br>◯ 29<br>◯ 30<br>◯ 88 Unknown

Including the index child, total number of people 0-5 years old that live in the household?

◯ 1
◯ 2
◯ 3
◯ 4
◯ 5
◯ 6
◯ 7
◯ 8
◯ 9
◯ 10
◯ 11
◯ 12
◯ 13
◯ 14
◯ 15
◯ 16
◯ 17
◯ 18
◯ 19
◯ 20
◯ 21
◯ 22
◯ 23
◯ 24
◯ 25
◯ 26
◯ 27
◯ 28
◯ 29
◯ 30
◯ 88 Unknown

Total number of people 5-17 years old that live in the household?

- ◯ 0
- ◯ 1
- ◯ 2
- ◯ 3
- ◯ 4
- ◯ 5
- ◯ 6
- ◯ 7
- ◯ 8
- ◯ 9
- ◯ 10
- ◯ 11
- ◯ 12
- ◯ 13
- ◯ 14
- ◯ 15
- ◯ 16
- ◯ 17
- ◯ 18
- ◯ 19
- ◯ 20
- ◯ 21
- ◯ 22
- ◯ 23
- ◯ 24
- ◯ 25
- ◯ 26
- ◯ 27
- ◯ 28
- ◯ 29
- ◯ 30
- ◯ Unknown

Including the study mother, total number of people 18-64 years old that live in the household?

- ◯ 1
- ◯ 2
- ◯ 3
- ◯ 4
- ◯ 5
- ◯ 6
- ◯ 7
- ◯ 8
- ◯ 9
- ◯ 10
- ◯ 11
- ◯ 12
- ◯ 13
- ◯ 14
- ◯ 15
- ◯ 16
- ◯ 17
- ◯ 18
- ◯ 19
- ◯ 20
- ◯ 21
- ◯ 22
- ◯ 23
- ◯ 24
- ◯ 25
- ◯ 26
- ◯ 27
- ◯ 28
- ◯ 29
- ◯ 30
- ◯ 88 Unknown

Total number of people 65 and older that live in the household?	◯ 0<br>◯ 1<br>◯ 2<br>◯ 3<br>◯ 4<br>◯ 5<br>◯ 6<br>◯ 7<br>◯ 8<br>◯ 9<br>◯ 10<br>◯ 11<br>◯ 12<br>◯ 13<br>◯ 14<br>◯ 15<br>◯ 16<br>◯ 17<br>◯ 18<br>◯ 19<br>◯ 20<br>◯ 21<br>◯ 22<br>◯ 23<br>◯ 24<br>◯ 25<br>◯ 26<br>◯ 27<br>◯ 28<br>◯ 29<br>◯ 30<br>◯ Unknown

Pets

Do you currently have any pets in your home?	◯ No<br>◯ Yes<br>◯ Unsure<br>◯ Refused/No answer
Are your pets...	◯ Inside the home only<br>◯ Outside the home only<br>◯ Both inside and outside the home
What kinds of pets do you have? (please check all that apply)	☐ Dog(s)<br>☐ Cat(s)<br>☐ Bird(s)/Chicken(s)<br>☐ Fish<br>☐ Rodent(s)<br>☐ Other
Please specify what other pet(s) you have	__________________________________

Travel

Since [week_0_arm_1][nbcrf_inf_name] was born, have you taken any trips outside of the US?	◯ No<br>◯ Yes<br>◯ Unknown<br>◯ Refused to answer
How many trips outside of the US have you taken since [week_0_arm_1][nbcrf_inf_name] was born?	◯ 1<br>◯ 2<br>◯ 3<br>◯ 4<br>◯ 5<br>◯ More than 5
Date of your first trip (Month/year)<br><br>CRC: please enter MM/01/YY for the date	________________
Did [week_0_arm_1][nbcrf_inf_name] come with you on this trip?	◯ No<br>◯ Yes<br>◯ Unsure<br>◯ Refused
Trip 1: Where did you travel (what country/countries) for this trip?	________________
Date of your second trip (Month/year)<br><br>CRC: please enter MM/01/YY for the date	________________
Did [week_0_arm_1][nbcrf_inf_name] come with you on this trip?	◯ No<br>◯ Yes<br>◯ Unsure<br>◯ Refused
Trip 2: Where did you travel (what country/countries) for this trip?	________________
Date of your third trip (Month/year)<br><br>CRC: please enter MM/01/YY for the date	________________
Did [week_0_arm_1][nbcrf_inf_name] come with you on this trip?	◯ No<br>◯ Yes<br>◯ Unsure<br>◯ Refused
Trip 3: Where did you travel (what country/countries) for this trip?	________________
Date of your fourth trip (Month/year)<br><br>CRC: please enter MM/01/YY for the date	________________
Did [week_0_arm_1][nbcrf_inf_name] come with you on this trip?	◯ No<br>◯ Yes<br>◯ Unsure<br>◯ Refused

Trip 4: Where did you travel (what country/countries) for this trip?	______________________
Date of your fifth trip (Month/year)<br><br>CRC: please enter MM/01/YY for the date	______________________
Did [week_0_arm_1][nbcrf_inf_name] come with you on this trip?	◯ No<br>◯ Yes<br>◯ Unsure<br>◯ Refused
Trip 5: Where did you travel (what country/countries) for this trip?	______________________
Date(s) of any additional trips (Month/year)<br><br>CRC: please enter MM/01/YY for the date	______________________
Where did you travel (what country/countries) for these trips?	______________________
Please list any additional trips that [week_0_arm_1][nbcrf_inf_name] came with you.	______________________

Maternal Occupation

Since [week_0_arm_1][nbcrf_inf_name] was 18 months old, have you worked outside the home?<br><br>Month 18: [month_18_arm_1][m18_workoutsidehome]	◯ No<br>◯ Yes<br>◯ Unsure/Unknown<br>◯ Refused/No Response
How many days have you worked outside the home since [week_0_arm_1][nbcrf_inf_name] was 18 months old?<br><br>Month 18: [month_18_arm_1][m18_daysworkoutsidehome]	______________________
How would you describe your work from home?	◯ Stay at home mom<br>◯ Home based business<br>◯ Work from home/telecommute<br>◯ Unemployed/looking for work<br>◯ Other
Please describe your "other" work from home	______________________
Please briefly describe your duties at this job	______________________
What is your current job title?<br>(If more than one job, list the job where you spend most of your time)	______________________
When did you start this job? (Month & Year)<br><br>CRC: Please enter MM/01/YY for date	______________________

Would you describe this job as...
(check all that apply)

- ☐ A health care organization
- ☐ A school or an educational organization
- ☐ A child care center or home
- ☐ An animal care or veterinary service
- ☐ Restaurant, catering, or food service
- ☐ Customer service, non-food hospitality, or direct sales
- ☐ Self-employed
- ☐ Other

If your job type is "other," please describe:

____

Does your job require you to frequently interact with

	No	Yes	Unknown	Refused to answer
The general public (not your co-workers or staff)	◯	◯	◯	◯
Children between the ages of 0-5	◯	◯	◯	◯
Children between the ages of 6-12	◯	◯	◯	◯
Adolescents/young adults between the ages of 13-21	◯	◯	◯	◯
People between the ages of 22-65	◯	◯	◯	◯
People older than 65	◯	◯	◯	◯
Animals	◯	◯	◯	◯

Since [week_0_arm_1][nbcrf_inf_name]'s birth, have you had different kinds of jobs other than your current job?

- ◯ No
- ◯ Yes
- ◯ Unknown
- ◯ Refused to answer

Please indicate the types of additional jobs you have had since [week_0_arm_1][nbcrf_inf_name] was born:
(Check all that apply)
(CRC: Exclude original answer)

- ☐ A health care organization
- ☐ A school or an educational organization
- ☐ A child care center or home
- ☐ An animal care or veterinary service
- ☐ Restaurant, catering, or food service
- ☐ Customer service, non-food hospitality, or direct sales
- ☐ Self-employed
- ☐ Other

If you said "other," please describe:

____

Did any of these jobs require you to frequently interact with

	No	Yes	Unsure	Refused to answer
The general public (not your co-workers or staff)	◯	◯	◯	◯
Children between the ages of 0-5	◯	◯	◯	◯
Children between the ages of 6-12	◯	◯	◯	◯
Adolescents/young adults between the ages of 13-21	◯	◯	◯	◯
People between the ages of 22-65	◯	◯	◯	◯
People older than 65	◯	◯	◯	◯
Animals	◯	◯	◯	◯

Childcare

Who cares for [week_0_arm_1][nbcrf_inf_name] throughout the day?

Check all that apply

☐ Mother
☐ Child's Father
☐ Adult relative other than the father
☐ Unrelated adult, including licensed caregivers
☐ Children under age 18

If an adult relative, other than the father, cares for [week_0_arm_1][nbcrf_inf_name] throughout the day, please specify the relation to [week_0_arm_1][nbcrf_inf_name]:

____

If a child under age 18 cares for [week_0_arm_1][nbcrf_inf_name] throughout the day, please specify the relation to [week_0_arm_1][nbcrf_inf_name]:

____

Where does the care of [week_0_arm_1][nbcrf_inf_name] occur?

Month 18: [Month_18_arm_1][m18_care_location]

◯ Solely in my home
◯ Out of home child care at least once a week

If out of home child care, which is used most often:

Month 18: [month_18_arm_1][m18_care_outofhome]

◯ In someone else's home
◯ In a child care center/Headstart/Preschool

How many other children are in care in [week_0_arm_1][nbcrf_inf_name]'s group?

Month 18: [month_18_arm_1][m18_care_numberofchildren]

____

How many days per week is [week_0_arm_1][nbcrf_inf_name] in care outside of the home?

Month 18: [month_18_arm_1][m18_care_daysperweek]

____

Infant Health

What is the name of your child's pediatrician and/or the name of the practice?

CRC: Format as "Dr. Name at Practice Name"

Month 18: [month_18_arm_1][m18_pediatrician]
Month 21: [month_21_arm_1][m21_pediatrician_new]

____

Name of pediatric practice

____

Name of physician

(if non-specific, enter "rotating")

____

Since [week_0_arm_1][nbcrf_inf_name] was 18 months old, how many times has he or she been to the doctor's office, clinic or hospital for any reason?

- ◯ 0
- ◯ 1
- ◯ 2
- ◯ 3
- ◯ 4 or more times

Medical Visit #1

(1) Date of visit

____

(1) Date Description:

- ◯ Exact
- ◯ Approximate
- ◯ Unknown
- ◯ No Response/Refused

(1) Location

- ◯ Doctor's Office
- ◯ Clinic
- ◯ ED/Hospital

(1) Reason for visit

____

Medical Visit #2

(2) Date of visit

____

(2) Date Description:

- ◯ Exact
- ◯ Approximate
- ◯ Unknown
- ◯ No Response/Refused

(2) Location

- ◯ Doctor's Office
- ◯ Clinic
- ◯ ED/Hospital

(2) Reason for visit

____

Medical Visit #3

(3) Date of visit	__________________
(3) Date Description:	◯ Exact<br>◯ Approximate<br>◯ Unknown<br>◯ No Response/Refused
(3) Location	◯ Doctor's Office<br>◯ Clinic<br>◯ ED/Hospital
(3) Reason for visit	__________________
If [week_0_arm_1][nbcrf_inf_name] has had 4 or more medical visits since they were six months old, please enter the date, date description, location and reason for visit here:	__________________
Is [week_0_arm_1][nbcrf_inf_name] currently ill?	◯ No<br>◯ Yes<br>◯ Unsure/Unknown<br>◯ Refused/ No Response
What is [week_0_arm_1][nbcrf_inf_name]'s current health problem?	__________________
Since [week_0_arm_1][nbcrf_inf_name] was born, how many days have you taken off of work for respiratory or stomach illness to care for him/her?<br>(Best estimate)	__________________
Were you able to use paid sick leave for these days?	◯ No<br>◯ Yes<br>◯ Unknown<br>◯ Refused to answer

On average, how many bowel movements does [week_0_arm_1][nbcrf_inf_name] typically have each day? If they do not have a bowel movement every day, how many bowel movements does [week_0_arm_1][nbcrf_inf_name] have each week?	◯ Once per DAY<br>◯ Twice per DAY<br>◯ Three times each DAY<br>◯ Four times each DAY<br>◯ Five times each DAY<br>◯ Six times each DAY<br>◯ Seven times each DAY<br>◯ Eight times each DAY<br>◯ Nine times each DAY<br>◯ Ten times each DAY<br>◯ Eleven times each DAY<br>◯ Twelve times each DAY<br>◯ Once per WEEK<br>◯ Twice per WEEK<br>◯ Three times per WEEK<br>◯ Four times per WEEK<br>◯ Five times per WEEK<br>◯ Six times per WEEK

Infant Medication Use

Types of Medications

Vitamins or dietary supplements<br><br>Month 18: [month_18_arm_1][m18_inf_vitamin_supplement]	◯ No<br>◯ Yes<br>◯ Unsure/Unknown<br>◯ Refused/No Response
Over the counter medications<br><br>Month 18: [month_18_arm_1][m18_inf_otcmeds]	◯ No<br>◯ Yes<br>◯ Unsure/Unknown<br>◯ Refused/No Response
Herbal or folk remedies<br><br>Month 18: [month_18_arm_1][m18_inf_herbalremedies]	◯ No<br>◯ Yes<br>◯ Unsure/Unknown<br>◯ Refused/No Response
Prescription medications<br><br>Month 18: [month_18_arm_1][m18_inf_prescriptions]	◯ No<br>◯ Yes<br>◯ Unsure/Unknown<br>◯ Refused/No Response

Infant Medication List

The following questions are about any medications that [week_0_arm_1][nbcrf_inf_name] has taken since he/she was 18 months old

How many vitamins/dietary supplements, over the counter medications, herbal/folk remedies or prescription medications is the infant taking in total?

Month 18: [month_18_arm_1][m18_infantmedications]

- ◯ 1
- ◯ 2
- ◯ 3
- ◯ 4
- ◯ 5
- ◯ 6
- ◯ 7
- ◯ 8
- ◯ 9
- ◯ 10
- ◯ 11 or More

Infant Medication 1

(1) Medication Name

Month 18: [month_18_arm_1][m18_infmed_name1] ________________________________

(1) Dosage/Strength

Month 18: [month_18_arm_1][m18_infmed_dose1] ________________________________

(1) Route

Month 18: [month_18_arm_1][m18_infmed_route1] ________________________________

(1) Units

Month 18: [month_18_arm_1][m18_infmed_units1] ________________________________

(1) Frequency

Month 18: [month_18_arm_1][m18_infmed_freq1] ________________________________

(1) Indication

Month 18: [month_18_arm_1][m18_infmed_indication1] ________________________________

Infant Medication 2

(2) Medication Name

Month 18: [month_18_arm_1][m18_infmed_name2] ________________________________

(2) Dosage/Strength

Month 18: [month_18_arm_1][m18_infmed_dose2] ________________________________

(2) Units

Month 18: [month_18_arm_1][m18_infmed_units2] ________________________________

(2) Route

Month 18: [month_18_arm_1][m18_infmed_route2] ________________________________

(2) Frequency

Month 18: [month_18_arm_1][m18_infmed_freq2] ________________________

(2) Indication

Month 18: [month_18_arm_1][m18_infmed_indication2] ________________________

Infant Medication 3

(3) Medication Name

Month 18: [month_18_arm_1][m18_infmed_name3] ________________________

(3) Dosage/Strength

Month 18: [month_18_arm_1][m18_infmed_dose3] ________________________

(3) Units

Month 18: [month_18_arm_1][m18_infmed_units3] ________________________

(3) Route

Month 18: [month_18_arm_1][m18_infmed_route3] ________________________

(3) Frequency

Month 18: [month_18_arm_1][m18_infmed_freq3] ________________________

(3) Indication

Month 18: [month_18_arm_1][m18_infmed_indication3] ________________________

Infant Medication 4

(4) Medication Name

Month 18: [month_18_arm_1][m18_infmed_name4] ________________________

(4) Dosage/Strength

Month 18: [month_18_arm_1][m18_infmed_dose4] ________________________

(4) Units

Month 18: [month_18_arm_1][m18_infmed_units4] ________________________

(4) Route

Month 18: [month_18_arm_1][m18_infmed_route4] ________________________

(4) Frequency

Month 18: [month_18_arm_1][m18_infmed_freq4] ________________________

(4) Indication

Month 18: [month_18_arm_1][m18_infmed_indication4] ______________________

Infant Medication 5

(5) Medication Name

Month 18: [month_18_arm_1][m18_infmed_name5] ______________________

(5) Dosage/Strength

Month 18: [month_18_arm_1][m18_infmed_dose5] ______________________

(5) Units

Month 18: [month_18_arm_1][m18_infmed_units5] ______________________

(5) Route

Month 18: [month_18_arm_1][m18_infmed_route5] ______________________

(5) Frequency

Month 18: [month_18_arm_1][m18_infmed_freq5] ______________________

(5) Indication

Month 18: [month_18_arm_1][m18_infmed_indication5] ______________________

Infant Medication 6

(6) Medication Name

Month 18: [month_18_arm_1][m18_infmed_name6] ______________________

(6) Dosage/Strength

Month 18: [month_18_arm_1][m18_infmed_dose6] ______________________

(6) Units

Month 18: [month_18_arm_1][m18_infmed_units6] ______________________

(6) Route

Month 18: [month_18_arm_1][m18_infmed_route6] ______________________

(6) Frequency

Month 18: [month_18_arm_1][m18_infmed_freq6] ______________________

(6) Indication

Month 18: [month_18_arm_1][m18_infmed_indication6] ______________________

Infant Medication 7

(7) Medication Name

Month 18: [month_18_arm_1][m18_infmed_name7] ______________________

(7) Dosage/Strength

Month 18: [month_18_arm_1][m18_infmed_dose7] ______________________

(7) Units

Month 18: [month_18_arm_1][m18_infmed_units7] ______________________

(7) Route

Month 18: [month_18_arm_1][m18_infmed_route7] ______________________

(7) Frequency

Month 18: [month_18_arm_1][m18_infmed_freq7] ______________________

(7) Indication

Month 18: [month_18_arm_1][m18_infmed_indication7] ______________________

Infant Medication 8

(8) Medication Name

Month 18: [month_18_arm_1][m18_infmed_name8] ______________________

(8) Dosage/Strength

Month 18: [month_18_arm_1][m18_infmed_dose8] ______________________

(8) Units

Month 18: [month_18_arm_1][m18_infmed_units8] ______________________

(8) Route

Month 18: [month_18_arm_1][m18_infmed_route8] ______________________

(8) Frequency

Month 18: [month_18_arm_1][m18_infmed_freq8] ______________________

(8) Indication

Month 18: [month_18_arm_1][m18_infmed_indication8] ______________________

Infant Medication 9

(9) Medication Name

Month 18: [month_18_arm_1][m18_infmed_name9] ______________________

(9) Dosage/Strength

Month 18: [month_18_arm_1][m18_infmed_dose9] ________________

(9) Units

Month 18: [month_18_arm_1][m18_infmed_units9] ________________

(9) Route

Month 18: [month_18_arm_1][m18_infmed_route9] ________________

(9) Frequency

Month 18: [month_18_arm_1][m18_infmed_freq9] ________________

(9) Indication

Month 18: [month_18_arm_1][m18_infmed_indication9] ________________

Infant Medication 10

(10) Medication Name

Month 18: [month_18_arm_1][m18_infmed_name10] ________________

(10) Dosage/Strength

Month 18: [month_18_arm_1][m18_infmed_dose10] ________________

(10) Units

Month 18: [month_18_arm_1][m18_infmed_units10] ________________

(10) Route

Month 18: [month_18_arm_1][m18_infmed_route10] ________________

(10) Frequency

Month 18: [month_18_arm_1][m18_infmed_freq10] ________________

(10) Indication

Month 18: [month_18_arm_1][m18_infmed_indication10] ________________

If the child is taking more than 10 medications, please record the name, dosage, units, route, frequency and indications of those medications here: ____________________

Infant Anthropometry

Now we are going to take measurements of your child, including length, weight and head & arm circumference.

Preform all measurements AT LEAST TWICE. If there is a discrepancy between measurements 1 and 2, take a third measurement.

LENGTH in centimeters

(1) Length ________________ (centimeters)

(2) Length ________________ (centimeters)

(3) Length
[in case of discrepancy] ________________ (centimeters)

WEIGHT in kilograms

(1) Weight ________________ (kilograms)

(2) Weight ________________ (kilograms)

(3) Weight
[in case of discrepancy] ________________ (kilograms)

HEAD CIRCUMFERENCE in centimeters

(1) Head Circumference ________________ (centimeters)

(2) Head Circumference ________________ (centimeters)

(3) Head Circumference
[in case of discrepancy] ________________ (centimeters)

ARM CIRCUMFERENCE in centimeters

(1) Arm Circumference

____
(centimeters)

(2) Arm Circumference

____
(centimeters)

(3) Arm Circumference

[in case of discrepancy]

____
(centimeters)

If a given measurement could not be assessed, please mark those measuremente here:

- ☐ LENGTH was not assessed
- ☐ WEIGHT was not assessed
- ☐ HEAD CIRCUMFERENCE was not assessed
- ☐ ARM CIRCUMFERENCE was not assessed

(1) Venipuncture

(Initial attempt at month 24 visit)

(1) Amount of blood drawn (milliliters)

- ◯ 0
- ◯ 0.25
- ◯ 0.50
- ◯ 0.75
- ◯ 1.0
- ◯ 1.25
- ◯ 1.50
- ◯ 1.75
- ◯ 2.0
- ◯ 2.25
- ◯ 2.50
- ◯ 2.75
- ◯ 3.0
- ◯ 3.25
- ◯ 3.50
- ◯ 3.75
- ◯ 4.0
- ◯ 4.25
- ◯ 4.50
- ◯ 4.75
- ◯ 5.0
- ◯ 5.25
- ◯ 5.50
- ◯ 5.75
- ◯ 6.0

(mls)

(1) Venipuncture attempts

- ◯ 0
- ◯ 1
- ◯ 2
- ◯ 3
- ◯ 4

(1) Name/names of nurse/nurses who preformed venipuncture

____

(1) Comments

____

(1) Was an adequate blood volume obtained?

◯ Yes
◯ No

(1) Is the mother willing to come back for a second blood draw at a later time?

◯ Yes
◯ No
◯ Maybe - will contact the mother in one week
◯ Did not ask the mother - will contact the mother in one week

(1) Date and time of second blood draw attempt:

____
(Remember to place this date and time on the REDCap Calendar)

(2) Venipuncture

(Redraw)

(2) Amount of blood drawn (milliliters)

◯ 0
◯ 0.25
◯ 0.50
◯ 0.75
◯ 1.0
◯ 1.25
◯ 1.50
◯ 1.75
◯ 2.0
◯ 2.25
◯ 2.50
◯ 2.75
◯ 3.0
◯ 3.25
◯ 3.50
◯ 3.75
◯ 4.0
◯ 4.25
◯ 4.50
◯ 4.75
◯ 5.0
◯ 5.25
◯ 5.50
◯ 5.75
◯ 6.0
(mls)

(2) Venipuncture attempts

◯ 0
◯ 1
◯ 2
◯ 3
◯ 4

(2) Name/names of nurse/nurses who preformed venipuncture

____

(2) Comments	____
(2) Was an adequate blood volume obtained?	◯ Yes<br>◯ No
Unable to contact, attempt by email or letter	◯ Yes<br>◯ No
