# Supplementary material for: Pediatric Respiratory and Enteric Virus Acquisition and Immunogenesis in US Mothers and Children Aged 0-2: PREVAIL Cohort Study
Source: JMIR Res Protoc. 2021 Feb 12;10(2):e22222. doi: 10.2196/22222 (PMC7910118; doi:10.2196/22222)
Supplement: Multimedia Appendix 6 [file resprot_v10i2e22222_app6.pdf]

PREVAIL Study Weekly Survey

Please complete the survey below about [week_0_arm_1][nbcrf_inf_name]'s health in the past week.

If you have any questions or concerns, please contact a PREVAIL Research Coordinator.

In the past week, has [week_0_arm_1][nbcrf_inf_name] experienced fever and/or cough?
◯ Yes
◯ No

On what date did [week_0_arm_1][nbcrf_inf_name]'s cough and/or fever begin?
____
((MM/DD/YYYY))

Is [week_0_arm_1][nbcrf_inf_name] still sick with a cough or fever TODAY?
◯ Yes
◯ No

	Yes	No	Unsure/Unknown
During this illness, has [week_0_arm_1][nbcrf_inf_name] had a cough?	◯	◯	◯
During this illness, has [week_0_arm_1][nbcrf_inf_name] had an earache?	◯	◯	◯
During this illness, has [week_0_arm_1][nbcrf_inf_name] had nasal congestion or a runny nose?	◯	◯	◯
During this illness, has [week_0_arm_1][nbcrf_inf_name] had a sore throat?	◯	◯	◯
During this illness, has [week_0_arm_1][nbcrf_inf_name] had wheezing?	◯	◯	◯
During this illness, has [week_0_arm_1][nbcrf_inf_name] had rapid or shallow breathing/shortness of breath	◯	◯	◯

Has [week_0_arm_1][nbcrf_inf_name] had a fever during this illness?
◯ Yes
◯ No
◯ Unsure/Unknown

Did you measure [week_0_arm_1][nbcrf_inf_name]'s temperature with a thermometer?
◯ Yes
◯ No

What was the highest temperature measured?
____
(record temperature in degrees fahrenheit)

What method did you use to measure this temperature?	◯ Rectal<br>◯ Armpit<br>◯ Mouth<br>◯ Ear<br>◯ Forehead<br>◯ Unsure/Unknown<br>◯ Other

Please collect an additional nasal swab from [week_0_arm_1][nbcrf_inf_name] and send it to CCHMC using the courier. In the coming week during this illness, remember to record [week_0_arm_1][nbcrf_inf_name]'s temperature, symptoms and medications on the log sheet. This information will be useful to you when completing next week's survey.

Please collect an additional nasal swab from [week_0_arm_1][nbcrf_inf_name] and send it to CCHMC using the courier. In the next 24-48 hours, you will receive another text survey to ask you more detailed question about this illness.

In the past week, has [week_0_arm_1][nbcrf_inf_name] experienced vomiting and/or diarrhea?	◯ Yes<br>◯ No

We are glad to hear that [week_0_arm_1][nbcrf_inf_name] is well this week. Please remember to send in one nasal swab and one diaper through the courier. Thank you!

On what date did vomiting and/or diarrhea start for [week_0_arm_1][nbcrf_inf_name]?	______________________<br>(MM/DD/YYYY)
Is [week_0_arm_1][nbcrf_inf_name] still sick with a vomiting and/or diarrhea TODAY?	◯ Yes<br>◯ No
During this illness, has [week_0_arm_1][nbcrf_inf_name] had diarrhea?	◯ Yes<br>◯ No<br>◯ Unsure/Unknown<br>(Please select one)
During this illness, has [week_0_arm_1][nbcrf_inf_name] vomited?	◯ Yes<br>◯ No<br>◯ Unsure/Unknown<br>(Please select one)
When you pinched the skin on your child's abdomen to test for dehydration, how did your child's skin retract?	◯ Normally - skin retracts immediately<br>◯ Slowly - the fold is visible for less than 2 seconds<br>◯ Very Slowly-the fold is visible for more than seconds<br>◯ Unsure/Unknown
Has [week_0_arm_1][nbcrf_inf_name] had a fever during this illness?	◯ Yes<br>◯ No<br>◯ Unsure/Unknown<br>(Please select one)
Did you measure [week_0_arm_1][nbcrf_inf_name]'s temperature with a thermometer?	◯ Yes<br>◯ No
What was the highest temperature measured?	______________________<br>(record temperature in degrees fahrenheit)

What method did you use to measure this temperature?	◯ Rectal<br>◯ Armpit<br>◯ Mouth<br>◯ Ear<br>◯ Forehead<br>◯ Unsure/Unknown<br>◯ Other<br>(Select all that apply)

Please collect an additional stool sample from [week_0_arm_1][nbcrf_inf_name] and send it to CCHMC using the courier. In the coming week during this illness, remember to record [week_0_arm_1][nbcrf_inf_name]'s temperature, symptoms and medications on the log sheet. This information will be useful to you when completing next week's survey.

Please collect an additional stool sample from [week_0_arm_1][nbcrf_inf_name] and send it to CCHMC using the courier. In the next 24-48 hours, you will receive another text survey to ask you more detailed questions about this illness.

Was this week missed?<br><br>(Not completed by the subject via text or by the CRC via telephone call to the subject)	◯ Yes<br>◯ No<br>◯ Not Applicable
Was this survey completed by the CRC via telephone call to the subject?	◯ Yes<br>◯ No
CRC Initials	__________________________
